# Association of Low- and No-Calorie Sweetened Beverages as a Replacement for Sugar-Sweetened Beverages With Body Weight and Cardiometabolic Risk: A Systematic Review and Meta-analysis

**DOI:** 10.1001/jamanetworkopen.2022.2092

**Published:** 2022-03-14

**Authors:** Néma D. McGlynn, Tauseef Ahmad Khan, Lily Wang, Roselyn Zhang, Laura Chiavaroli, Fei Au-Yeung, Jennifer J. Lee, Jarvis C. Noronha, Elena M. Comelli, Sonia Blanco Mejia, Amna Ahmed, Vasanti S. Malik, James O. Hill, Lawrence A. Leiter, Arnav Agarwal, Per B. Jeppesen, Dario Rahelić, Hana Kahleová, Jordi Salas-Salvadó, Cyril W. C. Kendall, John L. Sievenpiper

**Affiliations:** 1 Department of Nutritional Sciences, Temerty Faculty of Medicine, University of Toronto, Toronto, Ontario, Canada; 2 Toronto 3D Knowledge Synthesis and Clinical Trials Unit, Clinical Nutrition and Risk Factor Modification Centre, St Michael's Hospital, Toronto, Ontario, Canada; 3 Applied Human Nutrition, Mount Saint Vincent University, Halifax, Nova Scotia, Canada; 4 Faculty of Medicine, School of Medicine, The University of Queensland, Brisbane, Australia; 5 Department of Nutrition, Harvard T.H. Chan School of Public Health, Boston, Massachusetts; 6 Department of Nutrition Sciences, The University of Alabama at Birmingham, Birmingham; 7 Division of Endocrinology and Metabolism, Department of Medicine, St Michael’s Hospital, Toronto, Ontario, Canada; 8 Li Ka Shing Knowledge Institute, St Michael’s Hospital, Toronto, Ontario, Canada; 9 Department of Medicine, Temerty Faculty of Medicine, University of Toronto, Toronto, Ontario, Canada; 10 Division of General Internal Medicine, Department of Medicine, McMaster University, Hamilton, Ontario, Canada; 11 Department of Clinical Medicine, Aarhus University, Aarhus University Hospital, Aarhus, Denmark; 12 Vuk Vrhovac University Clinic for Diabetes, Endocrinology and Metabolic Diseases, Merkur University Hospital, Zagreb, Croatia; 13 University of Zagreb School of Medicine, Zagreb, Croatia; 14 University of Osijek School of Medicine, Osijek, Croatia; 15 Institute for Clinical and Experimental Medicine, Diabetes Centre, Prague, Czech Republic; 16 Physicians Committee for Responsible Medicine, Washington, DC; 17 Universitat Rovira i Virgili, Human Nutrition Department, Institut d'Investigació Sanitària Pere Virgili, Reus, Spain; 18 Centro de Investigación Biomédica en Red de Fisiopatología de la Obesidad y Nutrición (CIBERObn), Instituto de Salud Carlos III, Madrid, Spain; 19 College of Pharmacy and Nutrition, University of Saskatchewan, Saskatoon, Saskatchewan, Canada

## Abstract

**Importance:**

There are concerns that low- and no-calorie sweetened beverages (LNCSBs) do not have established benefits, with major dietary guidelines recommending the use of water and not LNCSBs to replace sugar-sweetened beverages (SSBs). Whether LNCSB as a substitute can yield similar improvements in cardiometabolic risk factors vs water in their intended substitution for SSBs is unclear.

**Objective:**

To assess the association of LNCSBs (using 3 prespecified substitutions of LNCSBs for SSBs, water for SSBs, and LNCSBs for water) with body weight and cardiometabolic risk factors in adults with and without diabetes.

**Data Sources:**

Medline, Embase, and the Cochrane Central Register of Controlled Trials were searched from inception through December 26, 2021.

**Study Selection:**

Randomized clinical trials (RCTs) with at least 2 weeks of interventions comparing LNCSBs, SSBs, and/or water were included.

**Data Extraction and Synthesis:**

Data were extracted and risk of bias was assessed by 2 independent reviewers. A network meta-analysis was performed with data expressed as mean difference (MD) or standardized mean difference (SMD) with 95% CIs. The GRADE (Grading of Recommendations Assessment, Development and Evaluation) system was used to assess the certainty of the evidence.

**Main Outcomes and Measures:**

The primary outcome was body weight. Secondary outcomes were other measures of adiposity, glycemic control, blood lipids, blood pressure, measures of nonalcoholic fatty liver disease, and uric acid.

**Results:**

A total of 17 RCTs with 24 trial comparisons were included, involving 1733 adults (mean [SD] age, 33.1 [6.6] years; 1341 women [77.4%]) with overweight or obesity who were at risk for or had diabetes. Overall, LNCSBs were a substitute for SSBs in 12 RCTs (n = 601 participants), water was a substitute for SSBs in 3 RCTs (n = 429), and LNCSBs were a substitute for water in 9 RCTs (n = 974). Substitution of LNCSBs for SSBs was associated with reduced body weight (MD, −1.06 kg; 95% CI, −1.71 to –0.41 kg), body mass index (MD, −0.32; 95% CI, −0.58 to –0.07), percentage of body fat (MD, −0.60%; 95% CI, −1.03% to –0.18%), and intrahepatocellular lipid (SMD, −0.42; 95% CI, −0.70 to –0.14). Substituting water for SSBs was not associated with any outcome. There was also no association found between substituting LNCSBs for water with any outcome except glycated hemoglobin A_1c_ (MD, 0.21%; 95% CI, 0.02% to 0.40%) and systolic blood pressure (MD, −2.63 mm Hg; 95% CI, −4.71 to −0.55 mm Hg). The certainty of the evidence was moderate (substitution of LNCSBs for SSBs) and low (substitutions of water for SSBs and LNCSBs for water) for body weight and was generally moderate for all other outcomes across all substitutions.

**Conclusions and Relevance:**

This systematic review and meta-analysis found that using LNCSBs as an intended substitute for SSBs was associated with small improvements in body weight and cardiometabolic risk factors without evidence of harm and had a similar direction of benefit as water substitution. The evidence supports the use of LNCSBs as an alternative replacement strategy for SSBs over the moderate term in adults with overweight or obesity who are at risk for or have diabetes.

## Key Points

**Question:**

Are low- and no-calorie sweetened beverages (LNCSBs) as the intended substitute for sugar-sweetened beverages (SSBs) associated with improved body weight and cardiometabolic risk factors similar to water replacement?

**Findings:**

In this systematic review and meta-analysis of 17 randomized clinical trials, LNCSBs as a substitute for SSBs were associated with reduced body weight, body mass index, percentage of body fat, and intrahepatocellular lipid, providing benefits that were similar to those of water, the standard-of-care substitution.

**Meaning:**

The findings of this study suggest that over the moderate term, LNCSBs are a viable alternative to water as a replacement strategy in adults with overweight or obesity who are at risk for or have diabetes.

## Teaser

This systematic review and meta-analysis assesses the outcomes of 3 types of substitution for sugar-sweetened beverages in adults with overweight or obesity and with or without diabetes.

## Introduction

Sugar consumption has emerged as an important public health concern. The evidence on this concern derives largely from consumption of sugar-sweetened beverages (SSBs), with excess intake of SSBs associated with weight gain and downstream cardiometabolic complications.^1,2,3,4^ Sugar-sweetened beverages have been identified as an important public health target.^5,6^ It is unclear whether low- and no-calorie sweetened beverages (LNCSBs) as a replacement strategy for SSBs provide the intended benefits. Recent systematic reviews and meta-analyses^7^ have shown an association between LNCSBs and a higher risk of the conditions that they are intended to prevent, such as weight gain, diabetes, and cardiovascular disease, in prospective cohort studies^8^ and have reported inconsistent findings for weight loss and improvements in downstream cardiometabolic risk factors in randomized clinical trials (RCTs).^7,8^ Biological mechanisms involving impaired sensory and endocrine signaling that was mediated by the sweet taste receptor^9,10^ and changes to the microbiome^10,11^ have been implicated in support of these observations.

Methodological considerations, however, have been raised that limit the inferences that can be drawn from these data. The available prospective cohort studies are at high risk for reverse causality.^12,13,14^ Furthermore, the syntheses of RCTs do not fully account for the calories available to be displaced by LNCSBs, with caloric (eg, SSBs) and noncaloric (eg, water and placebo) comparators that are pooled together or with noncaloric comparators that are used as the sole comparator, leading to an underestimation of the outcome of LNCSBs.^12,13,14^

The prevailing uncertainties have led to mixed recommendations from authoritative bodies. Neither the Dietary Guidelines for Americans nor Canada’s Food Guide supports the use of LNCSBs, and instead both recommend replacing SSBs with water.^5,6^ The American Heart Association supports a narrow indication for LNCSBs, recommending that LNCSBs should be used as a replacement by only adults who are habitual consumers of SSBs, but emphasizing the use of water or an unsweetened alternative.^15^ Similarly, diabetes associations in the UK, US, and Canada support LNCSBs insofar as they are used to displace calories from sugars and SSBs.^16,17,18^ The European Association for the Study of Diabetes has not made any specific recommendations about low- and no-calorie sweeteners (LNCSs) or LNCSBs.^19^ To update the recommendations of the European Association for the Study of Diabetes, the Diabetes and Nutrition Study Group commissioned the present systematic review and meta-analysis to summarize the evidence from RCTs of the association of LNCSBs, the most important source of LNCSs in a diet and a single food matrix, with intermediate cardiometabolic outcomes.^20^ Because of the importance of the comparator in drawing inferences about LNCSBs, we conducted network meta-analyses rather than traditional pairwise meta-analyses to assess the association of LNCSBs with body weight and cardiometabolic risk factors in adults with and without diabetes. We used 3 prespecified substitutions: LNCSBs for SSBs (intended substitution with caloric displacement), water for SSBs (standard-of-care substitution with caloric displacement), and LNCSBs for water (reference substitution without caloric displacement).

## Methods

This systematic review and network meta-analysis was conducted according to the *Cochrane Handbook for Systematic Reviews of Interventions*^21^ and the Preferred Reporting Items for Systematic Reviews and Meta-analyses (PRISMA) reporting guideline.^22^ The protocol is registered at ClinicalTrials.gov (NCT02879500).

### Data Sources, Searches, and Study Selection

We searched Medline, Embase, and the Cochrane Central Register of Controlled Trials from inception through December 26, 2021. Briefly, for this search, we used variations of the exposure terms (LNCSBs and SSBs), outcome terms (adiposity, glycemia, blood lipids, blood pressure [BP], nonalcoholic fatty liver disease [NAFLD], and uric acid), and study design terms (randomized controlled trial, randomized, and placebo). The full search strategy is presented in eTables 1 to 3 in the Supplement. Manual searches of the reference lists of included studies and reviews were also performed.

eTable 4 in the Supplement shows the PICOTS (Population, Intervention, Comparator, Outcome, Time, and Study) framework.^22^ We included RCTs of at least 2 weeks that investigated the association of LNCSBs, SSBs, and/or water with cardiometabolic risk factors. We excluded trials that had multimodal interventions, did not use comparator groups containing at least 1 of the other beverage interventions, included children and pregnant or breastfeeding women, or did not provide viable outcome data. Trials of LNCSs in fortified or nutrient-dense beverages (eg, milk and juice) were also excluded because of the presence of other nutrients.

### Data Extraction, Risk of Bias Assessment, and Outcomes

Two independent reviewers (N.D.M. and R.Z.) extracted relevant data from each included report (eMethods in the Supplement). Additional information was requested from study authors when necessary. Race and ethnicity data were not collected because the available data were not presented by these variables.

The same independent reviewers (N.D.M. and R.Z.) assessed risk of bias for each included RCT using the Cochrane risk-of-bias tool.^23^ Five domains of bias were assessed: sequence generation, allocation concealment, blinding of participants and personnel, incomplete outcome data, and selective reporting. Disagreements between the reviewers were resolved by consensus.

The primary outcome was body weight. Secondary outcomes were other measures of adiposity (body mass index [BMI], which was calculated as weight in kilograms divided by height in meters squared; percentage of body fat; and waist circumference), glycemic control (glycated hemoglobin A_1c_ [HbA_1c_], fasting plasma glucose, 2-hour postprandial glucose during a 75-g oral glucose tolerance test, fasting plasma insulin [FPI], and homeostatic model assessment of insulin resistance), blood lipids (low-density lipoprotein cholesterol, non–high-density lipoprotein cholesterol, triglycerides, high-density lipoprotein cholesterol, and total cholesterol), BP (systolic BP and diastolic BP), measures of NAFLD (intrahepatocellular lipid [IHCL], alanine aminotransferase, and aspartate aminotransferase), and uric acid. Change differences were preferred over end differences. Missing variance data were calculated using established formulas.^21^

### Data Synthesis and Grading the Evidence

This network meta-analysis was based on a frequentist framework and was conducted using the network suite of commands in Stata, version 15 (StataCorp LLC). We used change from baseline values from each study to calculate the mean differences (MDs) between treatments for each substitution (LNCSBs for SSBs, water for SSBs, and LNCSBs for water); otherwise, we used postintervention values (eMethods and eData 1-20 in the Supplement). We performed random-effects network meta-analyses for each outcome to compare the 3 interventions (LNCSBs, SSBs, and water) simultaneously. Inconsistency was assessed in the direct, indirect, and network estimates. We assessed interstudy heterogeneity in the direct (pairwise) estimates using the Cochran *Q* statistic with quantification by the *I*^2^ statistic, where *I*^2 ^≥50% and *P* < .10 were considered to be substantial interstudy heterogeneity. We measured incoherence in the network estimates using both local (loop-specific and side-splitting) and global (design-by-treatment interaction model) approaches.^24,25,26^ If 10 or more trials were available, we conducted a priori subgroup analyses by age, study duration, type of design, disease status, risk of bias, and funding source. Indirectness was assessed in the indirect comparisons by evaluation of intransitivity across the pairwise comparisons comprising the indirect estimates for the study characteristics of age, study length, sample size, and percentage of male participants. Publication bias was assessed if 10 or more trial comparisons were available; we used comparison-adjusted funnel plots to assess funnel plot asymmetry.^24^

We assessed the certainty of the evidence using the Grading of Recommendations Assessment, Development and Evaluation (GRADE) system.^20,27,28,29,30^ Network estimates of RCTs and the direct and indirect estimates that composed these network estimates started at a high certainty of evidence but were downgraded by established criteria for risk of bias, inconsistency (incoherence), indirectness, imprecision, and publication bias (eMethods in the Supplement).

## Results

**Figure 1.**
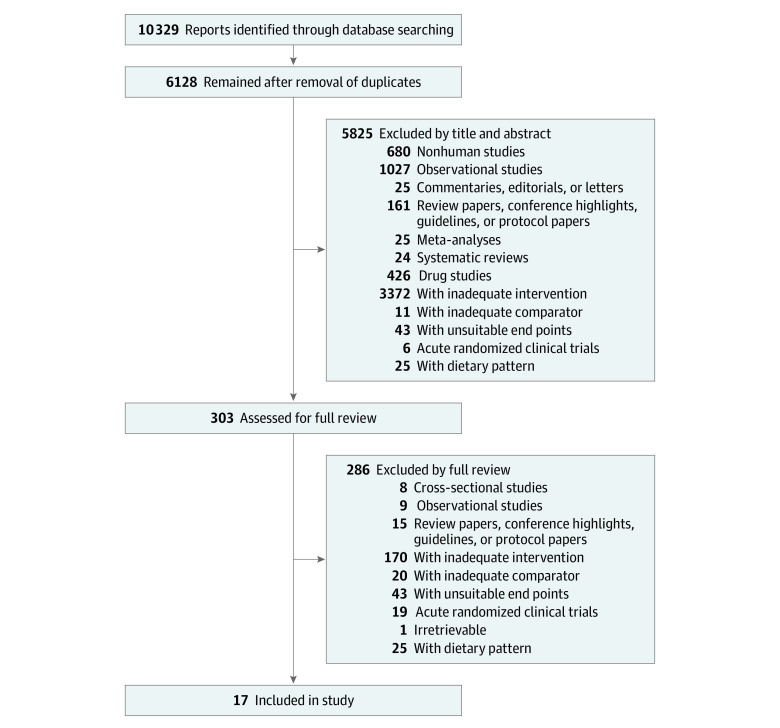
Literature Search for Randomized Clinical Trials of Low- and No-Calorie Sweetened Beverages

Figure 1 shows the flow of the literature search and selection, and eFigures 27 to 46 in the Supplement show the network diagram for each outcome. We identified 4541 reports, of which 13 met the eligibility criteria. An additional 4 reports were found through manual searching. A total of 17 RCTs with 24 trial comparisons were included that assessed the association of the 3 prespecified substitutions with body weight, other measures of adiposity, and cardiometabolic risk.^31,32,33,34,35,36,37,38,39,40,41,42,43,44,45,46,47^ These RCTs involved 1733 adult participants (mean [SD] age, 33.1 [6.6] years; 1341 women [77.4%] and 392 men [22.6%]) with overweight or obesity who were at risk for or had diabetes.

The Table and eTable 5 in the Supplement provide key trial characteristics.^31,32,33,34,35,36,37,38,39,40,41,42,43,44,45,46,47^ Overall, the RCTs had a medium sample size, with a median (range) number of 72 (27-308) participants, and involved more women than men (23% men to 77% women). Most participants were younger (median [range] age, 34 [23-48] years) and had overweight or obesity (median [range] BMI, 31 [22-36]), with 9 trials^31,32,33,34,35,36,38,39,40,41,42,44,45,46^ that included only participants with overweight and/or obesity and 1 trial^40^ that included participants with type 2 diabetes.

**Table.** Trial Characteristics

Source and country	Total No. of participants	Population	Age, mean (SD), y	No. of participants by sex (%)	LNCS type	Beverage dosage, mL/d			Design	Duration, wk	Funding source
						LNCSB	Water	SSB			
Bonnet et al,^31^ 2018; France	50	Overweight or healthy weight, otherwise healthy, non- or low-LNCS consumers	31.1 (10.3)	Male: 22 (44) Female: 28 (56)	Aspartame or acesulfame potassium	660	660	NA	Crossover	12	Agency or industry
Bruun et al,^32^ 2015; Denmark^a^	35	Overweight or obese, otherwise healthy	39 (1.1)	Male: 14 (40) Female: 21 (60)	Aspartame	1000	1000	1000	Parallel	26	Agency or industry
Campos et al,^34^ 2015; Switzerland	27	Overweight or obese, otherwise healthy, regular SSB consumers	NR	Male: 14 (52) Female: 13 (48)	NR	1300	NA	1300	Parallel	12	Agency
Ebbeling et al,^33^ 2020; US	203	Overweight or obese, otherwise healthy, regular SSB consumers	27 (5.6)	Male: 121 (60) Female: 82 (40)	NR	355	355	355	Parallel	52	Agency
Engel et al,^35^ 2018; Denmark^a^	45	Overweight or obese, otherwise healthy	38.6 (7.6)	Male: 16 (36) Female: 29 (64)	Aspartame	1000	1000	1000	Parallel	26	Agency or industry
Hernández-Cordero et al,^36^ 2014; Mexico	240	Overweight or obese, otherwise healthy, regular SSB consumers	33.3 (6.7)	Male: 0 Female: 240 (100)	NR	NA	≥250	≥250	Parallel	39	Industry
Higgins et al,^37^ 2018; US	93	Healthy weight, healthy, non- or low-LNCS consumers	22.9 (1.0)	Male: 43 (46) Female: 50 (54)	Aspartame	500	500	NA	Parallel	12	Industry
Higgins and Mattes,^38^ 2019; US	154	Overweight or obese, otherwise healthy, non- or low-LNCS consumers	27.3 (9.6)	Male: 67 (44) Female: 87 (56)	Saccharin, aspartame, rebaudioside A, or sucralose	1250-1750	NA	1250-1750	Parallel	12	Agency
Madjd et al,^39^ 2015; Iran	62	Overweight or obese, otherwise healthy, regular LNCSB consumers	32 (6.9)	Male: 0 Female: 62 (100)	NR	≥250	≥250	NA	Parallel	24	Agency
Madjd et al,^40^ 2017; Iran	81	Obese, with type 2 diabetes (only on metformin to control diabetes), regular LNCSB consumers	34.8 (7.2)	Male: 0 Female: 81 (100)	NR	≥250	≥250	NA	Parallel	24	Agency
Maersk et al,^41^ 2012; Denmark	35	Overweight or obese, otherwise healthy	39 (26)	Male: 14 (40) Female: 21 (60)	Aspartame	1000	1000	1000	Parallel	26	Agency or industry
Peters et al,^42^ 2016; US	308	Overweight or obese, otherwise healthy, weight stable, regular LNCSB consumers	47.8 (10.5)	Male: 53 (17) Female: 255 (83)	NR	710	710	NA	Parallel	52	Industry
Reid et al,^43^ 2007; England	133	Healthy weight, weight watchers and nonweight watchers	31.8 (9.1)	Male: 0 Female: 133 (100)	Aspartame	1000	NA	1000	Parallel	4	Agency
Reid et al,^44^ 2010; Scotland	53	Overweight, otherwise healthy	33.7 (9.9)	Male: 0 Female: 53 (100)	Aspartame	1000	NA	1000	Parallel	4	Agency
Reid et al,^45^ 2014; Scotland	41	Obese, otherwise healthy	35 (9.1)	Male: 0 Female: 41 (100)	Aspartame	1000	NA	1000	Parallel	4	Agency or industry
Tate et al,^46^ 2012; US	213	Overweight or obese, otherwise healthy, regular SSB consumers	42 (10.7)	Male: 35 (52) Female: 178 (48)	NR	1420-2000	1420-2000	NA	Parallel	26	Industry
Tordoff and Alleva,^47^ 1990; US	30	Healthy weight, healthy	25.6 (5.3)	Male: 21 (70) Female: 9 (30)	Aspartame	1135	NA	1135	Crossover	3	Agency

Abbreviations: LNCS, low- and no-calorie sweetener; LNCSB, low- and no-calorie sweetened beverage; NA, not applicable; NR, not reported; SSB, sugar-sweetened beverage.

^a^ Secondary analyses to Maersk et al.^41^ As more outcomes were reported in the Engel et al^35^ analysis, data from that trial were used for most outcomes, except for uric acid (Bruun et al^32^) and intrahepatocellular lipid (Maersk et al^41^).

Only 8 trials (11 comparisons)^31,32,35,37,38,43,44,45,47^ reported the type of LNCS used in the LNCSBs: 7 comparisons for aspartame and 1 comparison each for aspartame and acesulfame potassium blend, saccharin, rebaudioside A, and sucralose. Overall, LNCSBs were a substitute for SSBs in 12 trials (n = 601 participants),^33,34,35,38,43,44,45,47^ water was a substitute for SSBs in 3 trials (n = 429),^33,35,36,41^ and LNCSBs were a substitute for water in 9 trials (n = 974).^31,33,35,37,39,40,42,46^ The median (range) dosages were 1000 (250-2000) mL per day for LNCSBs, 1000 (250-1750) mL per day for SSBs, and 580 (250- 2000) mL per day for water.

Fifteen trials^32,33,34,35,36,37,38,39,40,41,42,43,44,45,46^ had a parallel design, and 2 trials^31,47^ had a crossover design. Most RCTs were conducted in Europe (n = 8) and North America (n = 6). The median (range) duration of follow-up was 12 (3-52) weeks. Eight trials^33,34,38,39,40,43,44,47^ were funded by agencies (government, not-for-profit health agency, or university sources), 4 trials^36,37,42,46^ were funded by industry, and 5 trials^31,32,35,41,45^ were funded by a combination of agency and industry. We contacted the authors of 7 studies^31,32,34,35,38,44,45^ for additional data, and the authors of 2 studies^34,38^ provided additional data.

eFigures 1 and 2 in the Supplement provide the Cochrane risk-of-bias tool assessments. Eight trial comparisons^32,34,35,37,41,43,44,47^ received an unclear risk-of-bias rating, and 11 comparisons^31,33,36,38,39,40,42,46^ were rated as having a low risk of bias. No RCTs were identified as having a high risk of bias, with no evidence of serious summary risk of bias across the trials.

### Associations of the Prespecified Substitutions 

**Figure 2.**
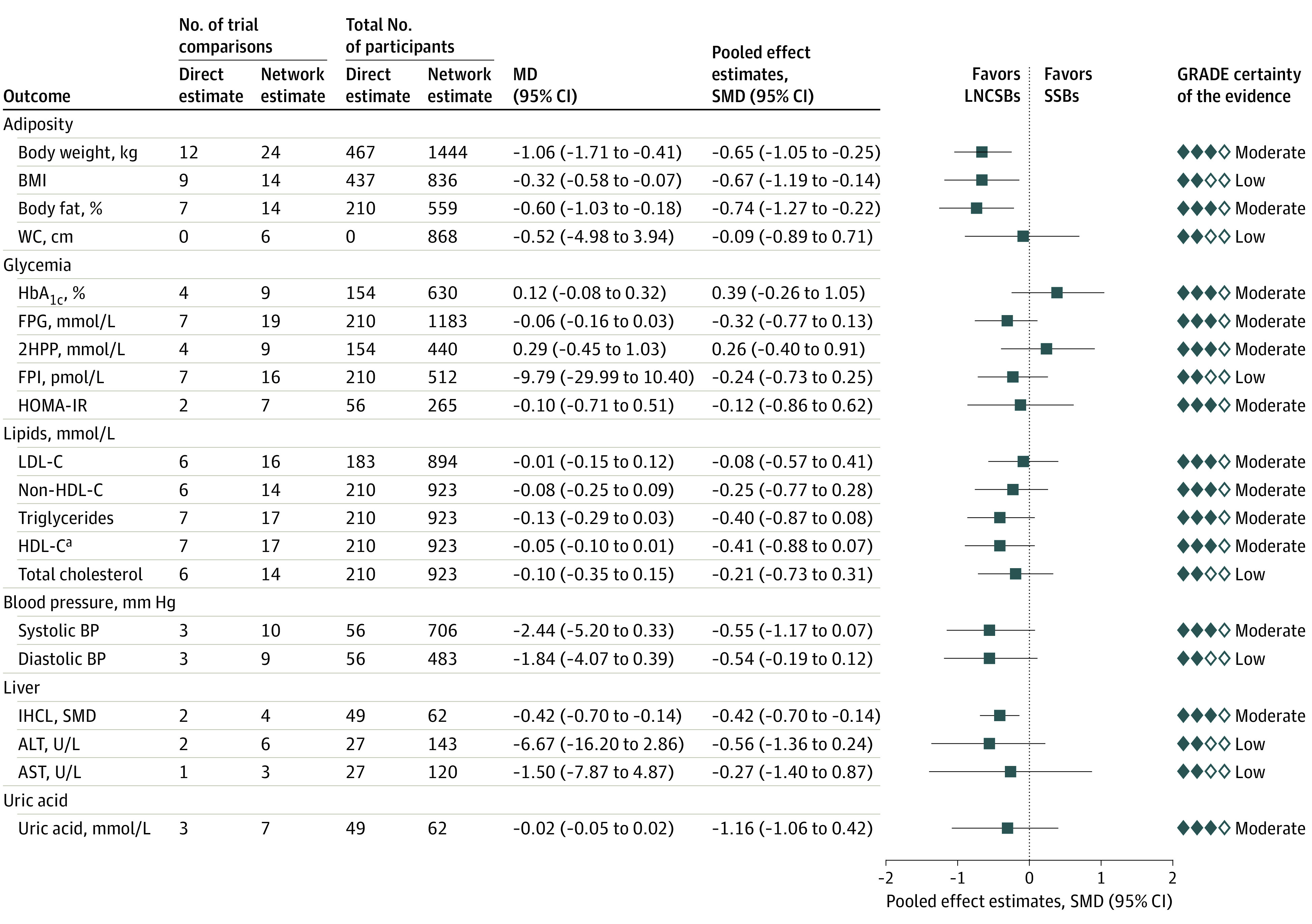
Substitution of Low- and No-Calorie Sweetened Beverages (LNCSBs) for Sugar-Sweetened Beverages (SSBs) Data were pooled using network random-effects models and expressed as mean differences (MDs) and 95% CIs. To display the results for outcomes on the same plot, standardized mean differences (SMDs, represented by blue squares) and pseudo 95% CIs (represented by black horizontal lines and proportionally scaled to the 95% CIs of the MDs) were calculated. 2HPP indicates 2-hour postprandial glucose; ALT, alanine aminotransferase (to convert to μkat/L, multiply by 0.0167); AST, aspartate aminotransferase (to convert to μkat/L, multiply by 0.0167); BMI, body mass index; FPG; fasting plasma glucose; FPI, fasting plasma insulin; GRADE, Grading of Recommendations Assessment, Development and Evaluation; HbA_1c_; glycated hemoglobin A_1c_; HDL-C, high-density lipoprotein cholesterol; HOMA-IR, homeostatic model assessment of insulin resistance; IHCL, intrahepatocellular lipid; LDL-C, low-density lipoprotein cholesterol; and WC, waist circumference. ^a^HDL-C result has been reversed for display purposes; that is, a negative MD would mean a positive improvement.

Figure 2 shows the network meta-analyses of the association of the intended substitution of LNCSBs for SSBs with body weight, other measures of adiposity, and cardiometabolic risk factors. This substitution was associated with reduced body weight (MD, −1.06 kg; 95% CI, −1.71 to –0.41 kg) and lower BMI (MD, −0.32; 95% CI, −0.58 to –0.07), percentage of body fat (MD, −0.60%; 95% CI, −1.03% to –0.18%), and IHCL (standardized MD [SMD], −0.42; 95% CI, −0.70 to –0.14). No other outcomes had significant differences.

**Figure 3.**
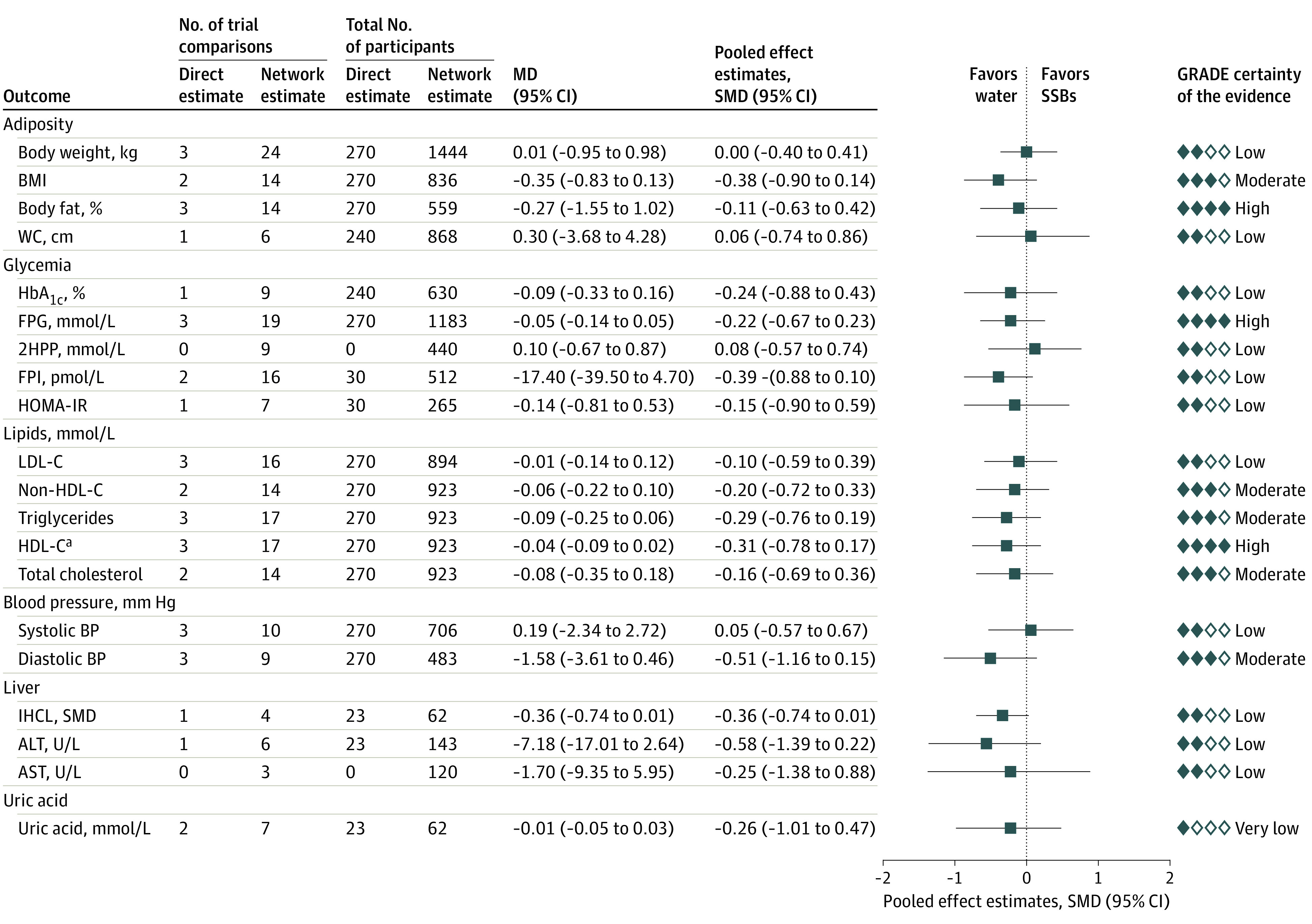
Substitution of Water for Sugar-Sweetened Beverages (SSBs) Data were pooled using network random-effects models and expressed as mean differences (MDs) and 95% CIs. To display the results for outcomes on the same plot, standardized mean differences (SMDs, represented by blue squares) and pseudo 95% CIs (represented by black horizontal lines and proportionally scaled to the 95% CIs of the MDs) were calculated. 2HPP indicates 2-hour postprandial glucose; ALT, alanine aminotransferase (to convert to μkat/L, multiply by 0.0167); AST, aspartate aminotransferase (to convert to μkat/L, multiply by 0.0167); BMI, body mass index; FPG; fasting plasma glucose; FPI, fasting plasma insulin; GRADE, Grading of Recommendations Assessment, Development and Evaluation; HbA_1c_; glycated hemoglobin A_1c_; HDL-C, high-density lipoprotein cholesterol; HOMA-IR, homeostatic model assessment of insulin resistance; IHCL, intrahepatocellular lipid; LDL-C, low-density lipoprotein cholesterol; and WC, waist circumference. ^a^HDL-C result has been reversed for display purposes; that is, a negative MD would mean a positive improvement.

Figure 3 shows the network meta-analyses of the association of the standard-of-care substitution of water for SSBs with body weight, other measures of adiposity, and cardiometabolic risk factors. Neither the primary outcome of body weight (MD, −0.01 kg; 95% CI, −0.95 to 0.98 kg) nor any of the secondary outcomes showed significant differences, although the direction of association favored water for most of the outcomes.

**Figure 4.**
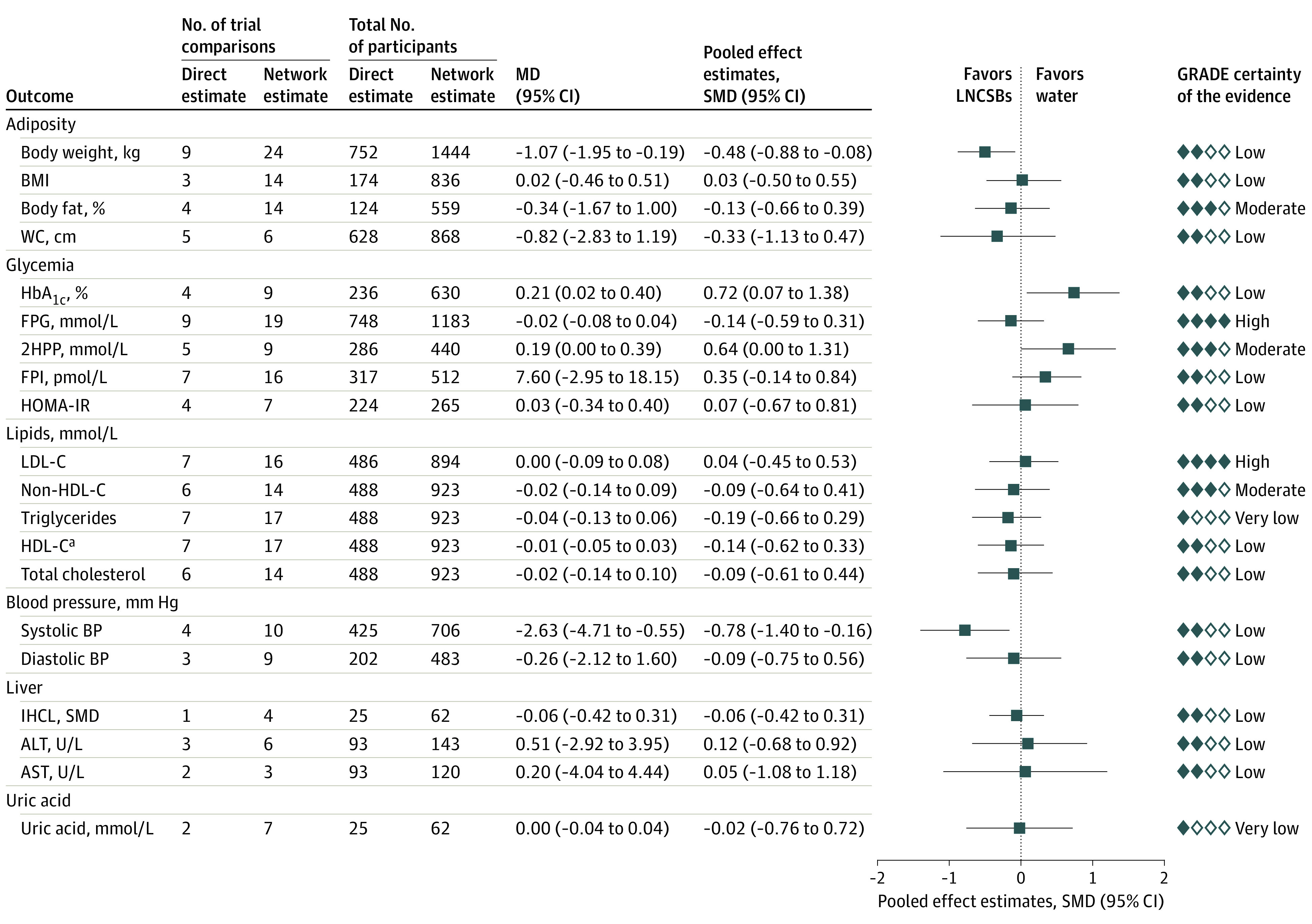
Substitution of Low- and No-Calorie Sweetened Beverages (LNCSBs) for Water Data were pooled using network random-effects models and expressed as mean differences (MDs) and 95% CIs. To display the results for outcomes on the same plot, standardized mean differences (SMDs, represented by blue squares) and pseudo 95% CIs (represented by black horizontal lines and proportionally scaled to the 95% CIs of the MDs) were calculated. 2HPP indicates 2-hour postprandial glucose; ALT, alanine aminotransferase (to convert to μkat/L, multiply by 0.0167); AST, aspartate aminotransferase (to convert to μkat/L, multiply by 0.0167); BMI, body mass index; FPG; fasting plasma glucose; FPI, fasting plasma insulin; GRADE, Grading of Recommendations Assessment, Development and Evaluation; HbA_1c_; glycated hemoglobin A_1c_; HDL-C, high-density lipoprotein cholesterol; HOMA-IR, homeostatic model assessment of insulin resistance; IHCL, intrahepatocellular lipid; LDL-C, low-density lipoprotein cholesterol; and WC, waist circumference. ^a^HDL-C result has been reversed for display purposes; that is, a negative MD would mean a positive improvement.

Figure 4 shows the network analyses of the association of the reference substitution of LNCSBs for water with body weight, other measures of adiposity, and cardiometabolic risk factors. Greater reduction in body weight (MD, −1.07 kg; 95% CI, −1.95 to −0.19 kg) was associated with LCSBs compared with water. Among secondary outcomes, water compared with LNCSBs was associated with lower level of HbA_1c_ (MD, 0.21%; 95% CI, 0.02% to 0.40%), and LNCSBs compared with water were associated with a greater decrease in systolic BP (MD, −2.63 mm Hg; 95% CI, −4.71 to −0.55 mm Hg). No secondary outcomes were affected.

### Adverse Events and Inconsistency

Adverse events were reported in 4 trials,^33,36,43,44^ including tiredness, mood swings, headaches, body aches, nausea, hospitalizations, and weight gain. In all cases, the adverse events were not observed,^43,44^ deemed to be unrelated to the intervention,^33^ or not severe enough to be of consequence.^36^

eTables 6 and 7 in the Supplement show the loop-specific and the design-by-treatment assessment of inconsistency (incoherence) in the network estimates. No significant incoherence was observed by any approach across the 3 substitutions.

eFigures 7 to 26 in the Supplement provide the assessments of network, direct and indirect estimates, inconsistency (heterogeneity) in the direct estimates, and inconsistency (incoherence) between the direct and indirect estimates using side-splitting method. There was evidence of substantial heterogeneity (*I*^2^≥50%; *P* < .10) in the direct pairwise estimates of the association of LNCSBs as a substitute for water with the primary outcome of body weight and secondary outcomes of waist circumference, HbA_1c_, FPI, homeostatic model assessment of insulin resistance, and triglycerides. Incoherence was not significant for any comparison, but on visual inspection slight instability between direct and indirect measures was present for BMI, percentage of body fat, HbA_1c_, fasting blood glucose, FPI, homeostatic model assessment of insulin resistance, low-density lipoprotein cholesterol, triglycerides, high-density lipoprotein cholesterol, total cholesterol, systolic BP, diastolic BP, IHCL, alanine aminotransferase, aspartate aminotransferase, and uric acid.

### Subgroup Analyses, Intransitivity, and Publication Bias

Because no outcome had 10 or more trials in all 3 comparisons, we did not conduct subgroup analyses.

eFigures 3 to 6 in the Supplement present the evaluation of intransitivity (a domain of indirectness) among the indirect comparisons by comparing the distribution of the potential effect modifiers across the available direct comparisons for age, study length, sample size, and percentage of males. The assumption of transitivity was met for all indirect comparisons as there was no overlap in the range between the pairwise comparisons.

eFigures 47 to 57 in the Supplement show the comparison-adjusted funnel plots for outcomes with 10 or more trial comparisons (body weight, BMI, percentage of body fat, FPI, fasting plasma glucose, low-density lipoprotein cholesterol, high-density lipoprotein cholesterol, triglycerides, total cholesterol, and systolic BP). Funnel plot asymmetry was not observed for any of the outcomes.

### GRADE Assessment 

eFigures 7 to 26 in the Supplement include the GRADE assessment for the network meta-analysis. The certainty of the evidence for body weight was moderate for LNCSBs as a substitute for SSBs (small reduction; downgrade for imprecision), moderate for water as a substitute for SSBs (no difference; downgrades for inconsistency and imprecision), and low for LNCSBs as a substitute for water (small reduction; downgrades for inconsistency and imprecision). The certainty of the evidence for the adiposity and cardiometabolic outcomes was generally moderate, ranging from very low to high for each of the 3 substitutions (downgrades for inconsistency, imprecision, and/or indirectness) and with nearly all directions of the association favoring the use of LNCSBs or water as a substitute for SSBs (small to trivial reductions) and diverging for the use of LNCSBs as a substitute for water (small to no differences).

## Discussion

In the present systematic review and meta-analysis, the use of LNCSBs as a substitute for SSBs was associated with reduced body weight, BMI, percentage of body fat, and IHCL, whereas the use of water as a substitute for SSBs was associated with no significant improvements, although the direction of association favored water in all cases. Furthermore, neither LNCSBs nor water as a substitute for SSBs was associated with significant improvements in glycemic control, BP, uric acid, or other aspects of the lipid profile or NAFLD markers, but the directions of the association favored LNCSBs or water in nearly all cases. The use of LNCSBs as a substitute for water did not show significant differences, except for a greater decrease in HbA_1c_ seen with water and in body weight and systolic BP seen with LNCSBs.

### Findings in the Context of Existing Studies

The findings in this study are in agreement with those reported in other systematic reviews and meta-analyses,^48,49,50,51^ which have allowed for the interpretation of results by comparator. Specifically, the findings that (1) reduced body weight, BMI, and body fat were associated with the use LNCSBs as a substitute for SSBs with caloric displacement and (2) neutral outcomes were associated with the use of LNCSBs as a substitute for water without caloric displacement are consistent with the results of other systematic reviews and meta-analyses of RCTs.^48,49,50,51^

Decreases in body weight,^48,49^ body weight and BMI,^50^ and a composite of body weight or BMI^51^ were observed with the substitution of LNCSs for a caloric comparator (sugars in foods or beverages) predominantly in participants with overweight or obesity. Miller and Perez^50^ further showed reductions in fat mass and waist circumference. Similarly, Toews et al^7^ found small reductions in BMI with sucrose in foods or beverages as the caloric comparator in predominantly healthy participants. On the other hand, undifferentiated analyses by Toews et al^7^ of the outcome of substituting LNCSs for a combination of caloric and noncaloric comparators and another analysis by Azad et al^8^ that restricted the outcome of substituting LNCSBs for matched noncaloric comparators (placebo, water, or weight loss diet) found no differences in body weight with LNCSs predominantly in participants with overweight or obesity. Overall, these findings are consistent with the mechanism of LNCSBs being associated with weight loss insofar as they were a factor in reducing net energy intake.

The observed improvements in downstream, intermediate cardiometabolic outcomes are also in agreement with findings of previous systematic reviews and meta-analyses. In addition to their association with weight gain,^52^ fructose-containing sugars that provide excess calories, especially in beverage form, have been associated with increased triglycerides,^53,54^ glucose,^55^ insulin,^55^ uric acid,^56^ and NAFLD markers.^57^ Toews et al^7^ showed that the use of LNCSs as a substitute for caloric sugars (sucrose) were a factor in reduced BP, and the reductions seen in IHCL would be expected through displacement of calories from SSBs.

The findings of this study can inform guidance on the role of LNCSBs in sugar-reduction strategies. There has been a particular focus on SSBs as the most important source of added or free sugars in several countries,^58,59,60^ given that the overconsumption of sugar has been associated with weight gain, diabetes, and downstream complications of hypertension and coronary heart disease.^1,2,3,4^ Although water is considered to be the standard-of-care substitution for SSBs by authoritative bodies,^5,6,15,16,17,18,19^ with many health organizations recommending against the use of LNCSBs, the existing evidence confirms the intended benefits of LNCSBs as a substitute for SSBs over the moderate term. For habitual consumers of SSBs with overweight or obesity, who are at risk for or have type 2 diabetes, and who are unable to switch to water, LNCSBs may provide a viable alternative. This finding is particularly important given that most people in the National Weight Control Registry who are successful at weight loss maintenance consume LNCSBs and report that LNCSBs help in controling caloric intake and weight loss maintenance.^61^

There is a need for high-quality RCTs that focus on quantifying the outcome of LNCSBs using different LNCS blends as substitutes for SSBs compared with the outcome of water (the standard-of-care substitution). We await the results of the STOP Sugars NOW (Strategies to Oppose Sugars With Non-nutritive Sweeteners or Water) trial and other similar RCTs to help clarify the role of LNCSBs. Future research using a range of designs is warranted to confirm whether the intended benefits of using LNCSBs as a substitute for SSBs are durable and extend to hard clinical outcomes.

### Strengths and Limitations

This systematic review and meta-analysis has several strengths. First, the use of network meta-analysis allowed for the simultaneous assessment of the 3 prespecified substitutions (LNCSBs for SSBs, water for SSBs, and LNCSBs for water), leveraging direct and indirect comparisons with a common comparator to increase the information size. Undertaking a network meta-analysis rather than a regular pairwise meta-analysis provided 2 distinct advantages: (1) more precise estimates than single direct or indirect estimates, and (2) the ability to compare interventions that had not been compared before. Second, a comprehensive literature search that included only RCTs provided the greatest protection against bias, no evidence of serious risk of bias among the included trials, and use of the GRADE approach to assess the certainty of the estimates.

This systematic review and meta-analysis also has several limitations. First, evidence of inconsistency was present in the primary outcome of body weight across the substitutions of water for SSBs and LNCSBs for water and in several secondary outcomes across the 3 prespecified substitutions, resulting in downgrades for serious inconsistency. This inconsistency was associated with either unexplained heterogeneity in the direct estimates or incoherence from the difference between direct and indirect estimates. Network estimates closely followed the direct estimate, with indirect estimates improving precision when coherent and only trivially affecting network estimates when incoherent. Second, there was evidence of serious indirectness in several of the analyses. Only 1 RCT of direct comparisons was available for several secondary outcomes, limiting generalizability and leading to downgrades for serious indirectness. The moderate median follow-up duration of 12 weeks was considered to be another potential source of indirectness across the analyses. Although there is some uncertainty about whether the benefits and lack of harm associated with LNCSBs extended beyond the 12-week median follow-up, any harm may have manifested within this time frame. The analyses also included RCTs with up to 1 year of follow-up that showed no evidence of harm or even benefit.^33,42^ Other large RCTs in children and adolescents (which were not captured in the present analyses) offer further evidence of durable benefit.^62,63^ Therefore, we did not downgrade the evidence for the lack of long-term follow-up as a source of indirectness and instead made all conclusions specific to the moderate term. Third, there was evidence of serious imprecision in several of the pooled estimates. The 95% CIs crossed the prespecified minimal important differences for the primary outcome of body weight and several secondary outcomes across the 3 prespecified substitutions. Balancing the strengths and limitations, we assessed the certainty of the evidence as generally low to moderate for most outcomes.

## Conclusions

In this systematic review and meta-analysis, using LNCSBs as an intended substitute for SSBs appeared to be associated with reductions in body weight and cardiometabolic risk factors, including BMI, percentage of body fat, and IHCL, without evidence of harm. These small improvements were similar in direction to those associated with water substitution, the standard of care. The evidence provides a good indication of the benefits of LNCSBs as an alternative replacement strategy over the moderate term for SSBs in adults with overweight or obesity who are at risk for or have diabetes.

## Notes

Supplement.
eMethods.
**eTable 1.** Search Strategy MEDLINE **eTable 2.** Search Strategy EMBASE **eTable 3.** Search Strategy Cochrane **eTable 4.** PICOTS^b^ Framework **eTable 5.** Trial Characteristics **eTable 6.** Loop-Specific Approach for Inconsistency **eTable 7.** Design by Treatment Approach for Inconsistency **eFigure 1.** Cochrane Risk of Bias Summary for All Included Trials **eFigure 2.** Risk of Bias Proportion for All Included Trials **eFigure 3.** Transitivity Analysis Box Plots Showing the Distribution of the Mean Age (Years) of the Trials Across the Available Direct Comparisons **eFigure 4.** Transitivity Analysis Box Plots Showing the Distribution of the Study Length (Weeks) of the Trials Across the Available Direct Comparisons **eFigure 5.** Transitivity Analysis Box Plots Showing the Distribution of the Sample Size of the Trials Across the Available Direct Comparisons **eFigure 6.** Transitivity Analysis Box Plots Showing the Distribution of % Males of the Trials Across the Available Direct Comparisons **eFigure 7.** Network Analysis With GRADE Assessment of the Certainty of the Evidence Comparing LNCSBs, SSBs and Water on Body Weight **eFigure 8.** Network Analysis With GRADE Assessment of the Certainty of the Evidence Comparing LNCSBs, SSBs and Water on BMI **eFigure 9.** Network Analysis With GRADE Assessment of the Certainty of the Evidence Comparing LNCSBs, SSBs and Water on Body Fat (%) **eFigure 10.** Network Analysis With GRADE Assessment of the Certainty of the Evidence Comparing LNCSBs, SSBs and Water on Waist Circumference **eFigure 11.** Network Analysis With GRADE Assessment of the Certainty of the Evidence Comparing LNCSBs, SSBs and Water on HbA1c **eFigure 12.** Network Analysis With GRADE Assessment of the Certainty of the Evidence Comparing LNCSBs, SSBs and Water on Fasting Plasma Glucose (FPG) **eFigure 13.** Network Analysis With GRADE Assessment of the Certainty of the Evidence Comparing LNCSBs, SSBs and Water on 2-hour Post-Prandial Glucose (2HPP) **eFigure 14.** Network Analysis With GRADE Assessment of the Certainty of the Evidence Comparing LNCSBs, SSBs and Water on Fasting Insulin **eFigure 15.** Network Analysis With GRADE Assessment of the Certainty of the Evidence Comparing LNCSBs, SSBs and Water on HOMA-IR **eFigure 16.** Network Analysis With GRADE Assessment of the Certainty of the Evidence Comparing LNCSBs, SSBs and Water on LDL-C **eFigure 17.** Network Analysis With GRADE Assessment of the Certainty of the Evidence Comparing LNCSBs, SSBs and Water on Non-HDL-C **eFigure 18.** Network Analysis With GRADE Assessment of the Certainty of the Evidence Comparing LNCSBs, SSBs and Water on Triglycerides **eFigure 19.** Network Analysis With GRADE Assessment of the Certainty of the Evidence Comparing LNCSBs, SSBs and Water on HDL-C **eFigure 20.** Network Analysis With GRADE Assessment of the Certainty of the Evidence Comparing LNCSBs, SSBs and Water on Total Cholesterol **eFigure 21.** Network Analysis With GRADE Assessment of the Certainty of the Evidence Comparing LNCSBs, SSBs and Water on SBP **eFigure 22.** Network Analysis With GRADE Assessment of the Certainty of the Evidence Comparing LNCSBs, SSBs and Water on DBP **eFigure 23.** Network Analysis With GRADE Assessment of the Certainty of the Evidence Comparing LNCSBs, SSBs and Water on IHCL **eFigure 24.** Network Analysis With GRADE Assessment of the Certainty of the Evidence Comparing LNCSBs, SSBs and Water on ALT **eFigure 25.** Network Analysis With GRADE Assessment of the Certainty of the Evidence Comparing LNCSBs, SSBs and Water on AST **eFigure 26.** Network Analysis With GRADE Assessment of the Certainty of the Evidence Comparing LNCSBs, SSBs and Water on Uric Acid **eFigure 27.** Network Diagram for Randomized Controlled Trials Investigating the Association of the Substitution of LNCSBs for SSBs, Water for SSBs, and LNCSBs for Water With Body Weight **eFigure 28.** Network Diagram for Randomized Controlled Trials Investigating the Association of the Substitution of LNCSBs for SSBs, Water for SSBs, and LNCSBs for Water With BMI **eFigure 29.** Network Diagram for Randomized Controlled Trials Investigating the Association of the Substitution of LNCSBs for SSBs, Water for SSBs, and LNCSBs for Water With Body Fat % **eFigure 30.** Network Diagram for Randomized Controlled Trials Investigating the Association of the Substitution of LNCSBs for SSBs, Water for SSBs, and LNCSBs for Water With Waist Circumference **eFigure 31.** Network Diagram for Randomized Controlled Trials Investigating the Association of the Substitution of LNCSBs for SSBs, Water for SSBs, and LNCSBs for Water With HbA1c **eFigure 32.** Network Diagram for Randomized Controlled Trials Investigating the Association of the Substitution of LNCSBs for SSBs, Water for SSBs, and LNCSBs for Water With FPG **eFigure 33.** Network Diagram for Randomized Controlled Trials Investigating the Association of the Substitution of LNCSBs for SSBs, Water for SSBs, and LNCSBs for Water With 2h-PP **eFigure 34.** Network Diagram for Randomized Controlled Trials Investigating the Association of the Substitution of LNCSBs for SSBs, Water for SSBs, and LNCSBs for Water With FPI **eFigure 35.** Network Diagram for Randomized Controlled Trials Investigating the Association of the Substitution of LNCSBs for SSBs, Water for SSBs, and LNCSBs for Water With HOMA-IR **eFigure 36.** Network Diagram for Randomized Controlled Trials Investigating the Association of the Substitution of LNCSBs for SSBs, Water for SSBs, and LNCSBs for Water With LDL-C **eFigure 37.** Network Diagram for Randomized Controlled Trials Investigating the Association of the Substitution of LNCSBs for SSBs, Water for SSBs, and LNCSBs for Water With Non-HDL-C **eFigure 38.** Network Diagram for Randomized Controlled Trials Investigating the Association of the Substitution of LNCSBs for SSBs, Water for SSBs, and LNCSBs for Water With TGs **eFigure 39.** Network Diagram for Randomized Controlled Trials Investigating the Association of the Substitution of LNCSBs for SSBs, Water for SSBs, and LNCSBs for Water With HDL-C **eFigure 40.** Network Diagram for Randomized Controlled Trials Investigating the Association of the Substitution of LNCSBs for SSBs, Water for SSBs, and LNCSBs for Water With TC **eFigure 41.** Network Diagram for Randomized Controlled Trials Investigating the Association of the Substitution of LNCSBs for SSBs, Water for SSBs, and LNCSBs for Water With SBP **eFigure 42.** Network Diagram for Randomized Controlled Trials Investigating the Association of the Substitution of LNCSBs for SSBs, Water for SSBs, and LNCSBs for Water With DBP **eFigure 43.** Network Diagram for Randomized Controlled Trials Investigating the Association of the Substitution of LNCSBs for SSBs, Water for SSBs, and LNCSBs for Water With IHCL **eFigure 44.** Network Diagram for Randomized Controlled Trials Investigating the Association of the Substitution of LNCSBs for SSBs, Water for SSBs, and LNCSBs for Water With ALT **eFigure 45.** Network Diagram for Randomized Controlled Trials Investigating the Association of the Substitution of LNCSBs for SSBs, Water for SSBs, and LNCSBs for Water With AST **eFigure 46.** Network Diagram for Randomized Controlled Trials Investigating the Association of the Substitution of LNCSBs for SSBs, Water for SSBs, and LNCSBs for Water With Uric Acid **eFigure 47.** Comparison Adjusted Funnel Plot for Body Weight **eFigure 48.** Comparison Adjusted Funnel Plot for BMI **eFigure 49.** Comparison Adjusted Funnel Plot for Body Fat (%) **eFigure 50.** Comparison Adjusted Funnel Plot for Fasting Insulin **eFigure 51.** Comparison Adjusted Funnel Plot for Fasting Blood Glucose **eFigure 52.** Comparison Adjusted Funnel Plot for LDL-Cholesterol **eFigure 53.** Comparison Adjusted Funnel Plot for Non-HDL-Cholesterol **eFigure 54.** Comparison Adjusted Funnel Plot for Triglycerides **eFigure 55.** Comparison Adjusted Funnel Plot for HDL-Cholesterol **eFigure 56.** Comparison Adjusted Funnel Plot for Total-Cholesterol **eFigure 57.** Comparison Adjusted Funnel Plot for Systolic Blood Pressure **eData 1.** Body Weight (Kg) **eData 2.** BMI (kg/m2) **eData 3.** Body Fat (%) **eData 4.** Waist Circumference (cm) **eData 5.** HbA1c (%) **eData 6.** Fasting Blood Glucose (mmol/L) **eData 7.** 2-Hour Post-Prandial Glucose (mmol/L) **eData 8.** Fasting Plasma Insulin (pmol/L) **eData 9.** HOMA-IR **eData 10.** LDL-Cholesterol (mmol/L) **eData 11.** Non-HDL-Cholesterol (mmol/L) **eData 12.** Triglycerides (mmol/L) **eData 13.** HDL-Cholesterol (mmol/L) **eData 14.** Total-Cholesterol (mmol/L) **eData 15.** Systolic Blood Pressure (mmHg) **eData 16.** Diastolic Blood Pressure (mmHg) **eData 17.** Intrahepatocellular Lipid/Liver Fat (SMD) **eData 18.** Alanine Aminotransferase (U/L) **eData 19.** Aspartate Aminotransferase (U/L) **eData 20.** Uric Acid (mmol/L)
