# Supplementary material for: Association of Low- and No-Calorie Sweetened Beverages as a Replacement for Sugar-Sweetened Beverages With Body Weight and Cardiometabolic Risk: A Systematic Review and Meta-analysis
Source: JAMA Netw Open. 2022 Mar 14;5(3):e222092. doi: 10.1001/jamanetworkopen.2022.2092 (PMC9907347; doi:10.1001/jamanetworkopen.2022.2092)
Supplement: Supplement. — eMethods. eTable 1. Search Strategy MEDLINE eTable 2. Search Strategy EMBASE eTable 3. Search Strategy Cochrane eTable 4. PICOTSb Framework eTable 5. Trial Characteristics eTable 6. Loop-Specific Approach for Inconsistency eTable 7. Design by Treatment Approach for Inconsistency eFigure 1. Cochrane Risk of Bias Summary for All Included Trials eFigure 2. Risk of Bias Proportion for All Included Trials eFigure 3. Transitivity Analysis Box Plots Showing the Distribution of the Mean Age (Years) of the Trials Across the Available Direct Comparisons eFigure 4. Transitivity Analysis Box Plots Showing the Distribution of the Study Length (Weeks) of the Trials Across the Available Direct Comparisons eFigure 5. Transitivity Analysis Box Plots Showing the Distribution of the Sample Size of the Trials Across the Available Direct Comparisons eFigure 6. Transitivity Analysis Box Plots Showing the Distribution of % Males of the Trials Across the Available Direct Comparisons eFigure 7. Network Analysis With GRADE Assessment of the Certainty of the Evidence Comparing LNCSBs, SSBs and Water on Body Weight eFigure 8. Network Analysis With GRADE Assessment of the Certainty of the Evidence Comparing LNCSBs, SSBs and Water on BMI eFigure 9. Network Analysis With GRADE Assessment of the Certainty of the Evidence Comparing LNCSBs, SSBs and Water on Body Fat (%) eFigure 10. Network Analysis With GRADE Assessment of the Certainty of the Evidence Comparing LNCSBs, SSBs and Water on Waist Circumference eFigure 11. Network Analysis With GRADE Assessment of the Certainty of the Evidence Comparing LNCSBs, SSBs and Water on HbA1c eFigure 12. Network Analysis With GRADE Assessment of the Certainty of the Evidence Comparing LNCSBs, SSBs and Water on Fasting Plasma Glucose (FPG) eFigure 13. Network Analysis With GRADE Assessment of the Certainty of the Evidence Comparing LNCSBs, SSBs and Water on 2-hour Post-Prandial Glucose (2HPP) eFigure 14. Network Analysis With GRADE Assessment of the Certainty o [file jamanetwopen-e222092-s001.pdf]

Supplementary Online Content

McGlynn ND, Khan TA, Wang L, et al. Association of low- and no-calorie sweetened beverages as a replacement for sugar-sweetened beverages with body weight and cardiometabolic risk: a systematic review and meta-analysis. *JAMA Netw Open*. 2022;5(3):e222092. doi:10.1001/jamanetworkopen.2022.2092

eMethods.
eTable 1. Search Strategy MEDLINE
eTable 2. Search Strategy EMBASE
eTable 3. Search Strategy Cochrane
eTable 4. PICOTS[b] Framework
eTable 5. Trial Characteristics
eTable 6. Loop-Specific Approach for Inconsistency
eTable 7. Design by Treatment Approach for Inconsistency
eFigure 1. Cochrane Risk of Bias Summary for All Included Trials
eFigure 2. Risk of Bias Proportion for All Included Trials
eFigure 3. Transitivity Analysis Box Plots Showing the Distribution of the Mean Age (Years) of the Trials Across the Available Direct Comparisons
eFigure 4. Transitivity Analysis Box Plots Showing the Distribution of the Study Length (Weeks) of the Trials Across the Available Direct Comparisons
eFigure 5. Transitivity Analysis Box Plots Showing the Distribution of the Sample Size of the Trials Across the Available Direct Comparisons
eFigure 6. Transitivity Analysis Box Plots Showing the Distribution of % Males of the Trials Across the Available Direct Comparisons
eFigure 7. Network Analysis With GRADE Assessment of the Certainty of the Evidence Comparing LNCSBs, SSBs and Water on Body Weight
eFigure 8. Network Analysis With GRADE Assessment of the Certainty of the Evidence Comparing LNCSBs, SSBs and Water on BMI
eFigure 9. Network Analysis With GRADE Assessment of the Certainty of the Evidence Comparing LNCSBs, SSBs and Water on Body Fat (%)
eFigure 10. Network Analysis With GRADE Assessment of the Certainty of the Evidence Comparing LNCSBs, SSBs and Water on Waist Circumference
eFigure 11. Network Analysis With GRADE Assessment of the Certainty of the Evidence Comparing LNCSBs, SSBs and Water on HbA1c
eFigure 12. Network Analysis With GRADE Assessment of the Certainty of the Evidence Comparing LNCSBs, SSBs and Water on Fasting Plasma Glucose (FPG)
eFigure 13. Network Analysis With GRADE Assessment of the Certainty of the Evidence Comparing LNCSBs, SSBs and Water on 2-hour Post-Prandial Glucose (2HPP)
eFigure 14. Network Analysis With GRADE Assessment of the Certainty of the Evidence Comparing LNCSBs, SSBs and Water on Fasting Insulin
eFigure 15. Network Analysis With GRADE Assessment of the Certainty of the Evidence Comparing LNCSBs, SSBs and Water on HOMA-IR

© 2022 McGlynn ND et al. *JAMA Network Open.*

eFigure 16. Network Analysis With GRADE Assessment of the Certainty of the Evidence Comparing LNCSBs, SSBs and Water on LDL-C
eFigure 17. Network Analysis With GRADE Assessment of the Certainty of the Evidence Comparing LNCSBs, SSBs and Water on Non-HDL-C
eFigure 18. Network Analysis With GRADE Assessment of the Certainty of the Evidence Comparing LNCSBs, SSBs and Water on Triglycerides
eFigure 19. Network Analysis With GRADE Assessment of the Certainty of the Evidence Comparing LNCSBs, SSBs and Water on HDL-C
eFigure 20. Network Analysis With GRADE Assessment of the Certainty of the Evidence Comparing LNCSBs, SSBs and Water on Total Cholesterol
eFigure 21. Network Analysis With GRADE Assessment of the Certainty of the Evidence Comparing LNCSBs, SSBs and Water on SBP
eFigure 22. Network Analysis With GRADE Assessment of the Certainty of the Evidence Comparing LNCSBs, SSBs and Water on DBP
eFigure 23. Network Analysis With GRADE Assessment of the Certainty of the Evidence Comparing LNCSBs, SSBs and Water on IHCL
eFigure 24. Network Analysis With GRADE Assessment of the Certainty of the Evidence Comparing LNCSBs, SSBs and Water on ALT
eFigure 25. Network Analysis With GRADE Assessment of the Certainty of the Evidence Comparing LNCSBs, SSBs and Water on AST
eFigure 26. Network Analysis With GRADE Assessment of the Certainty of the Evidence Comparing LNCSBs, SSBs and Water on Uric Acid
eFigure 27. Network Diagram for Randomized Controlled Trials Investigating the Association of the Substitution of LNCSBs for SSBs, Water for SSBs, and LNCSBs for Water With Body Weight
eFigure 28. Network Diagram for Randomized Controlled Trials Investigating the Association of the Substitution of LNCSBs for SSBs, Water for SSBs, and LNCSBs for Water With BMI
eFigure 29. Network Diagram for Randomized Controlled Trials Investigating the Association of the Substitution of LNCSBs for SSBs, Water for SSBs, and LNCSBs for Water With Body Fat %
eFigure 30. Network Diagram for Randomized Controlled Trials Investigating the Association of the Substitution of LNCSBs for SSBs, Water for SSBs, and LNCSBs for Water With Waist Circumference
eFigure 31. Network Diagram for Randomized Controlled Trials Investigating the Association of the Substitution of LNCSBs for SSBs, Water for SSBs, and LNCSBs for Water With HbA1c
eFigure 32. Network Diagram for Randomized Controlled Trials Investigating the Association of the Substitution of LNCSBs for SSBs, Water for SSBs, and LNCSBs for Water With FPG
eFigure 33. Network Diagram for Randomized Controlled Trials Investigating the Association of the Substitution of LNCSBs for SSBs, Water for SSBs, and LNCSBs for Water With 2h-PP
eFigure 34. Network Diagram for Randomized Controlled Trials Investigating the Association of the Substitution of LNCSBs for SSBs, Water for SSBs, and LNCSBs for Water With FPI
eFigure 35. Network Diagram for Randomized Controlled Trials Investigating the Association of the Substitution of LNCSBs for SSBs, Water for SSBs, and LNCSBs for Water With HOMA-IR
eFigure 36. Network Diagram for Randomized Controlled Trials Investigating the Association of the Substitution of LNCSBs for SSBs, Water for SSBs, and LNCSBs for Water With LDL-C

© 2022 McGlynn ND et al. *JAMA Network Open.*

eFigure 37. Network Diagram for Randomized Controlled Trials Investigating the Association of the Substitution of LNCSBs for SSBs, Water for SSBs, and LNCSBs for Water With Non-HDL-C
eFigure 38. Network Diagram for Randomized Controlled Trials Investigating the Association of the Substitution of LNCSBs for SSBs, Water for SSBs, and LNCSBs for Water With TGs
eFigure 39. Network Diagram for Randomized Controlled Trials Investigating the Association of the Substitution of LNCSBs for SSBs, Water for SSBs, and LNCSBs for Water With HDL-C
eFigure 40. Network Diagram for Randomized Controlled Trials Investigating the Association of the Substitution of LNCSBs for SSBs, Water for SSBs, and LNCSBs for Water With TC
eFigure 41. Network Diagram for Randomized Controlled Trials Investigating the Association of the Substitution of LNCSBs for SSBs, Water for SSBs, and LNCSBs for Water With SBP
eFigure 42. Network Diagram for Randomized Controlled Trials Investigating the Association of the Substitution of LNCSBs for SSBs, Water for SSBs, and LNCSBs for Water With DBP
eFigure 43. Network Diagram for Randomized Controlled Trials Investigating the Association of the Substitution of LNCSBs for SSBs, Water for SSBs, and LNCSBs for Water With IHCL
eFigure 44. Network Diagram for Randomized Controlled Trials Investigating the Association of the Substitution of LNCSBs for SSBs, Water for SSBs, and LNCSBs for Water With ALT
eFigure 45. Network Diagram for Randomized Controlled Trials Investigating the Association of the Substitution of LNCSBs for SSBs, Water for SSBs, and LNCSBs for Water With AST
eFigure 46. Network Diagram for Randomized Controlled Trials Investigating the Association of the Substitution of LNCSBs for SSBs, Water for SSBs, and LNCSBs for Water With Uric Acid
eFigure 47. Comparison Adjusted Funnel Plot for Body Weight
eFigure 48. Comparison Adjusted Funnel Plot for BMI
eFigure 49. Comparison Adjusted Funnel Plot for Body Fat (%)
eFigure 50. Comparison Adjusted Funnel Plot for Fasting Insulin
eFigure 51. Comparison Adjusted Funnel Plot for Fasting Blood Glucose
eFigure 52. Comparison Adjusted Funnel Plot for LDL-Cholesterol
eFigure 53. Comparison Adjusted Funnel Plot for Non-HDL-Cholesterol
eFigure 54. Comparison Adjusted Funnel Plot for Triglycerides
eFigure 55. Comparison Adjusted Funnel Plot for HDL-Cholesterol
eFigure 56. Comparison Adjusted Funnel Plot for Total-Cholesterol
eFigure 57. Comparison Adjusted Funnel Plot for Systolic Blood Pressure
eData 1. Body Weight (Kg)
eData 2. BMI (kg/m2)
eData 3. Body Fat (%)
eData 4. Waist Circumference (cm)
eData 5. HbA1c (%)
eData 6. Fasting Blood Glucose (mmol/L)
eData 7. 2-Hour Post-Prandial Glucose (mmol/L)
eData 8. Fasting Plasma Insulin (pmol/L)
eData 9. HOMA-IR
eData 10. LDL-Cholesterol (mmol/L)
eData 11. Non-HDL-Cholesterol (mmol/L)
eData 12. Triglycerides (mmol/L)
eData 13. HDL-Cholesterol (mmol/L)

© 2022 McGlynn ND et al. *JAMA Network Open.*

eData 14. Total-Cholesterol (mmol/L)
eData 15. Systolic Blood Pressure (mmHg)
eData 16. Diastolic Blood Pressure (mmHg)
eData 17. Intrahepatocellular Lipid/Liver Fat (SMD)
eData 18. Alanine Aminotransferase (U/L)
eData 19. Aspartate Aminotransferase (U/L)
eData 20. Uric Acid (mmol/L)

This supplementary material has been provided by the authors to give readers additional information about their work.

© 2022 McGlynn ND et al. *JAMA Network Open.*

eMethods

Data sources and searches

We searched MEDLINE, Embase, and the Cochrane Library from inception through 26 December 2021. The full search strategy is presented in online eTable S2-4. Briefly, we searched using variations of the exposure terms (low and non-nutritive sweetened beverages (LNCSBs), sugar-sweetened beverages (SSBs)), outcome terms (adiposity, glycemia, blood lipids, blood pressure, non-alcoholic fatty liver disease (NAFLD) and uric acid) and study design terms (randomized controlled trial, randomized, placebo). The search was limited to human studies with no language restriction. Additionally, reference lists of selected studies and reviews were searched, experts in the field were contacted and Google Scholar searches were conducted to identify any additional articles.

Study selection

Online eTables S5 shows the summary of our study selection using the PICOTS framework [1]. We included RCTs of at least 2-weeks duration that investigated the effect of one of three beverages (LNCSBs, SSBs or water) on cardiometabolic risk factors compared to another among the three beverages. Trials were included if the intervention arm assessed the effect of LNCSBs, SSBs or water consumed alone or while on a weight-loss or nutrition education program in adults. Trials were excluded if they had non-usual intake method (e.g., used low and non-nutritive sweeteners (LNCS) capsules that bypass the oral taste receptors); had a mixed intervention; had a duration of <2 weeks; did not use a comparator arm of LNCSBs, SSBs or water; included children, pregnant or breastfeeding women; or did not provide suitable end-point data. We only studied LNCS in liquid (beverage trials) as these allow for clear comparisons. Studies of LNCSs added to foods, fortified beverages or nutrient-dense beverages (e.g., milk, juice, etc.) were excluded due to the presence of other macronutrients. When multiple publications existed for the same study, the article with the most updated information was included. Published abstracts were not included.

Data extraction

Two independent reviewers (NM and RZ) assessed the titles and abstracts of all identified studies and reviewed and extracted relevant data from each report, including study design, blinding, sample size, participant characteristics, follow-up duration, nature of the interventions/comparators, doses, outcome data and funding source. In those trials where the data were only reported in figures, we extracted data using Plot Digitizer V.2.6.8 (http://plotdigitizer.sourceforge.net/). Additional information was requested from the authors when necessary. Disagreement were resolved by consensus or, when necessary, arbitration by TAK and JLS.

© 2022 McGlynn ND et al. JAMA Network Open.

Risk of bias assessment

Two independent reviewers (NM and RZ) assessed Risk of bias for each included trial using the Cochrane Risk of Bias tool [2]. Assessment was done across 5 domains of bias (sequence generation, allocation concealment, blinding of participants and personnel, incomplete outcome data and selective reporting). The risk of bias was assessed as either low (proper methods taken to reduce bias), high (improper methods creating bias) or unclear (insufficient information provided to determine the bias level).

Data synthesis

Mean differences between the arms and their respective variance terms were extracted and used as the basis for analysis for each trial comparison. If mean differences were not provided, they were derived from available data using published formulas.[3] When median data were reported, they were converted to mean data with corresponding variances using established methods.[4,5] When no variance data were available, the standard deviation was taken from a trial similar in size, participants, and nature of intervention. We used change from baseline values from each study to calculate the mean differences between treatments for each substitution (LNCSBs for SSBs, water for SSBs and LNCSBs for water), otherwise we used post-intervention values (Supplementary Data S1-S20). For crossover trials and for within arm changes in parallel trials, we used a correlation coefficient of 0.5 in pairwise analysis to calculate standard errors.[6-8] To mitigate a unit of analysis error, when arms of trials with multiple interventions or control arms were used more than once, the corresponding sample size was divided accordingly.[3] Non-HDL-C values that were not reported were derived by subtracting HDL-C from total cholesterol values with standard errors derived from HDL-C and total cholesterol variance data using the inverse variance law.[9] For trials in which the change in BMI was not reported, but body weight was reported, then if baseline BMI was available, these data were used to calculate the height, which could then be used to calculate the end BMI and change in BMI. The change in BMI variance was imputed using published formula[3] and a correlation coefficient of 0.5.[6-8]

Network meta-analysis, based on a frequentist framework, was conducted using the "network" suite of commands available in STATA version 15 (College Station, TX: StataCorp LP). We performed a random-effects network meta-analysis for each outcome to compare the three interventions simultaneously (LNCSBs, SSBs and water) in a single analysis by combining both direct and indirect evidence across the selected network of studies. The network meta-analysis synthesized all of the available evidence (direct and indirect effects) and quantified the pooled network effect of each intervention against every other intervention. We reported our results as mean differences (MDs) and 95% confidence intervals (CIs). To display the results for outcomes on the same plot, standardized mean differences (SMDs) and pseudo

© 2022 McGlynn ND et al. JAMA Network Open.

95% CIs were calculated, whereby the SMD 95% CIs were proportionally scaled to the MD 95% CIs. The network diagrams were generated to show the interactions among the studies included in the network meta-analysis and to illustrate the available direct comparisons between treatments [10].

Inconsistency was assessed in the direct, indirect, and network estimates. We assessed interstudy heterogeneity in the direct (each pairwise comparison arm) estimates using the Cochran Q statistic with quantification by the I^2 statistic, where an $I^2 \geq 50\%$, $P<0.10$ was considered an indication of substantial interstudy heterogeneity. We measured incoherence in the network estimates using both local (loop-specific and side-splitting) and global (design-by-treatment interaction model) approaches to evaluate the presence of incoherence. The loop-specific approach looked at the inconsistency in each closed loop in the network [10] while the side-splitting approach detected direct estimate comparisons that disagreed with the indirect evidence from the entire network [11]. The design-by-treatment interaction model was applied as a global approach to check simultaneously for inconsistency from all possible sources in the network [12]. If ≥10 comparisons were available for all comparisons, then we conducted a priori subgroup analyses by age, study duration, type of design, disease status, risk of bias and funding source.

Indirectness was assessed in the indirect comparisons by evaluation of intransitivity across the pairwise comparisons comprising the indirect estimates for important study characteristics including age, study length, sample size and percentage of males. Intransitivity was considered present if there was no overlap in the range between the pairwise comparisons.

Publication bias was assessed if ≥10 trial comparisons were available for all comparisons, We used comparison-adjusted funnel plots to assess funnel plot asymmetry[10]. Asymmetry around the line of the meta-analysis summary effect suggested evidence of small-study effects.

Grading of the evidence

We assessed the certainty of the evidence using the Grading of Recommendations Assessment, Development, and Evaluation (GRADE) system [13] with an extension for network meta-analyses [14] and other recent guidance [15-17] from the GRADE Working group. Evidence was graded as high, moderate, low or very low certainty. Network estimates of RCTs and the direct and indirect estimates that make-up these network estimates started at high certainty of evidence and were downgraded by established criteria. These criteria included serious risk of bias (weight of RCTs in the direct estimates show high risk of bias assessed by the Cochrane Risk of Bias tool), inconsistency (substantial unexplained heterogeneity [$I^2 \geq 50\%$, $P_Q<0.10$] in the direct estimates and incoherence in the network estimates by the loop-specific [p<0.05], side-splitting [p<0.05] and design-by-treatment interaction model, p<0.05 or if by visual inspection the direct

© 2022 McGlynn ND et al. JAMA Network Open.

and indirect results were in opposite directions), indirectness (presence of factors related to the participants, interventions, and study conditions that limited the generalizability of the results among the direct estimates and intransitivity in the indirect estimates or if information for the network estimate was based upon only 1 direct study or only from indirect studies), imprecision (the 95% CIs for effect estimates were wide and crossed prespecified minimally important differences (MIDs) for benefit and/or harm in the direct and network estimates) and publication bias (evidence of comparison adjusted funnel plot asymmetry).

© 2022 McGlynn ND et al. JAMA Network Open.

eTables

eTable 1. Search strategy MEDLINE

Database	MEDLINE[1]

Search Terms

1. aspartame.mp.
2. exp Aspartame/
3. neotame.mp.
4. saccharin.mp.
5. exp Saccharin/
6. sucralose.mp.
7. stevia.mp.
8. exp Stevia/
9. acesulfame.mp.
10. exp Sweetening Agents/
11. sugar substitute*.mp.
12. noncaloric.mp.
13. non-caloric.mp.
14. nonnutritive.mp.
15. non-nutritive.mp.
16. no calorie*.mp.
17. low calorie sweeten*.mp.
18. sugar-free.mp.
19. artificial sweet*.mp.
20. diet beverage*.mp.
21. diet soda.mp.
22. exp Carbonated Beverages/
23. ssb.mp.
24. sugar*sweetened beverage*.mp.
25. Fructose/
26. fructose.mp.
27. dietary sucrose/
28. sucrose.mp.
29. High Fructose Corn Syrup/
30. High Fructose Corn Syrup.mp.
31. Soft drink*.mp.
32. cola.mp.
33. sugar*.mp.
34. or/1-33

35. Blood Pressure/
36. systolic blood pressure.mp.
37. SBP.mp.
38. diastolic blood pressure.mp.
39. DBP.mp.
40. 35 or 36 or 37 or 38 or 39

41. glyc*m*.mp.
42. Hemoglobin A, Glycosylated/
43. glyc*mia.mp.
44. insulin*.mp.
45. gly*albumin.mp.
46. OGTT.mp.
47. hba1c.mp.
48. HOMA*.mp.
49. fructosamine*.mp.
50. Insulin/
51. exp Glucose/
52. Glucose Tolerance Test/
53. or/41-52
54. triglyceride.mp.
55. triacylglycerol.mp.
56. VLDL.mp.
57. very low density lipoprotein.mp.
58. lipid*.mp.
59. lipids/
60. cholesterol/
61. cholesterol.mp.
62. lipoprotein.mp.
63. lipoproteins/
64. (hdl or high density lipoprotein).mp.
65. (ldl or low density lipoprotein).mp.
66. exp hyperlipidemias/
67. apolipoprotein*.mp.
68. non-HDL.mp.
69. or/54-68

70. fatty liver.mp.
71. non-alcoholic fatty liver disease/
72. NAFLD.mp.
73. transaminases/
74. alanine transaminase/
75. alt.mp.
76. aspartate aminotransferase/
77. ast.mp.
78. IHCL.mp.
79. Intrahepatocellular lipid.mp.
80. transamin*.mp.
81. or/70-80

82. exp uric acid/
83. uric acid.mp.
84. urate.mp.
85. hyperuricemia/
86. hyperuricemia.mp.
87. hyperuricaemia.mp.
88. uric.mp.
89. or/82-88

90. “body weight”.mp.
91. Exp Body Weight/
92. Exp Weight Gain/
93. or/90-92
94. 40 or 53 or 69 or 81 or 89
95. 34 and 94
96. limit 95 to animals
97. 95 not 96
98. review.pt.
99. (medline or medlars or emabse or pubmed or Cochrane)
100. (scisearch or psychinfo or psychinfo).tw,sh.
101. (psychlit or psyclit).tw,sh.
102. cinahl.tw,sh.
103. ((hand adj2 search$) or (manual$ adj2 search$)).tw,sh.
104. (electronic database$ or bibliographic database$ or computeri?ed database$ or online database$).tw,sh.
105. (pooling or pooled or mantel haenszel).tw,sh.
106. (peto or dersimonian or der simonian or fixed effect).tw,sh.
107. (retraction of publication or retracted publication).pt.
108. or/99-106
109. 98 and 108
110. meta-analysis.pt.
111. meta-analysis.sh.
112. (meta-analys$ or meta analys$ or metaanalys$).tw,sh.
113. (systematic$ adj5 review$).tw,sh.
114. (systematic$ adj5 overview$).tw,sh.
115. (quantitativ$ adj5 review$).tw,sh.
116. (quantitativ$ adj5 overview$).tw,sh.
117. (quantitativ$ adj5 synthesis$).tw,sh.
118. (methodologic$ adj5 review$).tw,sh.
119. (methodologic$ adj5 overview$).tw,sh.
120. (integrative research review$ or research integration).tw.
121. or/110-119
122. 109 or 121
123. exp cohort studies/
124. cohort$.tw.
125. controlled clinical trial.pt.
126. epidemiologic methods/
127. limit 126 to yr=1966-1989
128. exp case-control studies/
129. (case$ and control$).tw.
130. (case$ and series).tw.
131. case reports.pt.
132. (case$ adj2 report$).tw.
133. (case$ adj2 stud$).tw.
134. or/123-125,127-132
135. 122 or 134
136. 97 not 135
137. randomized controlled trial.pt.
138. (random$ or placebo$ or single blind$ or double blind$ or triple blind$).ti,ab.
139. (retraction of publication or retracted publication).pt.
140. or/137-139
141. (animals not humans).sh.
142. ((comment or editorial or meta-analysis or practice-guideline or review or letter) not randomized controlled trial).pt.
143. (random sampl$ or random digit$ or random effect$ or random survey or random regression).ti,ab. not randomized controlled trial.pt.
144. or/141-143
145. 140 not 144
146. 136 and 145
147. 97 and 145
148. from 147 keep 1-3511

[1] For all databases, the original search date was 28 March 2019, updated 31 March 2021 and then finally on 26 March 2021

© 2022 McGlynn ND et al. JAMA Network Open.

eTable 2. Search strategy EMBASE

Database	EMBASE, Cochrane[1]
Search Terms	1. aspartame.mp.<br>2. exp Aspartame/<br>3. neotame.mp.<br>4. saccharin.mp.<br>5. exp Saccharin/<br>6. sucralose.mp.<br>7. stevia.mp.<br>8. exp Stevia/<br>9. acesulfame.mp.<br>10. Sweetening Agents/<br>11. sugar substitute*.mp.<br>12. noncaloric.mp.<br>13. non-caloric.mp.<br>14. nonnutritive.mp.<br>15. non-nutritive.mp.<br>16. no calorie*.mp.<br>17. low calorie sweeten*.mp.<br>18. sugar-free.mp.<br>19. artificial sweet*.mp.<br>20. diet beverage*.mp.<br>21. diet soda.mp.<br>22. exp Carbonated Beverages/<br>23. ssb.mp.<br>24. sugar*sweetened beverage*.mp.<br>25. Fructose/<br>26. fructose.mp.<br>27. dietary sucrose/<br>28. sucrose.mp.<br>29. High Fructose Corn Syrup/<br>30. High Fructose Corn Syrup.mp.<br>31. Soft drink*.mp.<br>32. cola.mp.<br>33. sugar*.mp.<br>34. or/1-33<br><br>35. Blood Pressure/<br>36. systolic blood pressure.mp.<br>37. SBP.mp.<br>38. diastolic blood pressure.mp.<br>39. DBP.mp.<br>40. or/35-39<br><br>41. glyc*m*.mp.<br>42. Hemoglobin A, Glycosylated/<br>43. glyc*mia.mp.<br>44. insulin*.mp.<br>45. gly*albumin.mp.<br>46. OGTT.mp.<br>47. hba1c.mp.<br>48. HOMA*.mp.<br>49. fructosamine*.mp.<br>50. Insulin/<br>51. exp Glucose/<br>52. Glucose Tolerance Test/<br>53. or/41-52<br>54. triglyceride.mp.<br>55. triacylglycerol.mp.<br>56. VLDL.mp.<br>57. very low density lipoprotein.mp.<br>58. lipid*.mp.<br>59. lipids/<br>60. cholesterol/<br>61. cholesterol.mp.<br>62. lipoprotein.mp.<br>63. lipoproteins/<br>64. (hdl or high density lipoprotein).mp.<br>65. (ldl or low density lipoprotein).mp.<br>66. exp hyperlipidemias/<br>67. apolipoprotein*.mp.<br>68. non-HDL.mp.<br>69. or/54-68<br><br>70. fatty liver.mp.<br>71. non-alcoholic fatty liver disease/<br>72. NAFLD.mp.<br>73. transaminases/<br>74. alanine transaminase/<br>75. alt.mp.<br>76. aspartate aminotransferase/<br>77. ast.mp.<br>78. IHCL.mp.<br>79. Intrahepatocellular lipid.mp.<br>80. transamin*.mp.<br>81. or/70-80<br><br>82. exp uric acid/<br>83. uric acid.mp.<br>84. urate.mp.<br>85. hyperuricemia/<br>86. hyperuricemia.mp.<br>87. hyperuricaemia.mp.<br>88. uric.mp.<br>89. or/82-88<br><br>90. “body weight”.mp.<br>91. Exp Body Weight/<br>92. Exp Weight Gain/<br>93. or/90-92<br>94. 40 or 53 or 69 or 81 or 89 or 93<br>95. 34 and 94<br>96. limit 95 to animals<br>97. 95 not 96<br>98. exp review/<br>99. (literature adj3 review$).ti,ab.<br>100. exp meta analysis/<br>101. exp Systematic Review/<br>102. or/98-101<br>103. (medline or medlars or embase or pubmed or cinahl or amed or psychlit or psyclit or psychinfo or psycinfo or scisearch or cochrane).ti,ab.<br>104. RETRACTED ARTICLE/<br>105. 103 or 104<br>106. 102 or 105<br>107. (systematic$ adj2 (review$ or overview)).ti,ab.<br>108. o(meta?anal$ or meta anal$ or meta-anal$ or metaanal$ or metanal$).ti,ab.<br>109. or/106-108<br>110. exp cohort analysis/<br>111. exp longitudinal study/<br>112. exp prospective study/<br>113. exp follow up/<br>114. cohort$.tw.<br>115. exp case control study/<br>116. (case$ and control$).tw.<br>117. exp case study/<br>118. (case$ and series).tw.<br>119. case report/<br>120. (case$ adj2 report$).tw.<br>121. (case$ adj2 stud$).tw.<br>122. or/110-121<br>123. 106 or 122<br>124. 97 not 123<br>125. (random$ or placebo$ or single blind$ or double blind$ or triple blind$).ti,ab.<br>126. RETRACTED ARTICLE/<br>127. 125 or 126<br>128. (animal$ not human$).sh,hw.<br>129. (book or conference paper or editorial or letter or review).pt. not exp randomized controlled trial/<br>130. (random sampl$ or random digit$ or random effect$ or random survey or random regression).ti,ab. not exp randomized controlled trial/<br>131. or/128-130<br>132. 127 not 131<br>133. 124 and 132

[1] For all databases, the original search date was 28 March 2019, updated 31 March 2021 and then finally on 26 March 2021

© 2022 McGlynn ND et al. JAMA Network Open.

eTable 3. Search strategy Cochrane

Database	Cochrane[1]	
Search Terms	1. aspartame.mp.<br>2. exp Aspartame/<br>3. neotame.mp.<br>4. saccharin.mp.<br>5. exp Saccharin/<br>6. sucralose.mp.<br>7. stevia.mp.<br>8. exp Stevia/<br>9. acesulfame.mp.<br>10. Sweetening Agents/<br>11. sugar substitute*.mp.<br>12. noncaloric.mp.<br>13. non-caloric.mp.<br>14. nonnutritive.mp.<br>15. non-nutritive.mp.<br>16. low calorie sweeten*.mp.<br>17. sugar-free.mp.<br>18. artificial sweet*.mp.<br>19. diet beverage*.mp.<br>20. diet soda.mp.<br>21. exp Carbonated Beverages/<br>22. ssb.mp.<br>23. sugar*sweetened beverage*.mp.<br>24. Fructose/<br>25. fructose.mp.<br>26. dietary sucrose/<br>27. sucrose.mp.<br>28. High Fructose Corn Syrup/<br>29. High Fructose Corn Syrup.mp.<br>30. Soft drink*.mp.<br>31. cola.mp.<br>32. sugar*.mp.<br>33. or/1-32<br><br>34. Blood Pressure/<br>35. systolic blood pressure.mp.<br>36. SBP.mp.<br>37. diastolic blood pressure.mp.<br>38. DBP.mp.<br>39. Or/35-39<br><br>40. glyc*m*.mp.<br>41. Hemoglobin A, Glycosylated/<br>42. glyc*mia.mp.<br>43. insulin*.mp.<br>44. gly*albumin.mp.<br>45. OGTT.mp.<br>46. hba1c.mp.<br>47. HOMA*.mp.<br>48. fructosamine*.mp.<br>49. Insulin/<br>50. exp Glucose/<br>51. Glucose Tolerance Test/<br>52. or/41-52	53. triglyceride.mp.<br>54. triacylglycerol.mp.<br>55. VLDL.mp.<br>56. very low density lipoprotein.mp.<br>57. lipid*.mp.<br>58. lipids/<br>59. cholesterol/<br>60. cholesterol.mp.<br>61. lipoprotein.mp.<br>62. lipoproteins/<br>63. (hdl or high density lipoprotein).mp.<br>64. (ldl or low density lipoprotein).mp.<br>65. exp hyperlipidemias/<br>66. apolipoprotein*.mp.<br>67. non-HDL.mp.<br>68. or/54-68<br><br>69. fatty liver.mp.<br>70. non-alcoholic fatty liver disease/<br>71. NAFLD.mp.<br>72. transaminases/<br>73. alanine transaminase/<br>74. alt.mp.<br>75. aspartate aminotransferase/<br>76. ast.mp.<br>77. IHCL.mp.<br>78. Intrahepatocellular lipid.mp.<br>79. transamin*.mp.<br>80. or/70-80<br><br>81. exp uric acid/<br>82. uric acid.mp.<br>83. urate.mp.<br>84. hyperuricemia/<br>85. hyperuricemia.mp.<br>86. hyperuricaemia.mp.<br>87. uric.mp.<br>88. or/82-88<br><br>89. “body weight”.mp.<br>90. exp Body Weight/<br>91. exp Weight Gain/<br>92. or/90-92<br>93. 39 or 52 or 68 or 80 or 88 or 92<br>94. 33 and 93<br>95. limit 95 to randomized controlled trial

[1] For all databases, the original search date was 28 March 2019, updated 31 March 2021 and then finally on 26 March 2021

© 2022 McGlynn ND et al. JAMA Network Open.

eTable 4. PICOTS[b] framework

Participants	Intervention	Comparison	Outcome	Time	Study Design
Adult men and women excluding pregnant or breastfeeding women and children. All health backgrounds	LNCSBs or SSBs or water	LNCSBs or SSBs or water	Adiposity, glycemic control, established blood lipid targets, blood pressure, non-alcoholic fatty liver and uric acid	≥ 2 weeks	Human randomized controlled trials

[b]Hutton B, Salanti G, Caldwell DM, Chaimani A, Schmid CH, Cameron C, Ioannidis JP, Straus S, Thorlund K, Jansen JP, Mulrow C, Catalá-López F, Gøtzsche PC, Dickersin K, Boutron I, Altman DG, Moher D. The PRISMA extension statement for reporting of systematic reviews incorporating network meta-analyses of health care interventions: checklist and explanations. Ann Intern Med. 2015 Jun 2;162(11):777-84.

© 2022 McGlynn ND et al. JAMA Network Open.

eTable 5. Trial characteristics

Study	Subjects	Age<br>Mean (SD)	Sex No. (%)<br>Males:Females	LNCS<br>Type	Beverage Dose (mL/d)			Setting	Design	Duration<br>(weeks)	Funding<br>Source	Contacted for data / Provided data
					LNCSB	Water	SSB					
Bonnet et al. 2018<br>LNCSB:Water	50<br>(22M:28F)	31.1 (10.3)	22:28<br>(44:56)	Asp/ Ace-K	660	660	NA	France	C	12	Agency/ Industry	Yes / No
*Bruun et al. 2015<br>LNCSB<br>Water<br>SSB	35<br>(3M:9F)<br>(5M:8F)<br>(6M:4F)	39 (1.1)<br>39 (27.7)<br>39 (28.8)<br>39 (19)	14:21<br>(40:60)	Asp	1000	1000	1000	Denmark	P	26	Agency/ Industry	Yes / No
Ebbeling et al. 2020<br>LNCSB<br>Water<br>SSB	 67 (40M, 27F)<br>69 (41M, 28F)<br>67 (40M, 27F)	26.8(5.6)<br>26.7(5.7)<br>27.9(6.0)<br>25.9(5.1)	121:82<br>(60:40)	NR	355	355	355	USA	P	52	Agency	
Campos et al. 2015<br>LNCSB<br>SSB	27<br>(8M:6F)<br>(6M:7F)	NR	14:13<br>(52:48)	NR	1300	NA	1300	Switzerland	P	12	Agency	Yes / Yes
*Engel et al. 2017<br>LNCSB<br>Water<br>SSB	45<br>(3M:12F)<br>(5M:11F)<br>(8M:6F)	38.6 (7.6)<br>39 (7.6)<br>39 (7.3)<br>37.8 (8)	16:29<br>(36:64)	NR	1000	1000	1000	Denmark	P	26	Agency/ Industry	Yes / No
Hernández-Cordero et al. 2014<br>Water<br>SSB	240<br>120F<br>120F	33.3 (6.7)<br>33.5 (6.7)<br>33.3 (6.7)	0:240<br>(0:100)	NR	NA	250+	250+	Mexico	P	39	Industry	
Higgins et al. 2018<br>Water<br>350mg LNCSB<br>1050mg LNCSB	93<br>(13M:18F)<br>(17M:14F)<br>(13M:18F)	22.9 (1.0)<br>24.2 (1.3)<br>22.8 (1.0)<br>21.8 (0.6)	43:50<br>(46:54)	Asp	500	500	NA	USA	P	12	Industry	
Higgins & Mattes 2019<br>LNCSB (Sac)<br>LNCSB (Asp)<br>LNCSB (RebA)<br>LNCSB (Suc)<br>SSB	154<br>(12M:17F)<br>(15M:15F)<br>(10M:18F)<br>(12M:16F)<br>(18M:21F)	27.3 (9.6)<br>25.8 (6.9)<br>29.5 (12)<br>27.1 (9.6)<br>25.9 (9)<br>28.2 (9.5)	67:87<br>(44:56)	Sac,<br>Asp,<br>RebA,<br>Suc	1250-1750	NA	1250-1750	USA	P	12	Agency	Yes / Yes
Madjd et al. 2015<br>LNCSB<br>Water	62<br>32F<br>30F	32 (6.9)<br>31.7 (6.8)<br>32.2 (6.9)	0:62<br>(0:100)	NR	250+	250+	NA	Iran	P	24	Agency	
Madjd et al. 2017<br>LNCSB<br>Water	81<br>40F<br>41F	34.8 (7.2)<br>35.5 (7.5)<br>34.2 (7)	0:81<br>(0:100)	NR	250+	250+	NA	Iran	P	24	Agency	
Maersk et al. 2012<br>LNCSB<br>Water<br>SSB	35<br>(3M:9F)<br>(5M:8F)<br>(6M:4F)	39 (26)<br>39(27.7)<br>39(28.8)<br>39(19)	14:21<br>(40:60)	NR	1000	1000	1000	Denmark	P	26	Agency/ Industry	
Peters et al. 2016<br>LNCSB<br>Water	308<br>(28M:130F)<br>(25M:125F)	47.8 (10.5)<br>48.3 (10.4)<br>47.3 (10.6)	53:255<br>(17:83)	NR	710	710	NA	USA	P	52	Industry	
Reid et al. 2007<br>LNCSB<br>SSB	133<br>65F<br>68F	31.8 (9.1)<br>NR<br>NR	0:133<br>(0:100)	Asp	1000	NA	1000	England	P	4	Agency	
Reid et al. 2010<br>LNCSB<br>SSB	53<br>29F<br>24F	33.7 (9.9)<br>32.9 (8.8)<br>34.5 (11)	0:53<br>(0:100)	Asp	1000	NA	1000	Scotland	P	4	Agency	Yes / No
Reid et al. 2014<br>LNCSB<br>SSB	41<br>21F<br>20F	35 (9.1)<br>34.6 (8.5)<br>35.1 (9.9)	0:41<br>(0:100)	Asp	1000	NA	1000	Scotland	P	4	Agency/ Industry	Yes / No
Tate et al. 2012<br>LNCSB<br>Water	213<br>(23M:82F)<br>(12M:96F)	42 (10.7)<br>41.2 (11.2)<br>43.2 (10.6)	35:178<br>(52:48)	NR	1420-2000	1420- 2000	NA	USA	P	26	Industry	
Tordoff et al. 1990<br>LNCSB: SSB	30<br>(21M:9F)	25.6 (5.3)	21:9<br>(70:30)	Asp	1135	NA	1135	USA	C	3	Agency	

Asp, aspartame; Ace-K, acesulfame potassium; NA, not applicable; No., number; LNCS, low and non-nutritive sweetener; LNCSB, low and non-nutritive sweetened beverage; NW, normal weight; NR, not reported; OB, obese; OW, overweight; RebA, rebaudioside A; Sac, saccharin; SD, standard deviation; Suc, sucralose; SSB, sugar-sweetened beverage; T2DM, type 2 diabetes mellitus.
*Secondary analyses to Maersk et al. 2012. As more outcomes were reported in the Engel et al. 2017 analysis, data from that trial was used for the majority of outcomes, except for uric acid (Bruun et al.) and IHCL (Maersk et al).

© 2022 McGlynn ND et al. JAMA Network Open.

eTable 6. Loop-specific approach for inconsistency

Outcome	P-value	Inconsistency
Body weight	P=0.074	Not present
BMI	P=0.8	Not present
Body fat	P=0.98	Not present
Waist circumference	Unable to calculate*	
HbA1c	Unable to calculate*	
FPG	P=0.25	Not present
2h-PG	No triangular loop found	
FPI	P=0.75	Not present
HOMA-IR	P=0.76	Not present
LDL-C	P=0.95	Not present
Non-HDL-C	P=0.66	Not present
TGs	P=0.76	Not present
HDL-C	P=0.49	Not present
TC	P=0.63	Not present
SBP	P=0.15	Not present
DBP	P=0.57	Not present
IHCL	Unable to calculate*	
ALT	Unable to calculate*	
AST	Unable to calculate*	
Uric acid	Unable to calculate*	

2h-PG, two-hour post prandial glucose; ALT, alanine transaminase; AST, aspartate transaminase; BMI, body mass index; DBP, diastolic blood pressure; FPG, fasting plasma glucose; FPI, fasting plasma insulin; HbA1c, hemoglobin A1c; HDL-C, high-density lipoprotein-cholesterol; HOMA-IR, Homeostatic Model Assessment of Insulin Resistance; IHCL, intra-hepatocellular lipid; LDL-C, low-density lipoprotein-cholesterol; Non-HDL-C, non-high-density lipoprotein-cholesterol; SBP, systolic blood pressure; TC, total cholesterol; TGs, triglycerides.
* Calculation issues with loop-specific approach in Stata with multiple-arm trials as model did not converge, therefore, “unable to calculate” for all missing ones.

© 2022 McGlynn ND et al. JAMA Network Open.

eTable 7. Design by treatment approach for inconsistency

Outcome	P-value	Inconsistency
Body weight	0.99	Not present
BMI	0.71	Not present
Body fat	0.94	Not present
Waist circumference	0.88	Not present
HbA1c	0.83	Not present
FPG	0.85	Not present
2h-PG	0.06	Not present
FPI	0.27	Not present
HOMA-IR	0.45	Not present
LDL-C	0.28	Not present
Non-HDL-C	0.075	Not present
TGs	0.45	Not present
HDL-C	0.77	Not present
TC	0.08	Not present
SBP	0.77	Not present
DBP	0.27	Not present
IHCL	Unable to calculate*	Unable to assess*
ALT	0.51	Not present
AST	0.92	Not present
Uric acid	0.76	Not present

2h-PG, two-hour post prandial glucose; ALT, alanine transaminase; AST, aspartate transaminase; BMI, body mass index; DBP, diastolic blood pressure; FPG, fasting plasma glucose; FPI, fasting plasma insulin; HbA1c, hemoglobin A1c; HDL-C, high-density lipoprotein-cholesterol; HOMA-IR, Homeostatic Model Assessment of Insulin Resistance; IHCL, intra-hepatocellular lipid; LDL-C, low-density lipoprotein-cholesterol; Non-HDL-C, non-high-density lipoprotein-cholesterol; SBP, systolic blood pressure; TC, total cholesterol; TGs, triglycerides.
*Calculation issues with design-by-treatment inconsistency in Stata did not converge.

© 2022 McGlynn ND et al. JAMA Network Open.

eFigures

eFigure 1. Cochrane risk of bias summary for all included trials

	Random sequence generation (selection bias)	Allocation concealment (selection bias)	Blinding of participants and personnel (performance bias)	Incomplete outcome data (attrition bias)	Selective reporting (reporting bias)
Bonnet 2018	+	?	+	?	+
Bruun 2015	+	?	?	?	+
Campos 2015	+	?	?	?	+
Ebbeling 2020	+	?	+	+	+
Engel 2017	+	?	?	?	+
Hernandez-Cordero 2014	+	?	+	+	+
Higgins 2018 (1050mg ASP)	+	?	?	?	+
Higgins 2018 (350mg ASP)	+	?	?	?	+
Higgins 2019 (Asp)	+	?	+	−	+
Higgins 2019 (RebA)	+	?	+	−	+
Higgins 2019 (Saccharin)	+	?	+	−	+
Higgins 2019 (Sucralose)	+	?	+	−	+
Madjd 2015	+	+	+	+	+
Madjd 2017	+	+	+	+	+
Maersk 2012	+	?	?	?	+
Peters 2016	+	?	+	?	+
Reid 2007	?	?	+	?	+
Reid 2010	?	?	+	?	+
Reid 2014	−	?	+	+	+
Tate 2012	+	+	+	+	+
Tordoff 1990 (Females)	?	?	+	−	?
Tordoff 1990 (Males)	?	?	+	−	?

© 2022 McGlynn ND et al. JAMA Network Open.

eFigure 2. Risk of bias proportion for all included trials

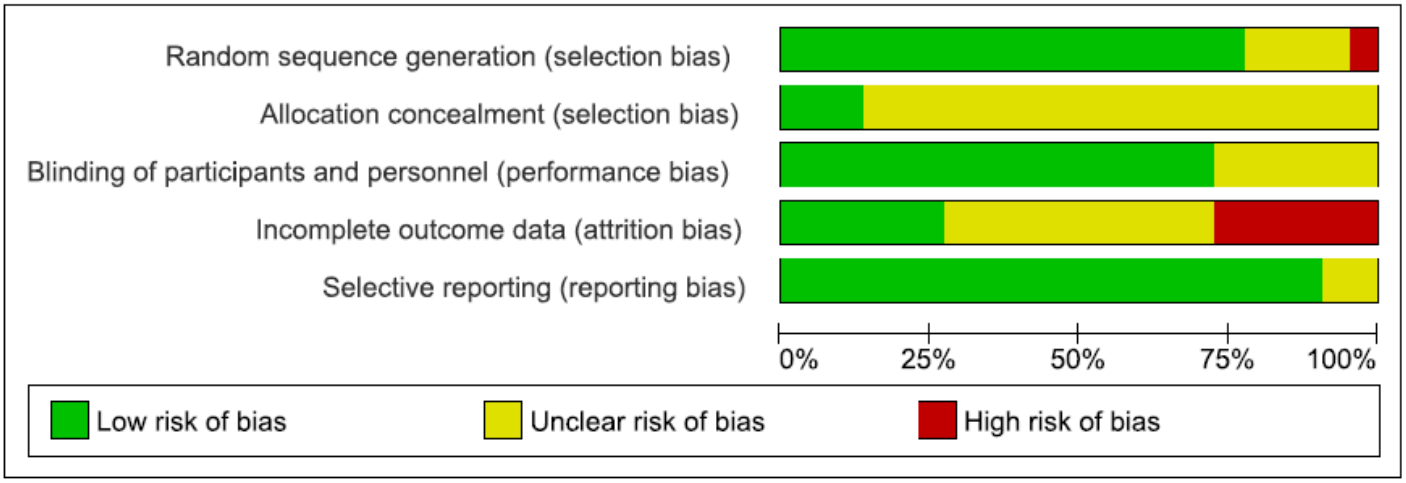

© 2022 McGlynn ND et al. JAMA Network Open.

eFigure 3. Transitivity analysis box plots showing the distribution of the mean age (years) of the trials across the available direct comparisons

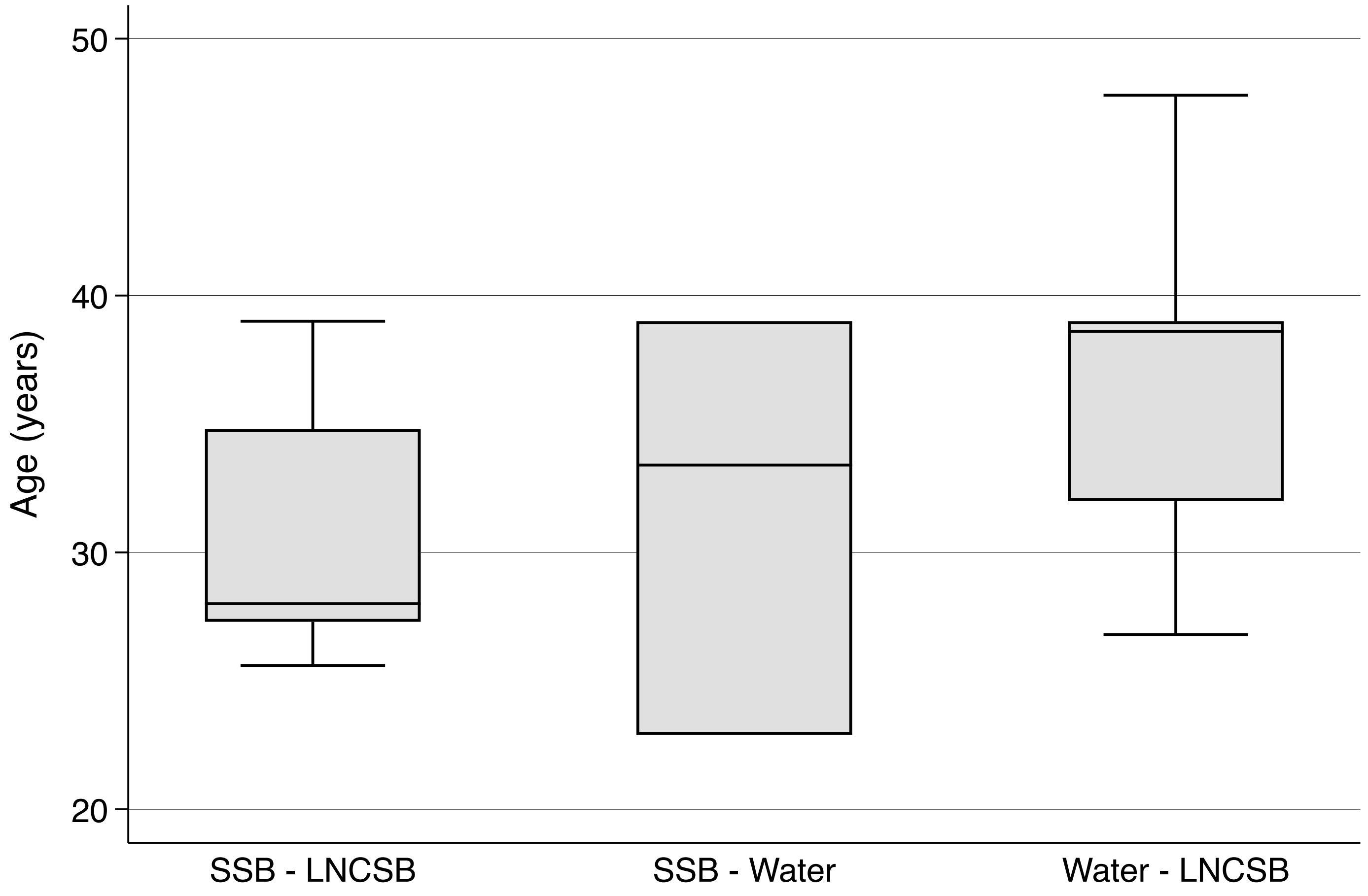

© 2022 McGlynn ND et al. JAMA Network Open.

eFigure 4. Transitivity analysis box plots showing the distribution of the study length (weeks) of the trials across the available direct comparisons

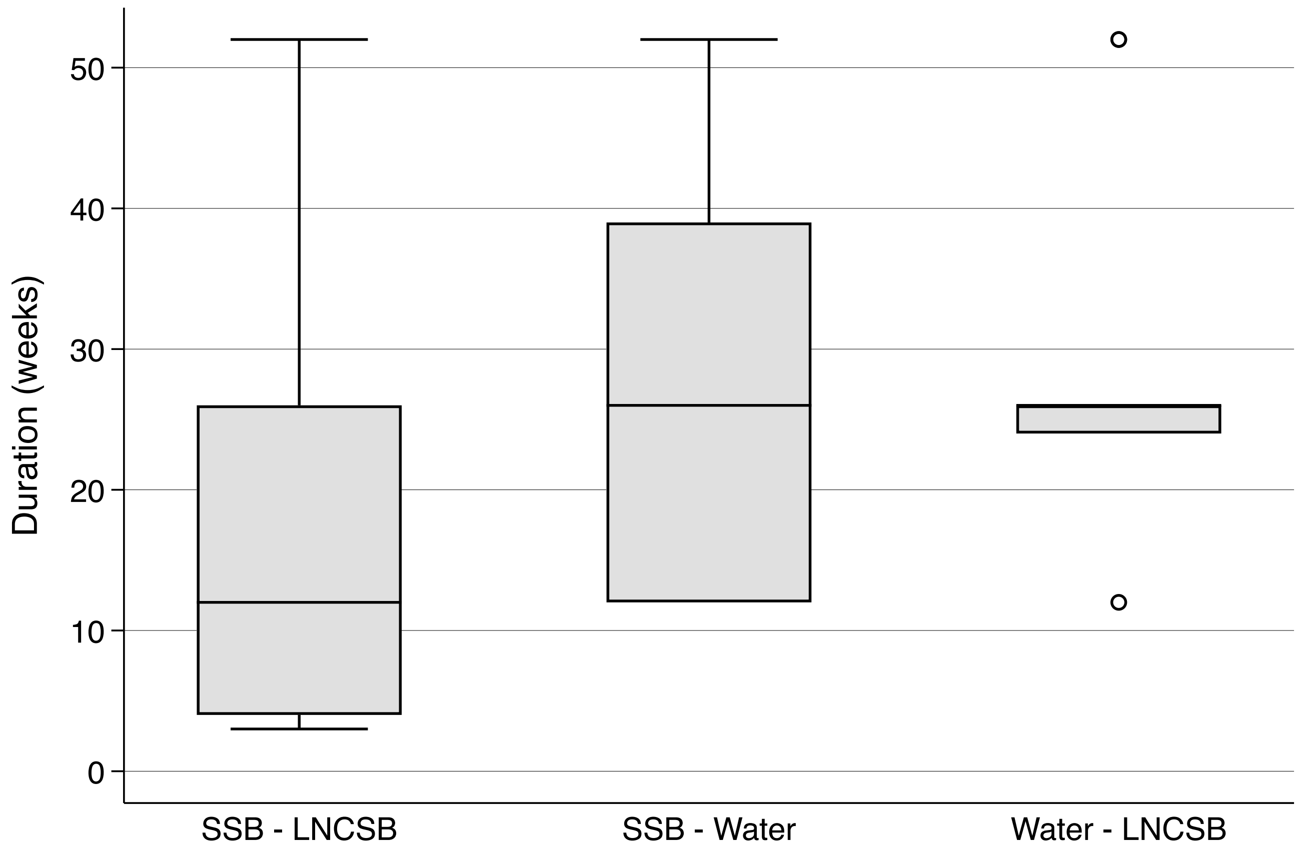

© 2022 McGlynn ND et al. JAMA Network Open.

eFigure 5. Transitivity analysis box plots showing the distribution of the sample size of the trials across the available direct comparisons

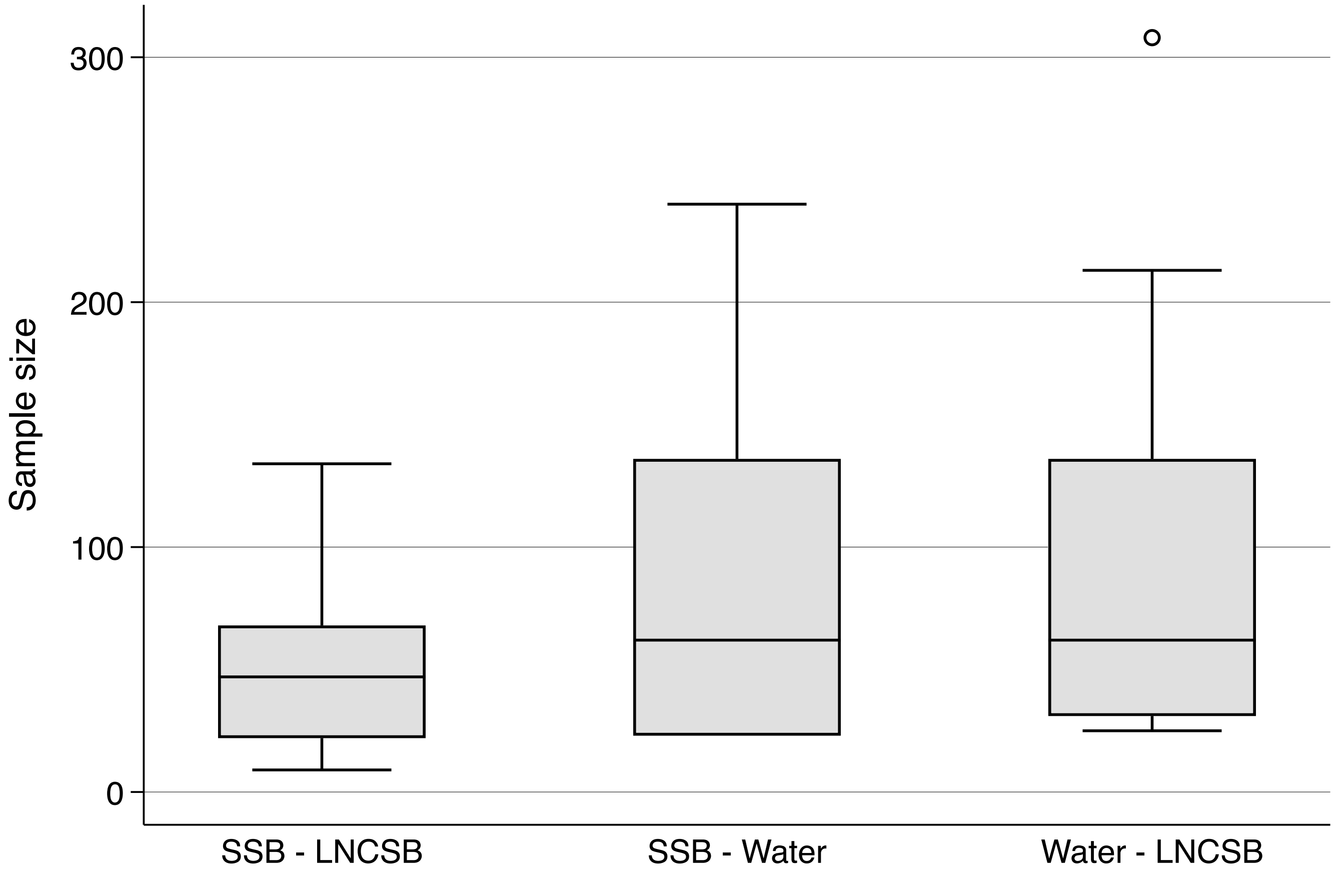

© 2022 McGlynn ND et al. JAMA Network Open.

eFigure 6. Transitivity analysis box plots showing the distribution of % males of the trials across the available direct comparisons

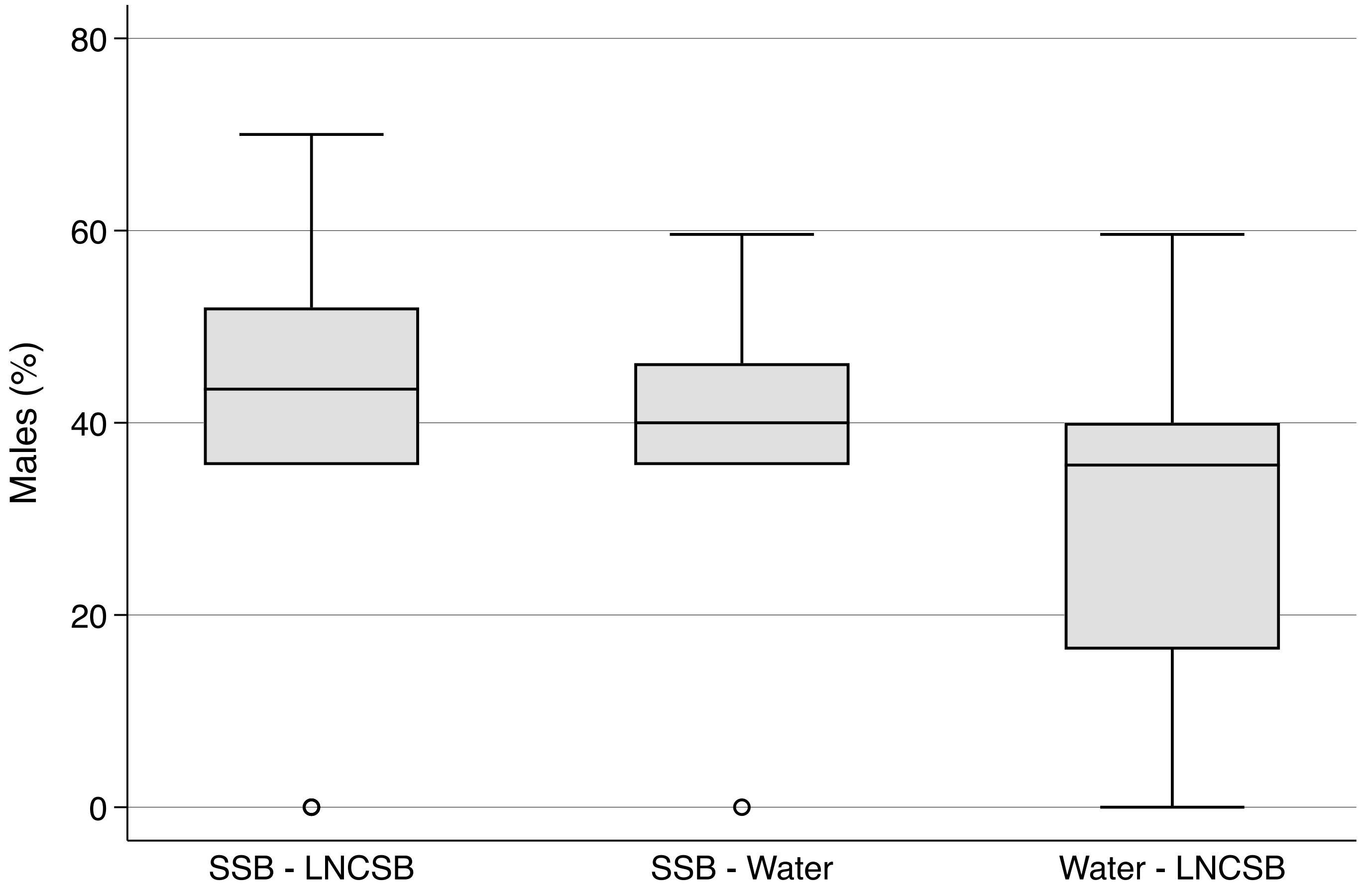

© 2022 McGlynn ND et al. JAMA Network Open.

eFigure 7. Network analysis with GRADE assessment of the certainty of the evidence comparing LNCSBs, SSBs and Water on Body Weight

Network Meta-Analysis

Body Weight (kg)

Comparison	Analysis	Trial Comparisons	Mean Difference with 95% CI	P_{Effect}	I^2 (%)	$P_{Inconsistency}$
**LNCSBs for SSBs**	**Network**	24	-1.057 [-1.705 to -0.408]	0.001		0.703
LNCSBs for SSBs	Direct	12	-1.015 [-1.745 to -0.285]		0	0.720
LNCSBs for SSBs	*Indirect*	12	-1.482 [-3.764 to 0.800]			
**Water for SSBs**	**Network**	24	0.012 [-0.952 to 0.976]	0.981		0.906
Water for SSBs	Direct	3	-0.061 [-1.569 to 1.448]		0	0.993
Water for SSBs	*Indirect*	21	0.066 [-1.380 to 1.512]			
**LNCSBs for Water**	**Network**	24	-1.069 [-1.953 to -0.185]	0.018		0.830
LNCSBs for Water	Direct	9	-1.120 [-2.202 to -0.039]		71	0.004
LNCSBs for Water	*Indirect*	15	-0.866 [-2.904 to 1.171]			

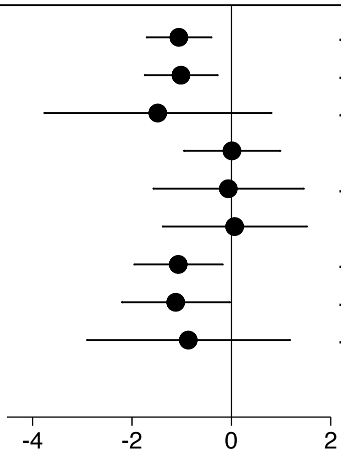

Using restricted maximum likelihood (REML) random-effects model

Note: $P_{Inconsistency}$ represents P-value for *incoherence factor* in the network estimates, and for *heterogeneity* in the direct pair-wise estimates

GRADE	Risk of Bias	Inconsistency	Indirectness	Imprecision	Publication bias	Overall
LNCSBs for SSBs	Not serious	Not serious	Not serious	Serious *Rated down the evidence for serious imprecision in the network estimates as the 95% CIs were wide and crossed prespecified MIDs for benefit and/or harm (0.5 kg for body weight).*	Not serious	**Moderate**
Water for SSBs	Not serious	Serious *Rated down the evidence for incoherence on visual inspection.*	Not serious	Serious *Rated down the evidence for serious imprecision in the network estimates as the 95% CIs were wide and crossed prespecified MIDs for benefit and/or harm (0.5 kg for body weight).*	Not serious	**Low**
LNCSBs for Water	Not serious	Serious *Rated down for inconsistency assessed in the direct estimates as substantial unexplained heterogeneity (I2 >50%, P<0.10)*	Not serious	Serious *Rated down the evidence for serious imprecision in the network estimates as the 95% CIs were wide and crossed prespecified MIDs for benefit and/or harm (0.5 kg for body weight).*	Not serious	**Low**

GRADE, Grading of Recommendations, Assessment, Development, and Evaluation; MID, Minimally Important Difference; NA, not available; LNCSBs, low- and no-calorie sweetened beverages; SSBs, sugar-sweetened beverages.

© 2022 McGlynn ND et al. JAMA Network Open.

eFigure 8. Network analysis with GRADE assessment of the certainty of the evidence comparing LNCSBs, SSBs and Water on BMI

Network Meta-Analysis

BMI (kg/m²)

Comparison	Analysis	Trial Comparisons	Mean Difference with 95% CI	P_{Effect}	I^2 (%)	$P_{Inconsistency}$
**LNCSBs for SSBs**	**Network**	14	-0.322 [-0.575 to -0.069]	0.012		0.247
LNCSBs for SSBs	Direct	9	-0.360 [-0.631 to -0.090]		35	0.252
LNCSBs for SSBs	*Indirect*	5	0.262 [-0.756 to 1.281]			
**Water for SSBs**	**Network**	14	-0.347 [-0.825 to 0.132]	0.156		0.250
Water for SSBs	Direct	2	-0.178 [-0.732 to 0.376]		0	0.923
Water for SSBs	*Indirect*	12	-0.787 [-1.669 to 0.095]			
**LNCSBs for Water**	**Network**	14	0.024 [-0.462 to 0.510]	0.922		0.257
LNCSBs for Water	Direct	3	0.406 [-0.413 to 1.225]		0	0.962
LNCSBs for Water	*Indirect*	11	-0.190 [-0.816 to 0.436]			

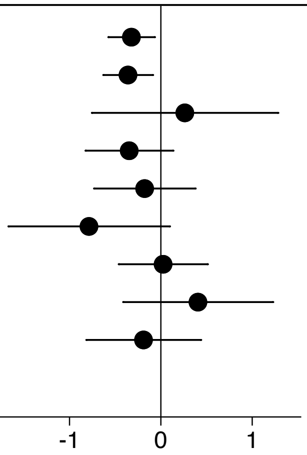

Using restricted maximum likelihood (REML) random-effects model

Note: $P_{Inconsistency}$ represents P-value for *incoherence factor* in the network estimates, and for *heterogeneity* in the direct pair-wise estimates

GRADE	Risk of Bias	Inconsistency	Indirectness	Imprecision	Publication bias	Overall
LNCSBs for SSBs	Not serious	Serious *Rated down the evidence for incoherence via visual inspection.*	Not serious	Serious *Rated down the evidence for serious imprecision in the network estimates as the 95% CIs were wide and crossed prespecified MIDs for benefit and/or harm (0.2kg/m2 for BMI)*	Not serious	**Low**
Water for SSBs	Not serious	Not serious	Not serious	Serious *Rated down the evidence for serious imprecision in the network estimates as the 95% CIs were wide and crossed prespecified MIDs for benefit and/or harm (0.2kg/m2 for BMI)*	Not serious	**Moderate**
LNCSBs for Water	Not serious	Serious *Rated down the evidence for incoherence on visual inspection.*	Not serious	Serious *Rated down the evidence for serious imprecision in the network estimates as the 95% CIs were wide and crossed prespecified MIDs for benefit and/or harm (0.2kg/m2 for BMI)*	Not serious	**Low**

GRADE, Grading of Recommendations, Assessment, Development, and Evaluation; MID, Minimally Important Difference; NA, not available; LNCSBs, low- and no-calorie sweetened beverages; SSBs, sugar-sweetened beverages.

© 2022 McGlynn ND et al. JAMA Network Open.

eFigure 9. Network analysis with GRADE assessment of the certainty of the evidence comparing LNCSBs, SSBs and Water on body fat (%)

Network Meta-Analysis

Body Fat (%)

Comparison	Analysis	Trial Comparisons	Mean Difference with 95% CI	P_{Effect}	I^2 (%)	$P_{Inconsistency}$
**LNCSBs for SSBs**	**Network**	14	-0.604 [-1.031 to -0.178]	0.005		0.444
LNCSBs for SSBs	Direct	7	-0.622 [-1.051 to -0.193]		0	0.998
LNCSBs for SSBs	*Indirect*	7	0.922 [-3.008 to 4.852]			
**Water for SSBs**	**Network**	14	-0.267 [-1.553 to 1.019]	0.684		0.612
Water for SSBs	Direct	3	-0.142 [-1.515 to 1.231]		0	0.696
Water for SSBs	*Indirect*	11	-1.145 [-4.772 to 2.483]			
**LNCSBs for Water**	**Network**	14	-0.337 [-1.673 to 0.998]	0.621		0.478
LNCSBs for Water	Direct	4	0.731 [-2.506 to 3.968]		0	0.960
LNCSBs for Water	*Indirect*	10	-0.555 [-2.020 to 0.909]			

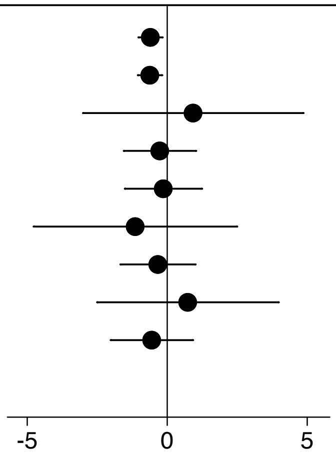

Using restricted maximum likelihood (REML) random-effects model

Note: $P_{Inconsistency}$ represents P-value for *incoherence factor* in the network estimates, and for *heterogeneity* in the direct pair-wise estimates

GRADE	Risk of Bias	Inconsistency	Indirectness	Imprecision	Publication bias	Overall
LNCSBs for SSBs	Not serious	Serious<br>*Rated down the evidence for incoherence via visual inspection.*	Not serious	Not serious<br>*(MIDs for body fat = 2%)*	Not serious	**MODERATE**
Water for SSBs	Not serious	Not serious	Not serious	Not serious<br>*(MIDs for body fat = 2%)*	Not serious	**HIGH**
LNCSBs for Water	Not serious	Serious<br>*Rated down the evidence for incoherence via visual inspection.*	Not serious	Not serious<br>*(MIDs for body fat = 2%)*	Not serious	**MODERATE**

GRADE, Grading of Recommendations, Assessment, Development, and Evaluation; MID, Minimally Important Difference; NA, not available; LNCSBs, low- and no-calorie sweetened beverages; SSBs, sugar-sweetened beverages.

© 2022 McGlynn ND et al. JAMA Network Open.

eFigure 10. Network analysis with GRADE assessment of the certainty of the evidence comparing LNCSBs, SSBs and Water on waist circumference

Network Meta-Analysis

Waist Circumference (cm)

Comparison	Analysis	Trial Comparisons	Mean Difference with 95% CI	P_{Effect}	I^2 (%)	$P_{Inconsistency}$
**LNCSBs for SSBs**	**Network**	6	-0.520 [-4.981 to 3.941]	0.819		
**Water for SSBs**	**Network**	6	0.300 [-3.681 to 4.281]	0.883		0.997
Water for SSBs	Direct	1	0.300 [-3.681 to 4.281]		.	.
Water for SSBs	*Indirect*	5	0.820 [-276.328 to 277.969]			
**LNCSBs for Water**	**Network**	6	-0.820 [-2.833 to 1.193]	0.425		
LNCSBs for Water	Direct	5	-0.820 [-2.834 to 1.193]		70	0.008
LNCSBs for Water	*Indirect*	1	0.220 [-554.440 to 554.880]			

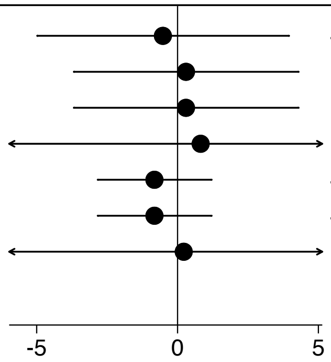

Using restricted maximum likelihood (REML) random-effects model

Note: $P_{Inconsistency}$ represents P-value for *incoherence factor* in the network estimates, and for *heterogeneity* in the direct pair-wise estimates

GRADE	Risk of Bias	Inconsistency	Indirectness	Imprecision	Publication bias	Overall
LNCSBs for SSBs	Not serious	Not serious	Serious *Rated down for indirectness as only based upon indirect assessment.*	Serious *Rated down the evidence for serious imprecision in the network estimates as the 95% CIs were wide and crossed prespecified MIDs for benefit and/or harm. MIDs for waist circumference = 2 cm).*	**NA**	**LOW**
Water for SSBs	Not serious	Not serious	Serious *Rated down for indirectness as only 1 direct study available.*	Serious *Rated down the evidence for serious imprecision in the network estimates as the 95% CIs were wide and crossed prespecified MIDs for benefit and/or harm. MIDs for waist circumference = 2 cm).*	**NA**	**LOW**
LNCSBs for Water	Not serious	Serious *Rated down for inconsistency assessed in the direct estimates as substantial unexplained heterogeneity (I2 >50%, P<0.10)*	Not serious.	Serious *Rated down the evidence for serious imprecision in the network estimates as the 95% CIs were wide and crossed prespecified MIDs for benefit and/or harm. MIDs for waist circumference = 2 cm).*	**NA**	**LOW**

GRADE, Grading of Recommendations, Assessment, Development, and Evaluation; MID, Minimally Important Difference; NA, not available; LNCSBs, low- and no-calorie sweetened beverages; SSBs, sugar-sweetened beverages.

© 2022 McGlynn ND et al. JAMA Network Open.

eFigure 11. Network analysis with GRADE assessment of the certainty of the evidence comparing LNCSBs, SSBs and Water on HbA1c

Network Meta-Analysis

HbA1c (%)

Comparison	Analysis	Trial Comparisons	Mean Difference with 95% CI	P_{Effect}	I^2 (%)	$P_{Inconsistency}$
**LNCSBs for SSBs**	**Network**	9	0.122 [-0.079 to 0.324]	0.234		0.821
LNCSBs for SSBs	Direct	4	0.110 [-0.137 to 0.357]		0	0.991
LNCSBs for SSBs	*Indirect*	5	0.173 [-0.316 to 0.662]			
**Water for SSBs**	**Network**	9	-0.085 [-0.327 to 0.156]	0.489		0.821
Water for SSBs	Direct	1	-0.050 [-0.480 to 0.380]		.	.
Water for SSBs	*Indirect*	8	-0.113 [-0.453 to 0.226]			
**LNCSBs for Water**	**Network**	9	0.208 [0.016 to 0.400]	0.034		0.821
LNCSBs for Water	Direct	4	0.223 [-0.010 to 0.456]		93	< 0.001
LNCSBs for Water	*Indirect*	5	0.160 [-0.336 to 0.656]			

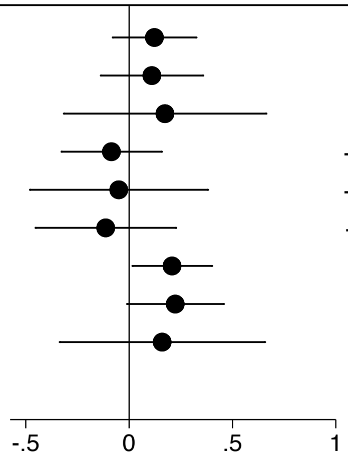

Using restricted maximum likelihood (REML) random-effects model

Note: $P_{Inconsistency}$ represents P-value for *incoherence factor* in the network estimates, and for *heterogeneity* in the direct pair-wise estimates

GRADE	Risk of Bias	Inconsistency	Indirectness	Imprecision	Publication Bias	Overall
LNCSBs for SSBs	Not serious	Not serious	Not serious	Serious<br>*Rated down for imprecision when the 95% CI were wide and crossed prespecified MIDs for benefit and/or harm. (MID = 0.3% for HbA1c),*	NA	**MODERATE**
Water for SSBs	Not serious	Not serious	Serious<br>*Rated down for indirectness as only 1 direct study available.*	Serious<br>*Rated down for imprecision when the 95% CI were wide and crossed prespecified MIDs for benefit and/or harm. (MID = 0.3% for HbA1c),*	NA	**LOW**
LNCSBs for Water	Not serious	Serious<br>*Rated down for inconsistency assessed in the direct estimates as substantial unexplained heterogeneity (I2 >50%, P<0.10)*	Not serious	Serious<br>*Rated down for imprecision when the 95% CI were wide and crossed prespecified MIDs for benefit and/or harm. (MID = 0.3% for HbA1c),*	NA	**LOW**

GRADE, Grading of Recommendations, Assessment, Development, and Evaluation; MID, Minimally Important Difference; NA, not available; LNCSBs, low- and no-calorie sweetened beverages; SSBs, sugar-sweetened beverages.

© 2022 McGlynn ND et al. JAMA Network Open.

eFigure 12. Network analysis with GRADE assessment of the certainty of the evidence comparing LNCSBs, SSBs and Water on fasting plasma glucose (FPG)

Network Meta-Analysis

Fasting Blood Glucose (mmol/L)

Comparison	Analysis	Trial Comparisons	Mean Difference with 95% CI	P_{Effect}	I^2 (%)	$P_{Inconsistency}$
**LNCSBs for SSBs**	**Network**	19	-0.064 [-0.155 to 0.026]	0.162		0.317
LNCSBs for SSBs	Direct	7	-0.085 [-0.184 to 0.014]		0	0.524
LNCSBs for SSBs	*Indirect*	12	0.038 [-0.182 to 0.258]			
**Water for SSBs**	**Network**	19	-0.045 [-0.137 to 0.048]	0.348		0.166
Water for SSBs	Direct	3	-0.006 [-0.114 to 0.102]		0	0.967
Water for SSBs	*Indirect*	16	-0.155 [-0.337 to 0.027]			
**LNCSBs for Water**	**Network**	19	-0.020 [-0.084 to 0.044]	0.545		0.336
LNCSBs for Water	Direct	9	-0.014 [-0.079 to 0.051]		0	0.676
LNCSBs for Water	*Indirect*	10	-0.194 [-0.554 to 0.166]			

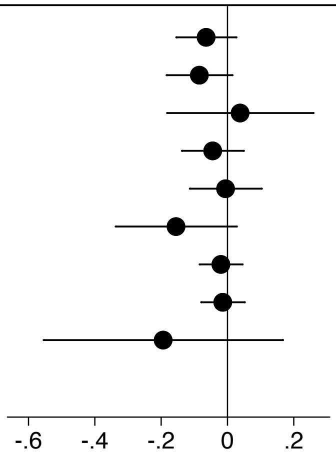

Using restricted maximum likelihood (REML) random-effects model

Note: $P_{Inconsistency}$ represents P-value for *incoherence factor* in the network estimates, and for *heterogeneity* in the direct pair-wise estimates

GRADE	Risk of Bias	Inconsistency	Indirectness	Imprecision	Publication Bias	Overall
LNCSBs for SSBs	Not serious	Serious<br>*Rated down the evidence for incoherence via visual inspection.*	Not serious	Not serious<br>*(MID for FPG = 0.5 mmol/L)*	Not serious	**MODERATE**
Water for SSBs	Not serious	Not serious	Not serious	Not serious<br>*(MID for FPG = 0.5 mmol/L)*	Not serious	**HIGH**
LNCSBs for Water	Not serious	Not serious	Not serious	Not serious<br>*(MID for FPG = 0.5 mmol/L)*	Not serious	**HIGH**

GRADE, Grading of Recommendations, Assessment, Development, and Evaluation; MID, Minimally Important Difference; NA, not available; LNCSBs, low- and no-calorie sweetened beverages; SSBs, sugar-sweetened beverages.

© 2022 McGlynn ND et al. JAMA Network Open.

eFigure 13. Network analysis with GRADE assessment of the certainty of the evidence comparing LNCSBs, SSBs and Water on 2-hour post-prandial glucose (2HPP)

Network Meta-Analysis
2-hour Post-prandial Glucose (mmol/L)

Comparison	Analysis	Trial Comparisons	Mean Difference with 95% CI	P_{Effect}	I^2 (%)	$P_{Inconsistency}$
**LNCSBs for SSBs**	**Network**	9	0.291 [-0.451 to 1.034]	0.442		0.998
LNCSBs for SSBs	Direct	4	0.291 [-0.451 to 1.034]		0	0.990
LNCSBs for SSBs	*Indirect*	5	-0.000 [-277.495 to 277.494]			
**Water for SSBs**	**Network**	9	0.101 [-0.666 to 0.869]	0.796		
**LNCSBs for Water**	**Network**	9	0.190 [-0.005 to 0.385]	0.056		0.998
LNCSBs for Water	Direct	5	0.190 [-0.005 to 0.385]		0	0.481
LNCSBs for Water	*Indirect*	4	0.771 [-550.062 to 551.603]			

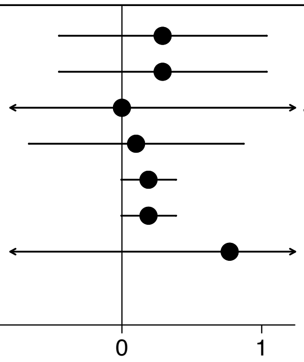

Using restricted maximum likelihood (REML) random-effects model

Note: $P_{Inconsistency}$ represents P-value for *incoherence factor* in the network estimates, and for *heterogeneity* in the direct pair-wise estimates

GRADE	Risk of Bias	Inconsistency	Indirectness	Imprecision	Publication Bias	Overall
LNCSBs for SSBs	Not serious	Not serious	Not serious	Serious<br>*Rated down for imprecision when the 95% CI were wide and crossed prespecified MIDs for benefit and/or harm. (MIDs for 2h-PG = 0.1%),*	NA	**MODERATE**
Water for SSBs	Not serious	Not serious	Serious<br>*Rated down for indirectness as only based upon indirect assessment.*	Serious<br>*Rated down for imprecision when the 95% CI were wide and crossed prespecified MIDs for benefit and/or harm. (MIDs for 2h-PG = 0.1%),*	NA	**LOW**
LNCSBs for Water	Not serious	Not serious	Not serious	Serious<br>*Rated down for imprecision when the 95% CI were wide and crossed prespecified MIDs for benefit and/or harm. (MIDs for 2h-PG = 0.1%),*	NA	**MODERATE**

GRADE, Grading of Recommendations, Assessment, Development, and Evaluation; MID, Minimally Important Difference; NA, not available; LNCSBs, low- and no-calorie sweetened beverages; SSBs, sugar-sweetened beverages.

© 2022 McGlynn ND et al. JAMA Network Open.

eFigure 14. Network analysis with GRADE assessment of the certainty of the evidence comparing LNCSBs, SSBs and Water on fasting insulin

Network Meta-Analysis

Fasting Insulin (pmol/L)

Comparison	Analysis	Trial Comparisons	Mean Difference with 95% CI	P_{Effect}	I^2 (%)	$P_{Inconsistency}$
**LNCSBs for SSBs**	**Network**	16	-9.794 [-29.986 to 10.399]	0.342		0.145
LNCSBs for SSBs	Direct	7	-13.984 [-35.022 to 7.053]		0	0.869
LNCSBs for SSBs	*Indirect*	9	52.043 [-33.410 to 137.497]			
**Water for SSBs**	**Network**	16	-17.396 [-39.495 to 4.704]	0.123		0.157
Water for SSBs	Direct	2	7.071 [-33.266 to 47.408]		3	0.310
Water for SSBs	*Indirect*	14	-32.870 [-63.612 to -2.128]			
**LNCSBs for Water**	**Network**	16	7.602 [-2.949 to 18.153]	0.158		0.445
LNCSBs for Water	Direct	7	7.813 [-2.995 to 18.621]		64	0.022
LNCSBs for Water	*Indirect*	9	-24.250 [-106.702 to 58.202]			

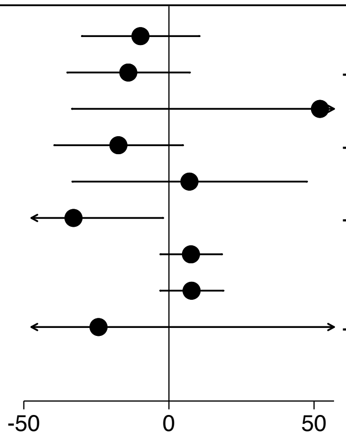

Using restricted maximum likelihood (REML) random-effects model

Note: $P_{Inconsistency}$ represents P-value for *incoherence factor* in the network estimates, and for *heterogeneity* in the direct pair-wise estimates

GRADE	Risk of Bias	Inconsistency	Indirectness	Imprecision	Publication Bias	Overall
LNCSBs for SSBs	Not serious	Serious<br>*Rated down the evidence for incoherence via visual inspection.*	Not serious.	Serious<br>*Rated down for imprecision when the 95% CI were wide and crossed prespecified MIDs for benefit and/or harm. (MIDs for FPI = 5pmol)*	Not serious	**LOW**
Water for SSBs	Not serious	Not serious<br>*Rated down for incoherence via visual inspection.*	Not serious.	Serious<br>*Rated down for imprecision when the 95% CI were wide and crossed prespecified MIDs for benefit and/or harm. (MIDs for FPI = 5pmol)*	Not serious	**LOW**
LNCSBs for Water	Not serious	Serious<br>*Rated down for inconsistency assessed in the direct estimates as substantial unexplained heterogeneity (I2 >50%, P<0.10)*	Not serious.	Serious<br>*Rated down for imprecision when the 95% CI were wide and crossed prespecified MIDs for benefit and/or harm. (MIDs for FPI = 5pmol)*	Not serious	**LOW**

GRADE, Grading of Recommendations, Assessment, Development, and Evaluation; MID, Minimally Important Difference; NA, not available; LNCSBs, low- and no-calorie sweetened beverages; SSBs, sugar-sweetened beverages.

© 2022 McGlynn ND et al. JAMA Network Open.

eFigure 15. Network analysis with GRADE assessment of the certainty of the evidence comparing LNCSBs, SSBs and Water on HOMA-IR

Network Meta-Analysis

HOMA-IR

Comparison	Analysis	Trial Comparisons	Mean Difference with 95% CI	P_{Effect}	I^2 (%)	$P_{Inconsistency}$
**LNCSBs for SSBs**	**Network**	7	-0.103 [-0.714 to 0.507]	0.740		0.285
LNCSBs for SSBs	Direct	2	-0.241 [-0.956 to 0.474]		0	0.431
LNCSBs for SSBs	*Indirect*	5	1.022 [-1.136 to 3.181]			
**Water for SSBs**	**Network**	7	-0.137 [-0.807 to 0.534]	0.690		0.197
Water for SSBs	Direct	1	0.321 [-0.652 to 1.295]		.	.
Water for SSBs	*Indirect*	6	-0.861 [-2.175 to 0.452]			
**LNCSBs for Water**	**Network**	7	0.033 [-0.335 to 0.402]	0.859		0.392
LNCSBs for Water	Direct	4	0.046 [-0.344 to 0.436]		66	0.035
LNCSBs for Water	*Indirect*	3	-1.567 [-5.248 to 2.115]			

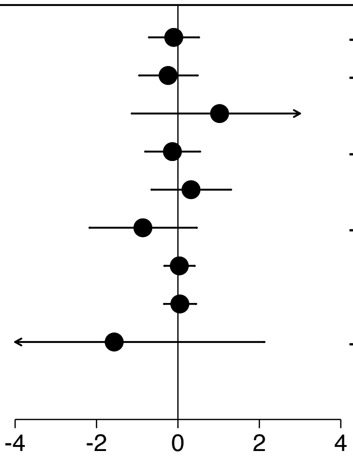

Using restricted maximum likelihood (REML) random-effects model

Note: $P_{Inconsistency}$ represents P-value for *incoherence factor* in the network estimates, and for *heterogeneity* in the direct pair-wise estimates

GRADE	Risk of Bias	Inconsistency	Indirectness	Imprecision	Publication Bias	Overall
LNCSBs for SSBs	Not serious	Serious<br>*Rated down the evidence for incoherence via visual inspection.*	Not serious	Not serious<br>*(MID for HOMA-IR.= 1)*	NA	**MODERATE**
Water for SSBs	Not serious	Serious<br>*Rated down the evidence for incoherence via visual inspection.*	Serious<br>*Rated down for indirectness as only 1 direct study available.*	Not serious<br>*(MID for HOMA-IR.= 1)*	NA	**LOW**
LNCSBs for Water	Not serious	Very Serious<br>*Rated down for inconsistency assessed in the direct estimates as substantial unexplained heterogeneity (I2 >50%, P<0.10)*<br>*Rated down for incoherence via visual inspection.*	Not serious	Not serious<br>*(MID for HOMA-IR.= 1)*	NA	**LOW**

GRADE, Grading of Recommendations, Assessment, Development, and Evaluation; MID, Minimally Important Difference; NA, not available; LNCSBs, low- and no-calorie sweetened beverages; SSBs, sugar-sweetened beverages.

© 2022 McGlynn ND et al. JAMA Network Open.

eFigure 16. Network analysis with GRADE assessment of the certainty of the evidence comparing LNCSBs, SSBs and Water on LDL-C

Network Meta-Analysis

LDL Cholesterol (mmol/L)

Comparison	Analysis	Trial Comparisons	Mean Difference with 95% CI	P_{Effect}	I^2 (%)	$P_{Inconsistency}$
**LNCSBs for SSBs**	**Network**	16	-0.013 [-0.148 to 0.123]	0.851		0.936
LNCSBs for SSBs	Direct	6	-0.015 [-0.180 to 0.149]		0	0.670
LNCSBs for SSBs	*Indirect*	10	-0.001 [-0.296 to 0.294]			
**Water for SSBs**	**Network**	16	-0.009 [-0.140 to 0.123]	0.896		0.563
Water for SSBs	Direct	3	-0.029 [-0.177 to 0.119]		0	0.529
Water for SSBs	*Indirect*	13	0.057 [-0.203 to 0.318]			
**LNCSBs for Water**	**Network**	16	-0.004 [-0.087 to 0.079]	0.922		0.760
LNCSBs for Water	Direct	7	-0.002 [-0.086 to 0.083]		3	0.435
LNCSBs for Water	*Indirect*	9	-0.071 [-0.508 to 0.365]			

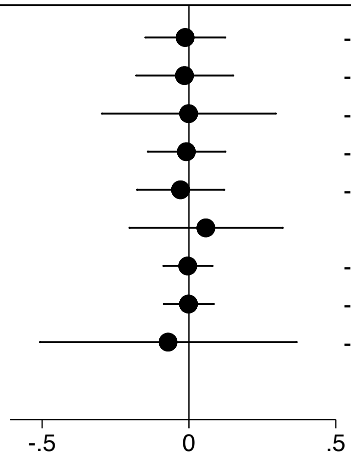

Using restricted maximum likelihood (REML) random-effects model

Note: $P_{Inconsistency}$ represents P-value for *incoherence factor* in the network estimates, and for *heterogeneity* in the direct pair-wise estimates

GRADE	Risk of Bias	Inconsistency	Indirectness	Imprecision	Publication Bias	Overall
LNCSBs for SSBs	Not serious	Not serious	Not serious.	Serious *Rated down for imprecision when the 95% CI were wide and crossed prespecified MIDs for benefit and/or harm. (MIDs for LDL-C = 0.1 mmol/L)*	Not serious	**MODERATE**
Water for SSBs	Not serious	Serious *Rated down for incoherence via visual inspection.*	Not serious.	Serious *Rated down for imprecision when the 95% CI were wide and crossed prespecified MIDs for benefit and/or harm. (MIDs for LDL-C = 0.1 mmol/L)*	Not serious	**LOW**
LNCSBs for Water	Not serious	Not serious	Not serious.	Not serious *(MIDs for LDL-C = 0.1 mmol/L)*	Not serious	**HIGH**

GRADE, Grading of Recommendations, Assessment, Development, and Evaluation; MID, Minimally Important Difference; NA, not available; LNCSBs, low- and no-calorie sweetened beverages; SSBs, sugar-sweetened beverages.

© 2022 McGlynn ND et al. JAMA Network Open.

eFigure 17. Network analysis with GRADE assessment of the certainty of the evidence comparing LNCSBs, SSBs and Water on Non-HDL-C

Network Meta-Analysis

Non-HDL Cholesterol (mmol/L)

Comparison	Analysis	Trial Comparisons	Mean Difference with 95% CI	P_{Effect}	I^2 (%)	$P_{Inconsistency}$
**LNCSBs for SSBs**	**Network**	14	-0.080 [-0.253 to 0.094]	0.368		0.395
LNCSBs for SSBs	Direct	6	-0.159 [-0.404 to 0.086]		22	0.266
LNCSBs for SSBs	*Indirect*	8	-0.009 [-0.254 to 0.235]			
**Water for SSBs**	**Network**	14	-0.058 [-0.219 to 0.103]	0.481		0.806
Water for SSBs	Direct	2	-0.049 [-0.256 to 0.159]		41	0.194
Water for SSBs	*Indirect*	12	-0.094 [-0.388 to 0.201]			
**LNCSBs for Water**	**Network**	14	-0.022 [-0.135 to 0.092]	0.707		0.978
LNCSBs for Water	Direct	6	-0.021 [-0.154 to 0.111]		48	0.073
LNCSBs for Water	*Indirect*	8	-0.027 [-0.385 to 0.332]			

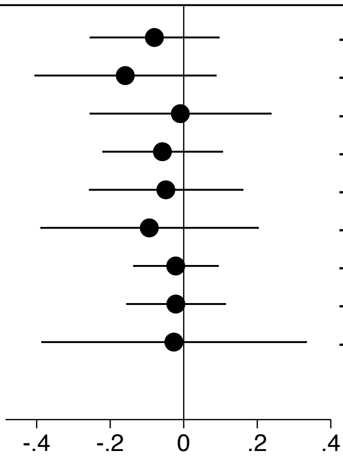

Using restricted maximum likelihood (REML) random-effects model

Note: $P_{Inconsistency}$ represents P-value for *incoherence factor* in the network estimates, and for *heterogeneity* in the direct pair-wise estimates

GRADE	Risk of Bias	Inconsistency	Indirectness	Imprecision	Publication Bias	Overall
LNCSBs for SSBs	Not serious	Not serious	Not serious.	Serious *Rated down for imprecision when the 95% CI were wide and crossed prespecified MIDs for benefit and/or harm. (MIDs for Non-HDL-C = 0.1 mmol/L)*	NA	**MODERATE**
Water for SSBs	Not serious	Not serious	Not serious.	Serious *Rated down for imprecision when the 95% CI were wide and crossed prespecified MIDs for benefit and/or harm. (MIDs for Non-HDL-C = 0.1 mmol/L)*	NA	**MODERATE**
LNCSBs for Water	Not serious	Not serious	Not serious.	Serious *Rated down for imprecision when the 95% CI were wide and crossed prespecified MIDs for benefit and/or harm. (MIDs for Non-HDL-C = 0.1 mmol/L)*	NA	**MODERATE**

GRADE, Grading of Recommendations, Assessment, Development, and Evaluation; MID, Minimally Important Difference; NA, not available; LNCSBs, low- and no-calorie sweetened beverages; SSBs, sugar-sweetened beverages.

© 2022 McGlynn ND et al. JAMA Network Open.

eFigure 18. Network analysis with GRADE assessment of the certainty of the evidence comparing LNCSBs, SSBs and Water on Triglycerides

Network Meta-Analysis

Triglycerides (mmol/L)

Comparison	Analysis	Trial Comparisons	Mean Difference with 95% CI	P_{Effect}	I^2 (%)	$P_{Inconsistency}$
**LNCSBs for SSBs**	**Network**	17	-0.131 [-0.286 to 0.025]	0.099		0.426
LNCSBs for SSBs	Direct	7	-0.098 [-0.277 to 0.081]		25	0.279
LNCSBs for SSBs	*Indirect*	10	-0.237 [-0.539 to 0.065]			
**Water for SSBs**	**Network**	17	-0.094 [-0.250 to 0.062]	0.236		0.562
Water for SSBs	Direct	3	-0.128 [-0.322 to 0.066]		43	0.156
Water for SSBs	*Indirect*	14	-0.030 [-0.308 to 0.249]			
**LNCSBs for Water**	**Network**	17	-0.037 [-0.129 to 0.056]	0.436		0.668
LNCSBs for Water	Direct	7	-0.041 [-0.138 to 0.056]		54	0.075
LNCSBs for Water	*Indirect*	10	0.062 [-0.401 to 0.526]			

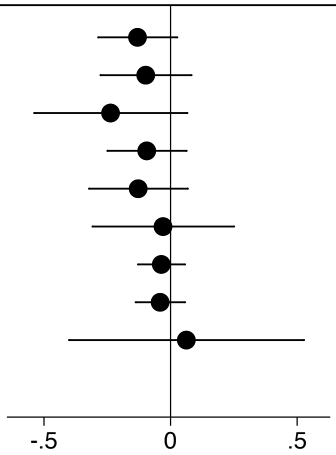

Using restricted maximum likelihood (REML) random-effects model

Note: $P_{Inconsistency}$ represents P-value for *incoherence factor* in the network estimates, and for *heterogeneity* in the direct pair-wise estimates

GRADE	Risk of Bias	Inconsistency	Indirectness	Imprecision	Publication Bias	Overall
LNCSBs for SSBs	Not serious	Not serious	Not serious.	Serious<br>*Rated down for imprecision when the 95% CI were wide and crossed prespecified MIDs for benefit and/or harm. (MIDs for TG = 0.1 mmol/L)*	Not serious	**MODERATE**
Water for SSBs	Not serious	Not serious	Not serious.	Serious<br>*Rated down for imprecision when the 95% CI were wide and crossed prespecified MIDs for benefit and/or harm. (MIDs for TG = 0.1 mmol/L)*	Not serious	**MODERATE**
LNCSBs for Water	Not serious	Very serious<br>*Rated down for inconsistency assessed in the direct estimates as substantial unexplained heterogeneity (I2 >50%, P<0.10)*<br>*Rated down for incoherence via visual inspection.*	Not serious	Serious<br>*Rated down for imprecision when the 95% CI were wide and crossed prespecified MIDs for benefit and/or harm. (MIDs for TG = 0.1 mmol/L)*	Not serious	**VERY LOW**

GRADE, Grading of Recommendations, Assessment, Development, and Evaluation; MID, Minimally Important Difference; NA, not available; LNCSBs, low- and no-calorie sweetened beverages; SSBs, sugar-sweetened beverages.

© 2022 McGlynn ND et al. JAMA Network Open.

eFigure 19. Network analysis with GRADE assessment of the certainty of the evidence comparing LNCSBs, SSBs and Water on HDL-C

Network Meta-Analysis

HDL Cholesterol (mmol/L)

Comparison	Analysis	Trial Comparisons	Mean Difference with 95% CI	P_{Effect}	I^2 (%)	$P_{Inconsistency}$
**LNCSBs for SSBs**	**Network**	17	0.048 [-0.008 to 0.104]	0.095		0.860
LNCSBs for SSBs	Direct	7	0.045 [-0.020 to 0.110]		0	0.928
LNCSBs for SSBs	*Indirect*	10	0.056 [-0.054 to 0.167]			
**Water for SSBs**	**Network**	17	0.036 [-0.020 to 0.091]	0.206		0.756
Water for SSBs	Direct	3	0.040 [-0.022 to 0.103]		0	0.941
Water for SSBs	*Indirect*	14	0.019 [-0.100 to 0.138]			
**LNCSBs for Water**	**Network**	17	0.012 [-0.028 to 0.052]	0.551		0.584
LNCSBs for Water	Direct	7	0.015 [-0.026 to 0.056]		0	0.920
LNCSBs for Water	*Indirect*	10	-0.037 [-0.217 to 0.143]			

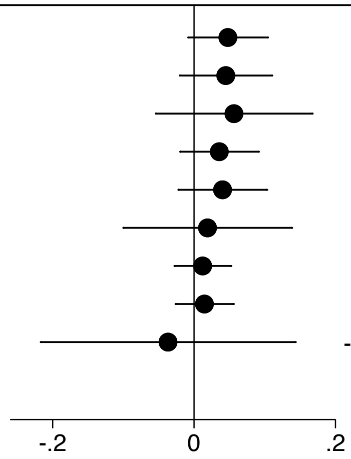

Using restricted maximum likelihood (REML) random-effects model

Note: $P_{Inconsistency}$ represents P-value for *incoherence factor* in the network estimates, and for *heterogeneity* in the direct pair-wise estimates

GRADE	Risk of Bias	Inconsistency	Indirectness	Imprecision	Publication Bias	Overall
LNCSBs for SSBs	Not serious	Not serious	Not serious.	Serious<br>*Rated down for imprecision when the 95% CI were wide and crossed prespecified MIDs for benefit and/or harm. (MIDs for HDL-C = 0.1 mmol/L)*	Not serious	**MODERATE**
Water for SSBs	Not serious	Not serious	Not serious.	Not serious<br>*(MIDs for HDL-C = 0.1 mmol/L)*	Not serious	**HIGH**
LNCSBs for Water	Not serious	Serious<br>*Rated down for incoherence via visual inspection.*	Not serious	Not serious<br>*MIDs for HDL-C = 0.1 mmol/L)*	Not serious	**LOW**

GRADE, Grading of Recommendations, Assessment, Development, and Evaluation; MID, Minimally Important Difference; NA, not available; LNCSBs, low- and no-calorie sweetened beverages; SSBs, sugar-sweetened beverages.

© 2022 McGlynn ND et al. JAMA Network Open.

eFigure 20. Network analysis with GRADE assessment of the certainty of the evidence comparing LNCSBs, SSBs and Water on total cholesterol

Network Meta-Analysis

Total Cholesterol (mmol/L)

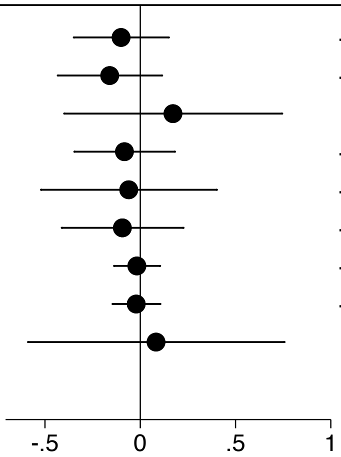

Comparison	Analysis	Trial Comparisons	Mean Difference with 95% CI	P_{Effect}	I^2 (%)	$P_{Inconsistency}$
**LNCSBs for SSBs**	**Network**	14	-0.101 [-0.349 to 0.148]	0.427		0.299
LNCSBs for SSBs	Direct	6	-0.160 [-0.433 to 0.113]		15	0.317
LNCSBs for SSBs	*Indirect*	8	0.172 [-0.399 to 0.743]			
**Water for SSBs**	**Network**	14	-0.083 [-0.346 to 0.180]	0.537		0.907
Water for SSBs	Direct	2	-0.061 [-0.521 to 0.400]		22	0.258
Water for SSBs	*Indirect*	12	-0.094 [-0.412 to 0.225]			
**LNCSBs for Water**	**Network**	14	-0.018 [-0.138 to 0.102]	0.772		0.765
LNCSBs for Water	Direct	6	-0.021 [-0.146 to 0.103]		29	0.182
LNCSBs for Water	*Indirect*	8	0.083 [-0.589 to 0.755]			

Using restricted maximum likelihood (REML) random-effects model

Note: $P_{Inconsistency}$ represents P-value for *incoherence factor* in the network estimates, and for *heterogeneity* in the direct pair-wise estimates

GRADE	Risk of Bias	Inconsistency	Indirectness	Imprecision	Publication Bias	Overall
LNCSBs for SSBs	Not serious	Serious *Rated down for incoherence via visual inspection.*	Not serious.	Serious *Rated down for imprecision when the 95% CI were wide and crossed prespecified MIDs for benefit and/or harm. (MIDs for TC = 0.1 mmol/L)*	Not serious	**LOW**
Water for SSBs	Not serious	Not serious	Not serious.	Serious *Rated down for imprecision when the 95% CI were wide and crossed prespecified MIDs for benefit and/or harm. (MIDs for TC = 0.1 mmol/L)*	Not serious	**MODERATE**
LNCSBs for Water	Not serious	Serious *Rated down for incoherence via visual inspection.*	Not serious	Serious *Rated down for imprecision when the 95% CI were wide and crossed prespecified MIDs for benefit and/or harm. (MIDs for TC = 0.1 mmol/L)*	Not serious	**LOW**

GRADE, Grading of Recommendations, Assessment, Development, and Evaluation; MID, Minimally Important Difference; NA, not available; LNCSBs, low- and no-calorie sweetened beverages; SSBs, sugar-sweetened beverages.

© 2022 McGlynn ND et al. JAMA Network Open.

eFigure 21. Network analysis with GRADE assessment of the certainty of the evidence comparing LNCSBs, SSBs and Water on SBP

Network Meta-Analysis

Systolic BP (mmHg)

Comparison	Analysis	Trial Comparisons	Mean Difference with 95% CI	P_{Effect}	I^2 (%)	$P_{Inconsistency}$
**LNCSBs for SSBs**	**Network**	10	-2.439 [-5.204 to 0.326]	0.084		0.413
LNCSBs for SSBs	Direct	3	-3.387 [-7.155 to 0.380]		0	0.593
LNCSBs for SSBs	*Indirect*	7	-0.539 [-6.089 to 5.010]			
**Water for SSBs**	**Network**	10	0.190 [-2.344 to 2.724]	0.883		0.632
Water for SSBs	Direct	3	0.364 [-2.618 to 3.347]		0	0.555
Water for SSBs	*Indirect*	7	-1.675 [-9.547 to 6.198]			
**LNCSBs for Water**	**Network**	10	-2.628 [-4.707 to -0.550]	0.013		0.377
LNCSBs for Water	Direct	4	-2.397 [-4.614 to -0.179]		29	0.275
LNCSBs for Water	*Indirect*	6	-6.547 [-15.503 to 2.410]			

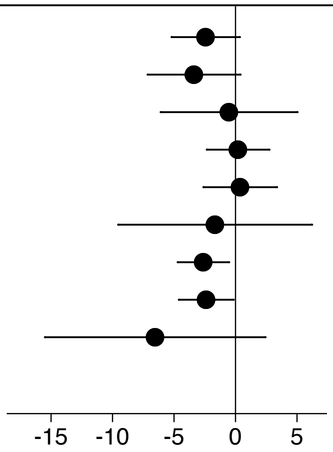

Using restricted maximum likelihood (REML) random-effects model

Note: $P_{Inconsistency}$ represents P-value for *incoherence factor* in the network estimates, and for *heterogeneity* in the direct pair-wise estimates

GRADE	Risk of Bias	Inconsistency	Indirectness	Imprecision	Publication Bias	Overall
LNCSBs for SSBs	Not serious	Not serious	Not serious.	Serious *Rated down for imprecision when the 95% CI were wide and crossed prespecified MIDs for benefit and/or harm. (MIDs for SBP = 2 mmHg)*	Not serious	**MODERATE**
Water for SSBs	Not serious	Serious *Rated down for incoherence via visual inspection.*	Not serious.	Serious *Rated down for imprecision when the 95% CI were wide and crossed prespecified MIDs for benefit and/or harm. (MIDs for SBP = 2 mmHg)*	Not serious	**LOW**
LNCSBs for Water	Not serious	Serious *Rated down for incoherence via visual inspection*	Not serious	Serious *Rated down for imprecision when the 95% CI were wide and crossed prespecified MIDs for benefit and/or harm. (MIDs for SBP = 2 mmHg)*	Not serious	**LOW**

GRADE, Grading of Recommendations, Assessment, Development, and Evaluation; MID, Minimally Important Difference; NA, not available; LNCSBs, low- and no-calorie sweetened beverages; SSBs, sugar-sweetened beverages.

© 2022 McGlynn ND et al. JAMA Network Open.

eFigure 22. Network analysis with GRADE assessment of the certainty of the evidence comparing LNCSBs, SSBs and Water on DBP

Network Meta-Analysis

Diastolic BP (mmHg)

Comparison	Analysis	Trial Comparisons	Mean Difference with 95% CI	P_{Effect}	I^2 (%)	$P_{Inconsistency}$
**LNCSBs for SSBs**	**Network**	9	-1.839 [-4.072 to 0.394]	0.106		0.246
LNCSBs for SSBs	Direct	3	-2.588 [-5.154 to -0.022]		18	0.334
LNCSBs for SSBs	*Indirect*	6	0.487 [-4.033 to 5.008]			
**Water for SSBs**	**Network**	9	-1.578 [-3.611 to 0.455]	0.128		0.550
Water for SSBs	Direct	3	-1.324 [-3.700 to 1.053]		1	0.469
Water for SSBs	*Indirect*	6	-3.474 [-10.071 to 3.123]			
**LNCSBs for Water**	**Network**	9	-0.261 [-2.118 to 1.595]	0.783		0.199
LNCSBs for Water	Direct	3	0.071 [-1.853 to 1.996]		0	0.332
LNCSBs for Water	*Indirect*	6	-4.561 [-11.380 to 2.258]			

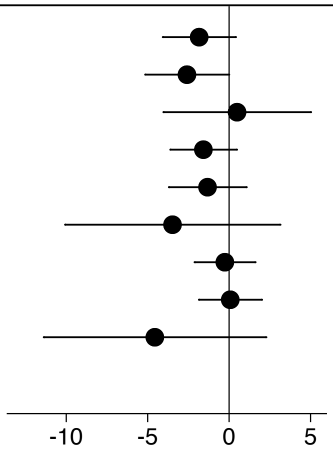

Using restricted maximum likelihood (REML) random-effects model

Note: $P_{Inconsistency}$ represents P-value for *incoherence factor* in the network estimates, and for *heterogeneity* in the direct pair-wise estimates

GRADE	Risk of Bias	Inconsistency	Indirectness	Imprecision	Publication Bias	Overall
LNCSBs for SSBs	Not serious	Serious<br>*Rated down for incoherence via visual inspection*	Not serious.	Serious<br>*Rated down for imprecision when the 95% CI were wide and crossed prespecified MIDs for benefit and/or harm. (MIDs for DBP = 2 mmHg)*	NA	**LOW**
Water for SSBs	Not serious	Not serious	Not serious.	Serious<br>*Rated down for imprecision when the 95% CI were wide and crossed prespecified MIDs for benefit and/or harm. (MIDs for DBP = 2 mmHg)*	NA	**MODERATE**
LNCSBs for Water	Not serious	Serious<br>*Rated down for incoherence via visual inspection*	Not serious	Serious<br>*Rated down for imprecision when the 95% CI were wide and crossed prespecified MIDs for benefit and/or harm. (MIDs for DBP = 2 mmHg)*	**NA**	**LOW**

GRADE, Grading of Recommendations, Assessment, Development, and Evaluation; MID, Minimally Important Difference; NA, not available; LNCSBs, low- and no-calorie sweetened beverages; SSBs, sugar-sweetened beverages.

© 2022 McGlynn ND et al. JAMA Network Open.

eFigure 23. Network analysis with GRADE assessment of the certainty of the evidence comparing LNCSBs, SSBs and Water on IHCL

Network Meta-Analysis

Liver Fat (IHCL) as SMD

**Comparison**	**Analysis**	**Trial Comparisons**	Mean Difference with 95% CI	P_{Effect}	I^2 (%)	$P_{Inconsistency}$
**LNCSBs for SSBs**	**Network**	4	-0.421 [-0.697 to -0.144]	0.003		
**Water for SSBs**	**Network**	4	-0.363 [-0.739 to 0.013]	0.059		0.789
SSB vs Water	Direct	1	-0.390 [-0.803 to 0.023]		.	.
SSB vs Water	*Indirect*	3	-0.210 [-1.431 to 1.011]			
**LNCSBs for Water**	**Network**	4	-0.058 [-0.424 to 0.309]	0.758		0.753
LNCSBs for Water	Direct	1	-0.080 [-0.472 to 0.312]		.	.
LNCSBs for Water	*Indirect*	3	0.100 [-0.949 to 1.149]			

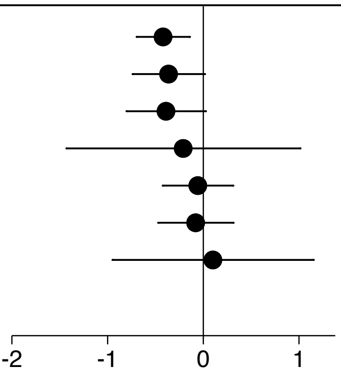

Using restricted maximum likelihood (REML) random-effects model

Note: $P_{Inconsistency}$ represents P-value for *incoherence factor* in the network estimates, and for *heterogeneity* in the direct pair-wise estimates

**GRADE**	**Risk of Bias**	**Inconsistency**	**Indirectness**	**Imprecision**	**Publication Bias**	**Overall**
LNCSBs for SSBs	Not serious	Not serious	Not serious	Serious *Rated down for imprecision when the 95% CI were wide and crossed prespecified MIDs for benefit and/or harm. (MIDs for IHCL = 0.1)*	NA	**MODERATE**
Water for SSBs	Not serious	Not serious	Serious *Rated down for indirectness as only 1 direct study available.*	Serious *Rated down for imprecision when the 95% CI were wide and crossed prespecified MIDs for benefit and/or harm. (MIDs for IHCL = 0.1)*	NA	**LOW**
LNCSBs for Water	Not serious	Serious *Rated down for incoherence via visual inspection*	Serious *Rated down for indirectness as only 1 direct study available.*	Not serious	NA	**LOW**

GRADE, Grading of Recommendations, Assessment, Development, and Evaluation; MID, Minimally Important Difference; NA, not available; LNCSBs, low- and no-calorie sweetened beverages; SSBs, sugar-sweetened beverages.

© 2022 McGlynn ND et al. JAMA Network Open.

eFigure 24. Network analysis with GRADE assessment of the certainty of the evidence comparing LNCSBs, SSBs and Water on ALT

Network Meta-Analysis

ALT (U/L)

Comparison	Analysis	Trial Comparisons	Mean Difference with 95% CI	P_{Effect}	I^2 (%)	$P_{Inconsistency}$
**LNCSBs for SSBs**	**Network**	6	-6.670 [-16.201 to 2.860]	0.170		0.280
LNCSB vs SSB	Direct	2	-8.456 [-18.523 to 1.611]		2	0.312
LNCSB vs SSB	*Indirect*	4	6.470 [-19.225 to 32.164]			
**Water for SSBs**	**Network**	6	-7.182 [-17.009 to 2.644]	0.152		0.645
SSB vs Water	Direct	1	-4.576 [-19.394 to 10.243]		.	.
SSB vs Water	*Indirect*	5	-9.129 [-21.980 to 3.723]			
**LNCSBs for Water**	**Network**	6	0.512 [-2.924 to 3.948]	0.770		0.541
LNCSBs for Water	Direct	3	0.418 [-3.031 to 3.868]		0	0.294
LNCSBs for Water	*Indirect*	3	12.313 [-25.666 to 50.293]			

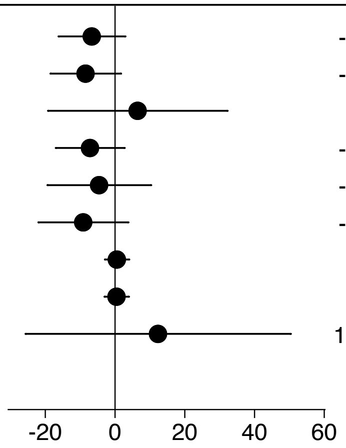

Using restricted maximum likelihood (REML) random-effects model

Note: $P_{Inconsistency}$ represents P-value for *incoherence factor* in the network estimates, and for *heterogeneity* in the direct pair-wise estimates

GRADE	Risk of Bias	Inconsistency	Indirectness	Imprecision	Publication Bias	Overall
LNCSBs for SSBs	Not serious	Serious<br>*Rated down for incoherence via visual inspection*	Not serious.	Serious<br>*Rated down for imprecision when the 95% CI were wide and crossed prespecified MIDs for benefit and/or harm. (MIDs for ALT = 1)*	NA	**LOW**
Water for SSBs	Not serious	Not serious	Serious<br>*Rated down for indirectness as only 1 direct study available.*	Serious<br>*Rated down for imprecision when the 95% CI were wide and crossed prespecified MIDs for benefit and/or harm. (MIDs for ALT = 1)*	NA	**LOW**
LNCSBs for Water	Not serious	Not serious	Not serious	Not serious	NA	**LOW**

GRADE, Grading of Recommendations, Assessment, Development, and Evaluation; MID, Minimally Important Difference; NA, not available; LNCSBs, low- and no-calorie sweetened beverages; SSBs, sugar-sweetened beverages.

© 2022 McGlynn ND et al. JAMA Network Open.

eFigure 25. Network analysis with GRADE assessment of the certainty of the evidence comparing LNCSBs, SSBs and Water on AST

Network Meta-Analysis

AST (U/L)

Comparison	Analysis	Trial Comparisons	Mean Difference with 95% CI	P_{Effect}	I^2 (%)	$P_{Inconsistency}$
**LNCSBs for SSBs**	**Network**	3	-1.500 [-7.869 to 4.870]	0.644		0.995
LNCSBs for SSBs	Direct	1	-1.500 [-7.870 to 4.870]		.	.
LNCSBs for SSBs	*Indirect*	2	-0.001 [-438.254 to 438.253]			
**Water for SSBs**	**Network**	3	-1.700 [-9.352 to 5.952]	0.663		
**LNCSBs for Water**	**Network**	3	0.200 [-4.041 to 4.441]	0.926		0.995
LNCSBs for Water	Direct	2	0.200 [-4.041 to 4.441]		0	0.817
LNCSBs for Water	*Indirect*	1	-2.800 [-879.330 to 873.730]			

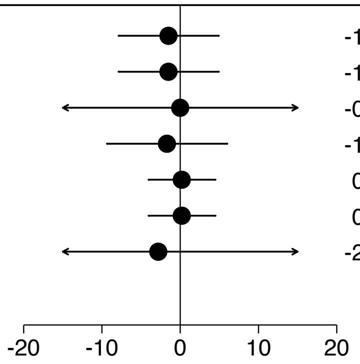

Using restricted maximum likelihood (REML) random-effects model

Note: $P_{Inconsistency}$ represents P-value for *incoherence factor* in the network estimates, and for *heterogeneity* in the direct pair-wise estimates

GRADE	Risk of Bias	Inconsistency	Indirectness	Imprecision	Publication Bias	Overall
LNCSBs for SSBs	Not serious	Not serious	Serious *Rated down for indirectness as only 1 direct study available.*	Serious *Rated down for imprecision when the 95% CI were wide and crossed prespecified MIDs for benefit and/or harm. (MIDs for AST = 1)*	NA	**LOW**
Water for SSBs	Not serious	Not serious	Serious *Rated down for indirectness as evidence only comes from indirect studies..*	Serious *Rated down for imprecision when the 95% CI were wide and crossed prespecified MIDs for benefit and/or harm. (MIDs for AST = 1)*	NA	**LOW**
LNCSBs for Water	Not serious	Serious *Rated down for incoherence via visual inspection*	Not serious	Serious *Rated down for imprecision when the 95% CI were wide and crossed prespecified MIDs for benefit and/or harm. (MIDs for AST = 1)*	NA	**LOW**

GRADE, Grading of Recommendations, Assessment, Development, and Evaluation; MID, Minimally Important Difference; NA, not available; LNCSBs, low- and no-calorie sweetened beverages; SSBs, sugar-sweetened beverages.

© 2022 McGlynn ND et al. JAMA Network Open.

eFigure 26. Network analysis with GRADE assessment of the certainty of the evidence comparing LNCSBs, SSBs and Water on uric acid

Network Meta-Analysis

Uric Acid (mmol/L)

Comparison	Analysis	Trial Comparisons	Mean Difference with 95% CI	P_{Effect}	I^2 (%)	$P_{Inconsistency}$
**LNCSBs for SSBs**	**Network**	7	-0.015 [-0.051 to 0.020]	0.396		
**Water for SSBs**	**Network**	7	-0.014 [-0.054 to 0.025]	0.477		0.676
Water for SSBs	Direct	2	-0.017 [-0.062 to 0.029]		86	0.007
Water for SSBs	*Indirect*	5	0.023 [-0.160 to 0.206]			
**LNCSBs for Water**	**Network**	7	-0.001 [-0.044 to 0.042]	0.962		0.676
LNCSBs for Water	Direct	2	-0.003 [-0.052 to 0.046]		0	0.527
LNCSBs for Water	*Indirect*	5	0.037 [-0.144 to 0.218]			

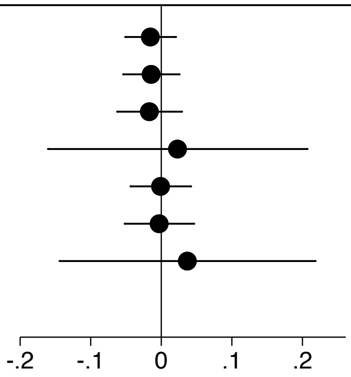

Using restricted maximum likelihood (REML) random-effects model

Note: $P_{Inconsistency}$ represents P-value for *incoherence factor* in the network estimates, and for *heterogeneity* in the direct pair-wise estimates

GRADE	Risk of Bias	Inconsistency	Indirectness	Imprecision	Publication Bias	Overall
LNCSBs for SSBs	Not serious	Not serious	Not serious	Serious<br>*Rated down for imprecision when the 95% CI were wide and crossed prespecified MIDs for benefit and/or harm. (MIDs for Uric acid = 0.013 mmol/L)*	NA	**MODERATE**
Water for SSBs	Not serious	Very Serious<br>*Rated down for inconsistency assessed in the direct estimates as substantial unexplained heterogeneity (I2 >50%, P<0.10)*<br>*Rated down for incoherence via visual inspection*	Not serious	Serious<br>*Rated down for imprecision when the 95% CI were wide and crossed prespecified MIDs for benefit and/or harm. (MIDs for Uric acid = 0.013 mmol/L)*	NA	**VERY LOW**
LNCSBs for Water	Not serious	Very Serious<br>*Rated down for inconsistency assessed in the direct estimates as substantial unexplained heterogeneity (I2 >50%, P<0.10)*<br>*Rated down for incoherence via visual inspection*	Not serious	Serious<br>*Rated down for imprecision when the 95% CI were wide and crossed prespecified MIDs for benefit and/or harm. (MIDs for Uric acid = 0.013 mmol/L)*	NA	**VERY LOW**

GRADE, Grading of Recommendations, Assessment, Development, and Evaluation; MID, Minimally Important Difference; NA, not available; LNCSBs, low- and no-calorie sweetened beverages; SSBs, sugar-sweetened beverages.

© 2022 McGlynn ND et al. JAMA Network Open.

eFigure 27. Network diagram for randomized controlled trials investigating the association of the substitution of LNCSBs for SSBs, water for SSBs, and LNCSBs for water with Body Weight

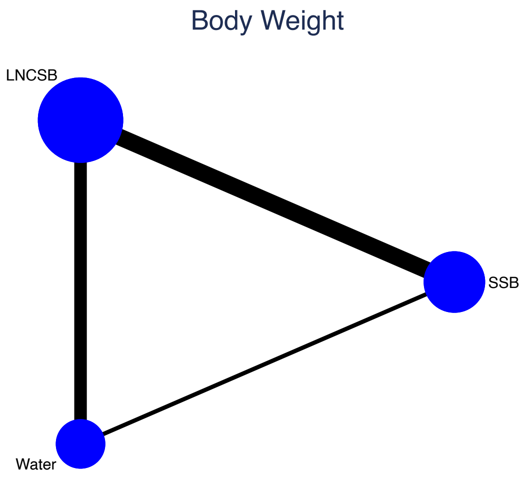

Blue nodes represent the study size for each beverage under investigation. The thickness of the black lines represents the number of studies directly comparing one beverage to another. LNCSBs, non-nutritive sweetened beverages; SSBs, sugar-sweetened beverages.

© 2022 McGlynn ND et al. JAMA Network Open.

eFigure 28. Network diagram for randomized controlled trials investigating the association of the substitution of LNCSBs for SSBs, water for SSBs, and LNCSBs for water with BMI

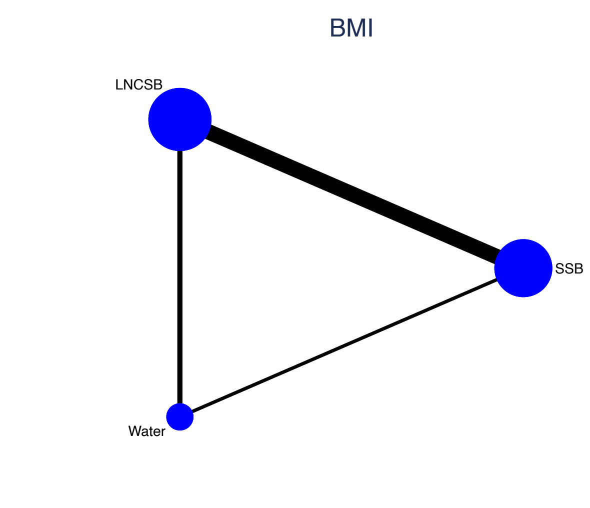

Blue nodes represent the study size for each beverage under investigation. The thickness of the black lines represents the number of studies directly comparing one beverage to another. BMI; body mass index, LNCSBs, non-nutritive sweetened beverages; SSBs, sugar-sweetened beverages.

© 2022 McGlynn ND et al. JAMA Network Open.

eFigure 29. Network diagram for randomized controlled trials investigating the association of the substitution of LNCSBs for SSBs, water for SSBs, and LNCSBs for water with Body Fat %

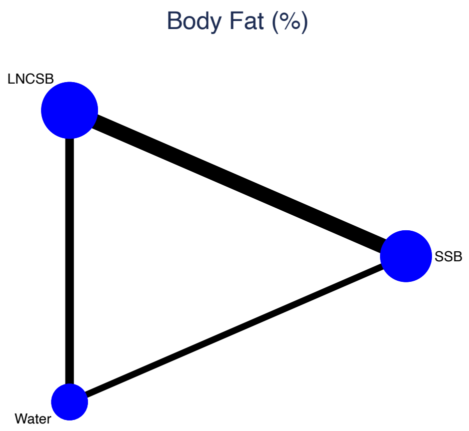

Blue nodes represent the study size for each beverage under investigation. The thickness of the black lines represents the number of studies directly comparing one beverage to another. LNCSBs, non-nutritive sweetened beverages; SSBs, sugar-sweetened beverages.

© 2022 McGlynn ND et al. JAMA Network Open.

eFigure 30. Network diagram for randomized controlled trials investigating the association of the substitution of LNCSBs for SSBs, water for SSBs, and LNCSBs for water with Waist Circumference

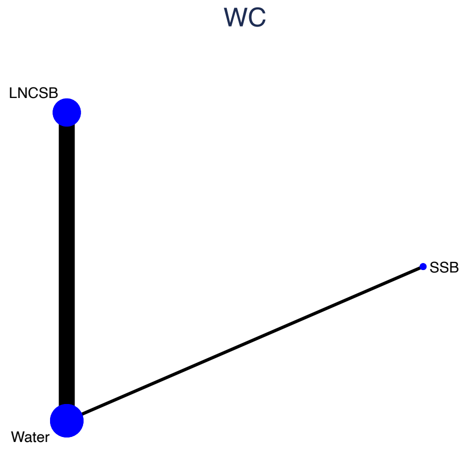

Blue nodes represent the study size for each beverage under investigation. The thickness of the black lines represents the number of studies directly comparing one beverage to another. LNCSBs, non-nutritive sweetened beverages; SSBs, sugar-sweetened beverages.

© 2022 McGlynn ND et al. JAMA Network Open.

eFigure 31. Network diagram for randomized controlled trials investigating the association of the substitution of LNCSBs for SSBs, water for SSBs, and LNCSBs for water with HbA1c

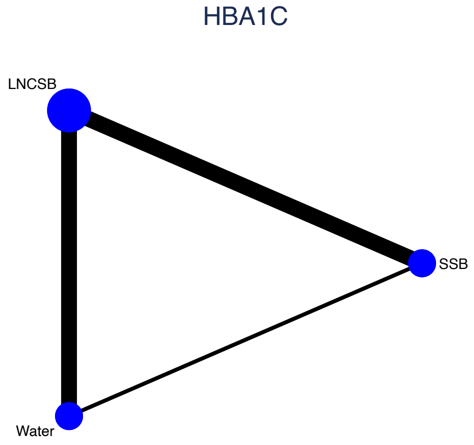

Blue nodes represent the study size for each beverage under investigation. The thickness of the black lines represents the number of studies directly comparing one beverage to another. HbA1c, hemoglobin A1c; LNCSBs, non-nutritive sweetened beverages; SSBs, sugar-sweetened beverages.

© 2022 McGlynn ND et al. JAMA Network Open.

eFigure 32. Network diagram for randomized controlled trials investigating the association of the substitution of LNCSBs for SSBs, water for SSBs, and LNCSBs for water with FPG

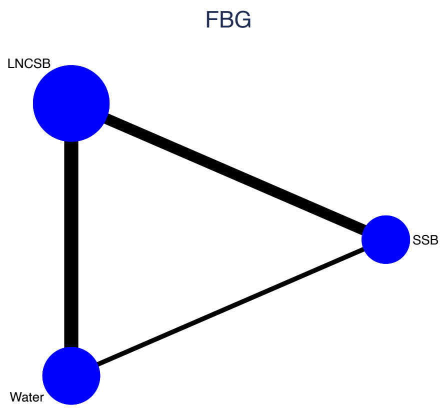

Blue nodes represent the study size for each beverage under investigation. The thickness of the black lines represents the number of studies directly comparing one beverage to another. FPG, fasting plasma glucose; LNCSBs, non-nutritive sweetened beverages; SSBs, sugar-sweetened beverages.

© 2022 McGlynn ND et al. JAMA Network Open.

eFigure 33. Network diagram for randomized controlled trials investigating the association of the substitution of LNCSBs for SSBs, water for SSBs, and LNCSBs for water with 2h-PP

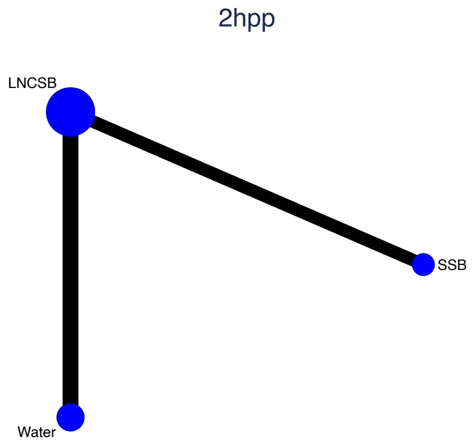

Blue nodes represent the study size for each beverage under investigation. The thickness of the black lines represents the number of studies directly comparing one beverage to another. 2h-PP, two-hour post prandial glucose; LNCSBs, non-nutritive sweetened beverages; SSBs, sugar-sweetened beverages.

© 2022 McGlynn ND et al. JAMA Network Open.

eFigure 34. Network diagram for randomized controlled trials investigating the association of the substitution of LNCSBs for SSBs, water for SSBs, and LNCSBs for water with FPI

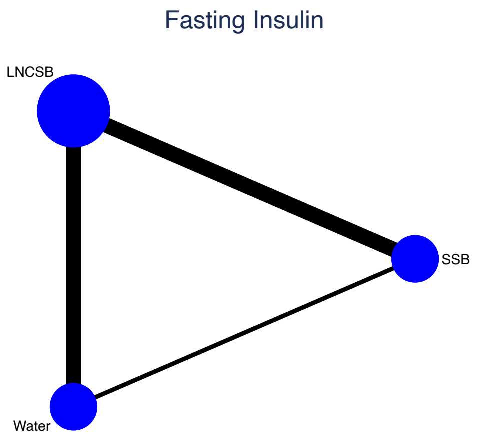

Blue nodes represent the study size for each beverage under investigation. The thickness of the black lines represents the number of studies directly comparing one beverage to another. 2h-PP, two-hour post prandial glucose; LNCSBs, non-nutritive sweetened beverages; SSBs, sugar-sweetened beverages.

© 2022 McGlynn ND et al. JAMA Network Open.

eFigure 35. Network diagram for randomized controlled trials investigating the association of the substitution of LNCSBs for SSBs, water for SSBs, and LNCSBs for water with HOMA-IR

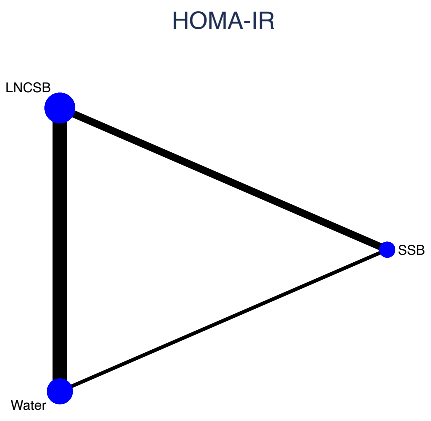

Blue nodes represent the study size for each beverage under investigation. The thickness of the black lines represents the number of studies directly comparing one beverage to another. HOMA-IR; homeostatic model assessment of insulin resistance; LNCSBs, non-nutritive sweetened beverages; SSBs, sugar-sweetened beverages.

© 2022 McGlynn ND et al. JAMA Network Open.

eFigure 36. Network diagram for randomized controlled trials investigating the association of the substitution of LNCSBs for SSBs, water for SSBs, and LNCSBs for water with LDL-C

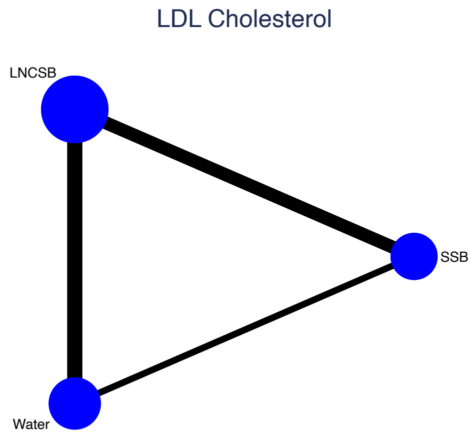

Blue nodes represent the study size for each beverage under investigation. The thickness of the black lines represents the number of studies directly comparing one beverage to another. LDL-C, low-density lipoprotein cholesterol; LNCSBs, non-nutritive sweetened beverages; SSBs, sugar-sweetened beverages.

© 2022 McGlynn ND et al. JAMA Network Open.

eFigure 37. Network diagram for randomized controlled trials investigating the association of the substitution of LNCSBs for SSBs, water for SSBs, and LNCSBs for water with Non-HDL-C

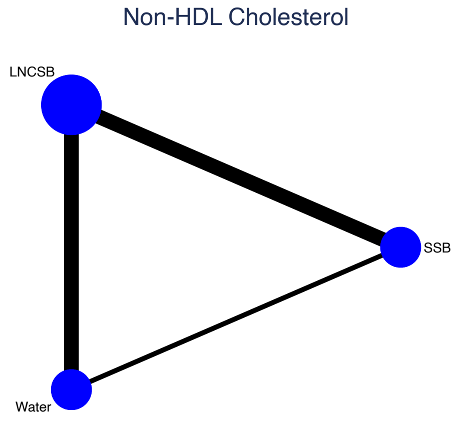

Blue nodes represent the study size for each beverage under investigation. The thickness of the black lines represents the number of studies directly comparing one beverage to another. Non-HDL-C, non-high-density lipoprotein cholesterol, LNCSBs, non-nutritive sweetened beverages; SSBs, sugar-sweetened beverages.

© 2022 McGlynn ND et al. JAMA Network Open.

eFigure 38. Network diagram for randomized controlled trials investigating the association of the substitution of LNCSBs for SSBs, water for SSBs, and LNCSBs for water with TGs

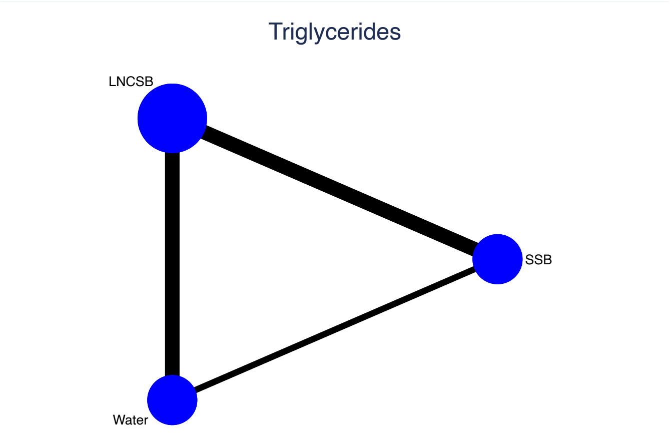

Blue nodes represent the study size for each beverage under investigation. The thickness of the black lines represents the number of studies directly comparing one beverage to another. LNCSBs, non-nutritive sweetened beverages; SSBs, sugar-sweetened beverages; TGs, triglycerides.

© 2022 McGlynn ND et al. JAMA Network Open.

eFigure 39. Network diagram for randomized controlled trials investigating the association of the substitution of LNCSBs for SSBs, water for SSBs, and LNCSBs for water with HDL-C

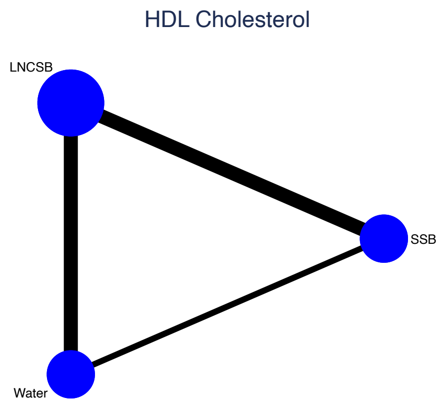

Blue nodes represent the study size for each beverage under investigation. The thickness of the black lines represents the number of studies directly comparing one beverage to another. HDL-C, high-density lipoprotein cholesterol, LNCSBs, non-nutritive sweetened beverages; SSBs, sugar-sweetened beverages.

© 2022 McGlynn ND et al. JAMA Network Open.

eFigure 40. Network diagram for randomized controlled trials investigating the association of the substitution of LNCSBs for SSBs, water for SSBs, and LNCSBs for water with TC

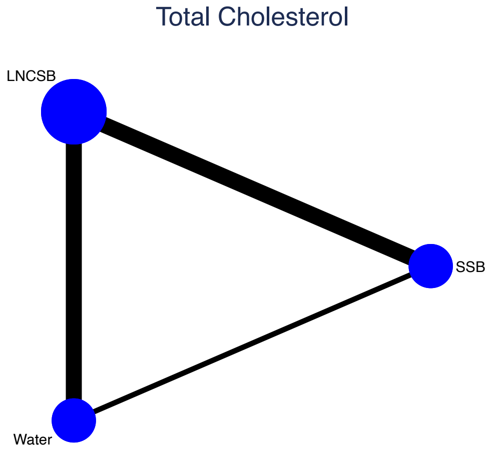

Blue nodes represent the study size for each beverage under investigation. The thickness of the black lines represents the number of studies directly comparing one beverage to another. LNCSBs, non-nutritive sweetened beverages; SSBs, sugar-sweetened beverages; TC, total cholesterol.

© 2022 McGlynn ND et al. JAMA Network Open.

eFigure 41. Network diagram for randomized controlled trials investigating the association of the substitution of LNCSBs for SSBs, water for SSBs, and LNCSBs for water with SBP

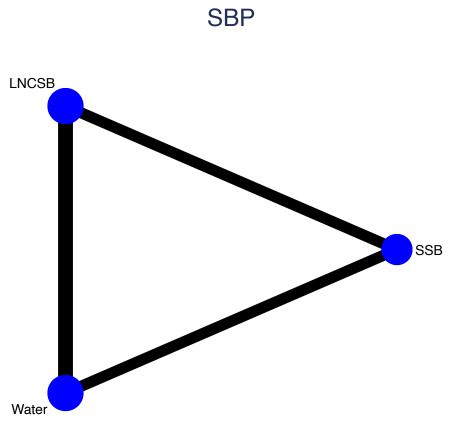

Blue nodes represent the study size for each beverage under investigation. The thickness of the black lines represents the number of studies directly comparing one beverage to another. LNCSBs, non-nutritive sweetened beverages; SBP, systolic blood pressure; SSBs, sugar-sweetened beverages.

© 2022 McGlynn ND et al. JAMA Network Open.

eFigure 42. Network diagram for randomized controlled trials investigating the association of the substitution of LNCSBs for SSBs, water for SSBs, and LNCSBs for water with DBP

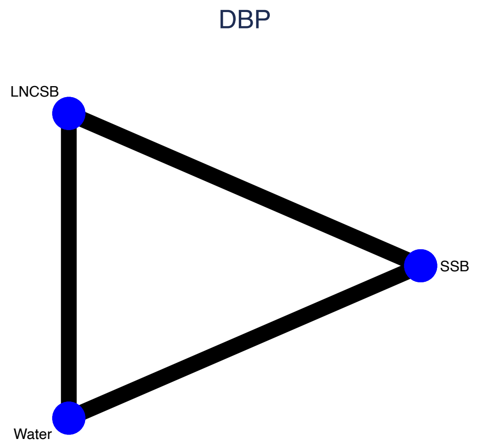

Blue nodes represent the study size for each beverage under investigation. The thickness of the black lines represents the number of studies directly comparing one beverage to another. DBP, diastolic blood pressure; LNCSBs, non-nutritive sweetened beverages; SSBs, sugar-sweetened beverages.

© 2022 McGlynn ND et al. JAMA Network Open.

eFigure 43. Network diagram for randomized controlled trials investigating the association of the substitution of LNCSBs for SSBs, water for SSBs, and LNCSBs for water with IHCL

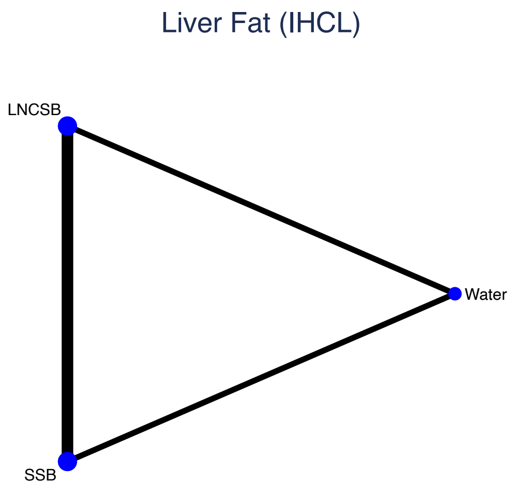

Blue nodes represent the study size for each beverage under investigation. The thickness of the black lines represents the number of studies directly comparing one beverage to another. IHCL, intra-hepatocellular lipid; LNCSBs, non-nutritive sweetened beverages; SSBs, sugar-sweetened beverages.

© 2022 McGlynn ND et al. JAMA Network Open.

eFigure 44. Network diagram for randomized controlled trials investigating the association of the substitution of LNCSBs for SSBs, water for SSBs, and LNCSBs for water with ALT

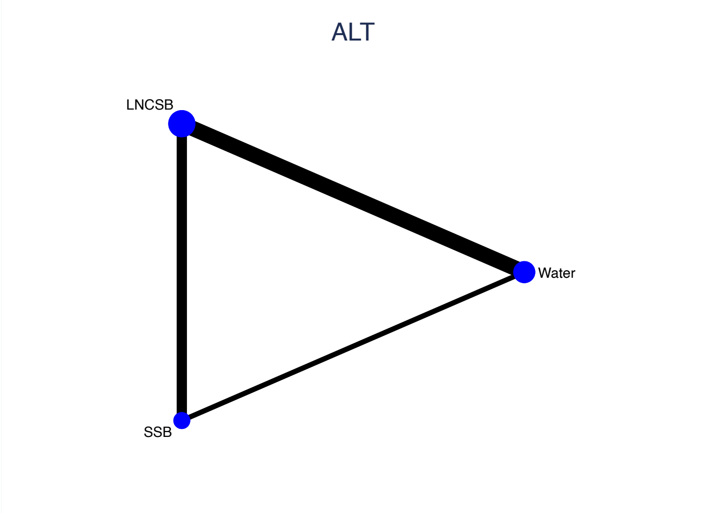

Blue nodes represent the study size for each beverage under investigation. The thickness of the black lines represents the number of studies directly comparing one beverage to another. ALT, alanine transaminase; LNCSBs, non-nutritive sweetened beverages; SSBs, sugar-sweetened beverages.

© 2022 McGlynn ND et al. JAMA Network Open.

eFigure 45. Network diagram for randomized controlled trials investigating the association of the substitution of LNCSBs for SSBs, water for SSBs, and LNCSBs for water with AST

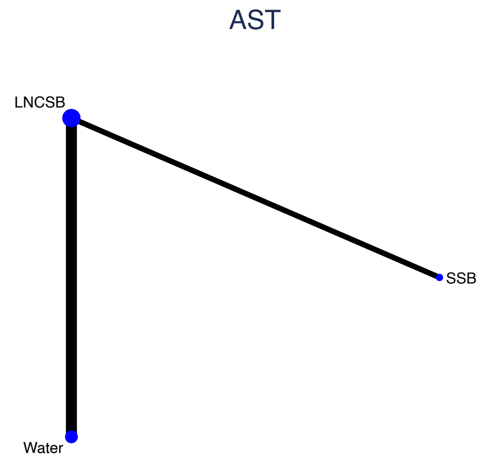

Blue nodes represent the study size for each beverage under investigation. The thickness of the black lines represents the number of studies directly comparing one beverage to another. AST, aspartate transaminase; LNCSBs, non-nutritive sweetened beverages; SSBs, sugar-sweetened beverages.

© 2022 McGlynn ND et al. JAMA Network Open.

eFigure 46. Network diagram for randomized controlled trials investigating the association of the substitution of LNCSBs for SSBs, water for SSBs, and LNCSBs for water with uric acid

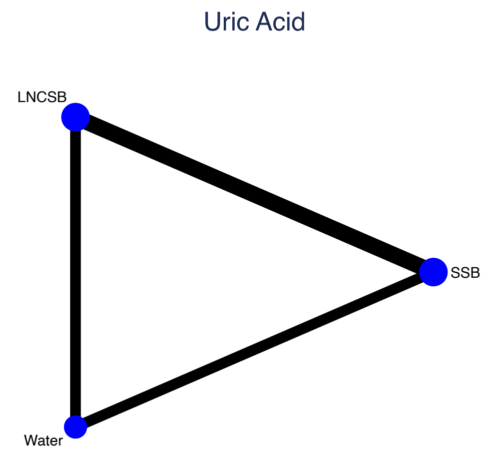

Blue nodes represent the study size for each beverage under investigation. The thickness of the black lines represents the number of studies directly comparing one beverage to another. LNCSBs, non-nutritive sweetened beverages; SSBs, sugar-sweetened beverages.

© 2022 McGlynn ND et al. JAMA Network Open.

Publication Bias

eFigure 47. Comparison adjusted funnel plot for Body Weight

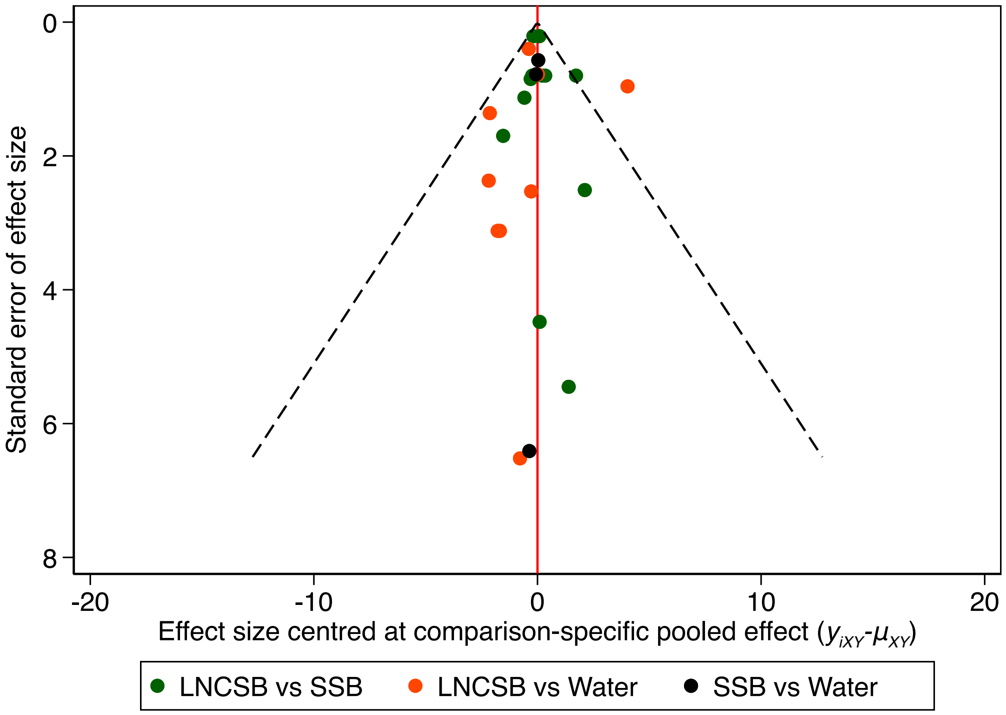

The horizontal axis represents an adjusted effect size, presenting the difference between each observed effect size and the mean effect size for the specific comparison being made. The dashed lines represent pseudo 95% confidence intervals.

© 2022 McGlynn ND et al. JAMA Network Open.

eFigure 48. Comparison adjusted funnel plot for BMI

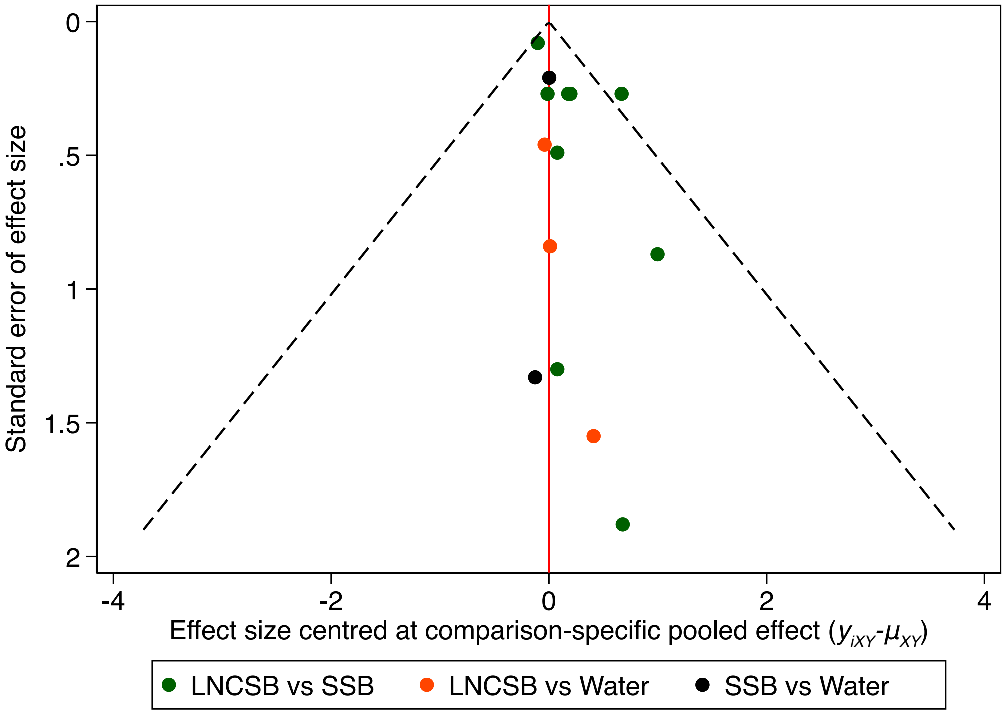

The horizontal axis represents an adjusted effect size, presenting the difference between each observed effect size and the mean effect size for the specific comparison being made. The dashed lines represent pseudo 95% confidence intervals.

© 2022 McGlynn ND et al. JAMA Network Open.

eFigure 49. Comparison adjusted funnel plot for Body Fat (%)

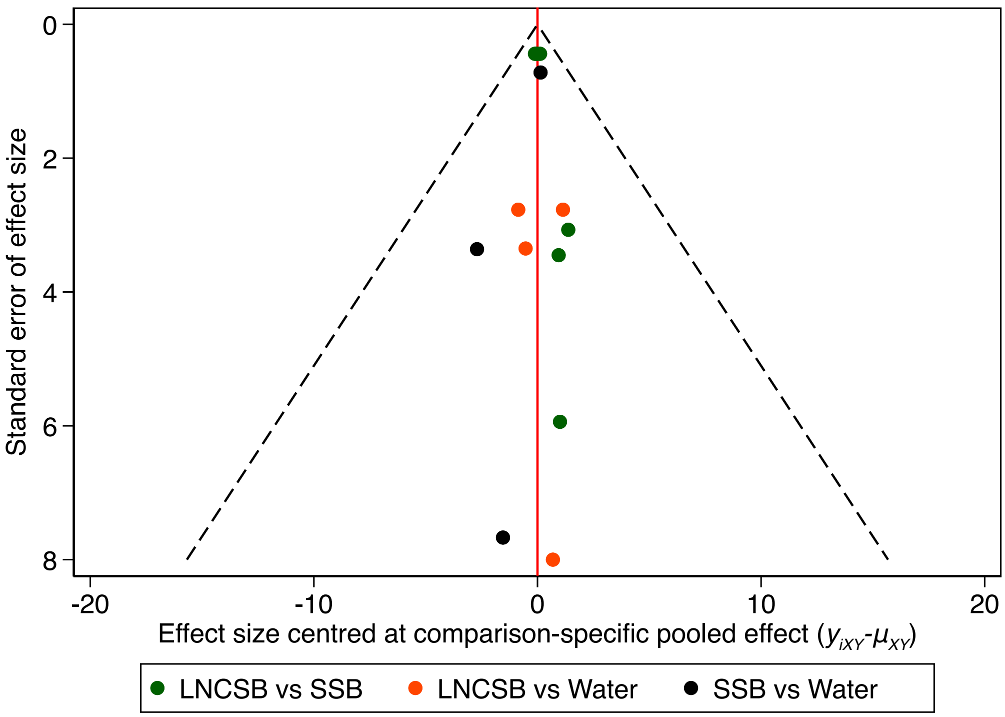

The horizontal axis represents an adjusted effect size, presenting the difference between each observed effect size and the mean effect size for the specific comparison being made. The dashed lines represent pseudo 95% confidence intervals.

© 2022 McGlynn ND et al. JAMA Network Open.

eFigure 50. Comparison adjusted funnel plot for Fasting Insulin

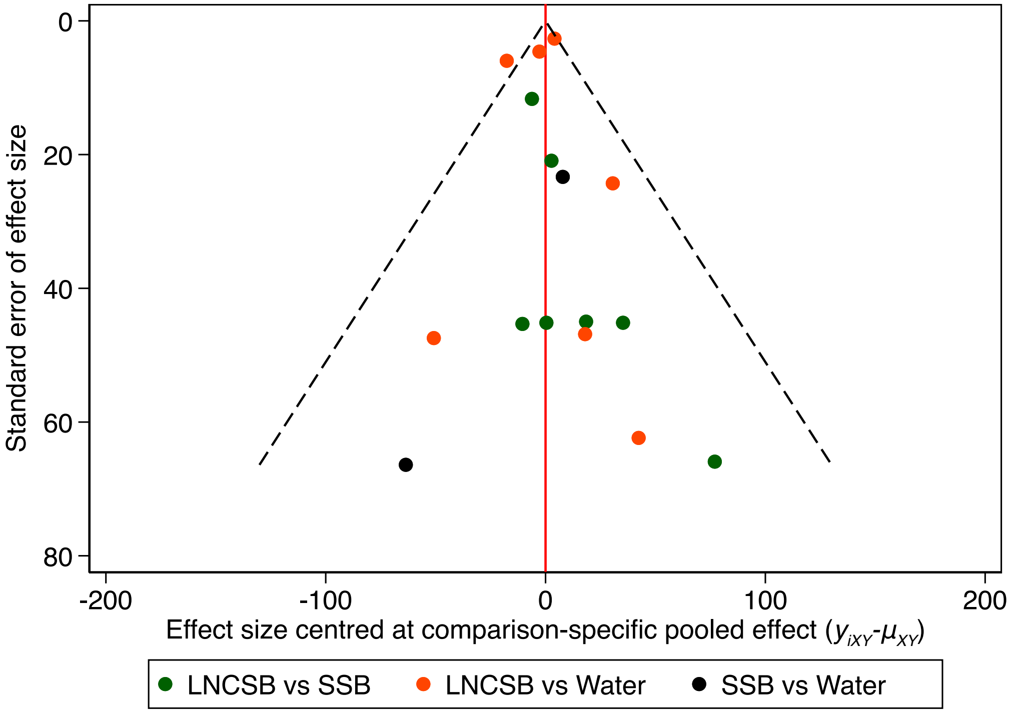

The horizontal axis represents an adjusted effect size, presenting the difference between each observed effect size and the mean effect size for the specific comparison being made. The dashed lines represent pseudo 95% confidence intervals.

© 2022 McGlynn ND et al. JAMA Network Open.

eFigure 51. Comparison adjusted funnel plot for Fasting Blood Glucose

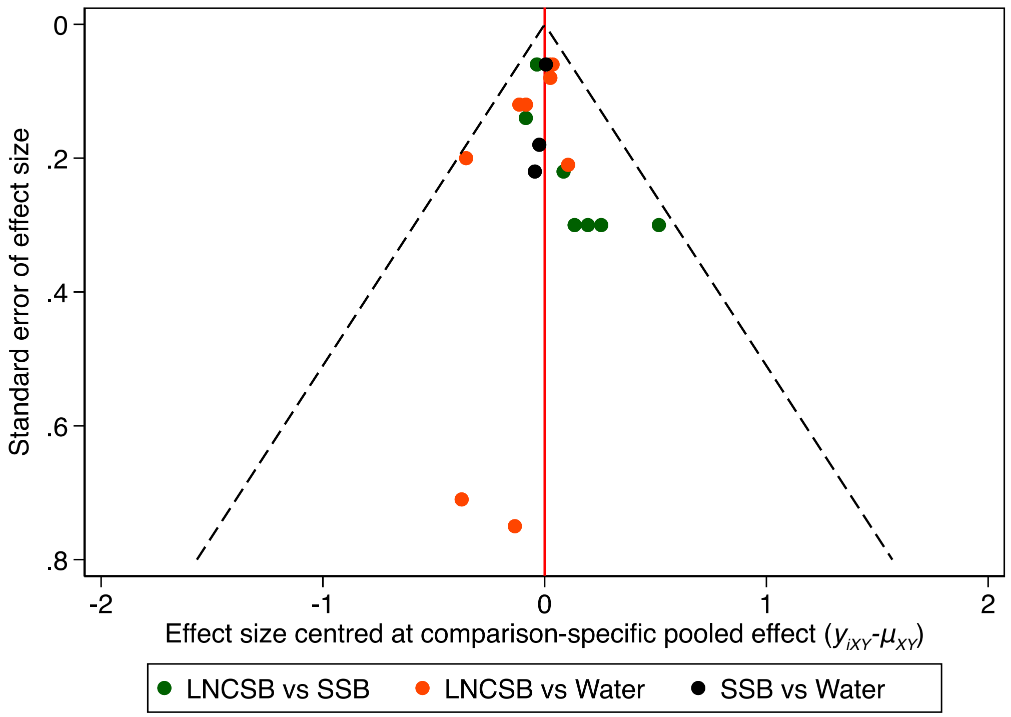

The horizontal axis represents an adjusted effect size, presenting the difference between each observed effect size and the mean effect size for the specific comparison being made. The dashed lines represent pseudo 95% confidence intervals.

© 2022 McGlynn ND et al. JAMA Network Open.

eFigure 52. Comparison adjusted funnel plot for LDL-Cholesterol

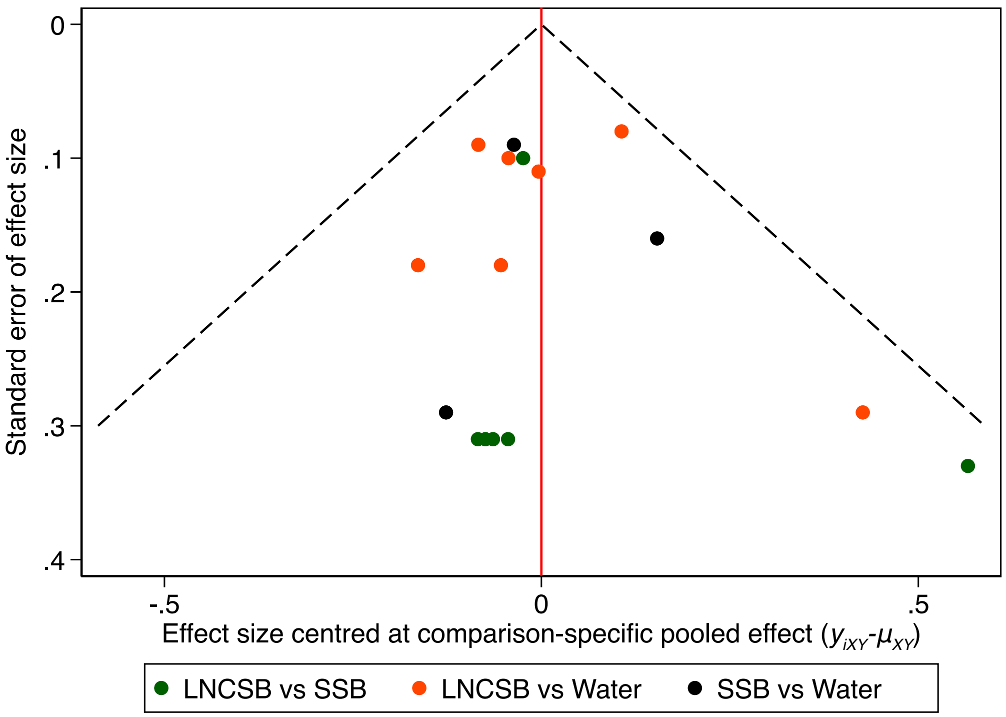

The horizontal axis represents an adjusted effect size, presenting the difference between each observed effect size and the mean effect size for the specific comparison being made. The dashed lines represent pseudo 95% confidence intervals.

© 2022 McGlynn ND et al. JAMA Network Open.

eFigure 53. Comparison adjusted funnel plot for Non-HDL-Cholesterol

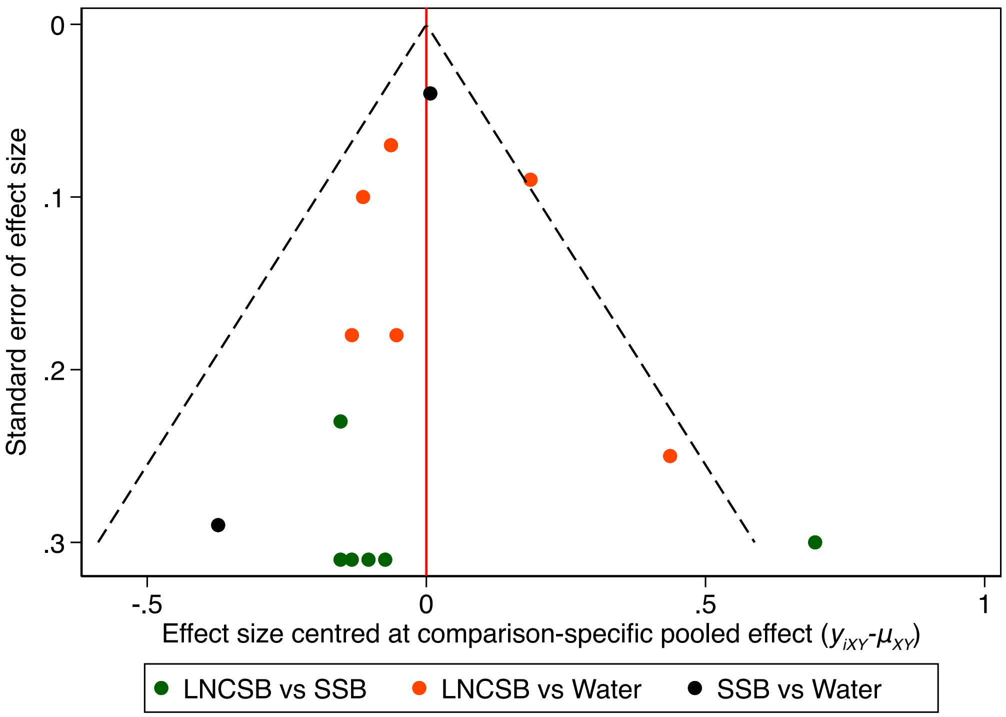

The horizontal axis represents an adjusted effect size, presenting the difference between each observed effect size and the mean effect size for the specific comparison being made. The dashed lines represent pseudo 95% confidence intervals.

© 2022 McGlynn ND et al. JAMA Network Open.

eFigure 54. Comparison adjusted funnel plot for Triglycerides

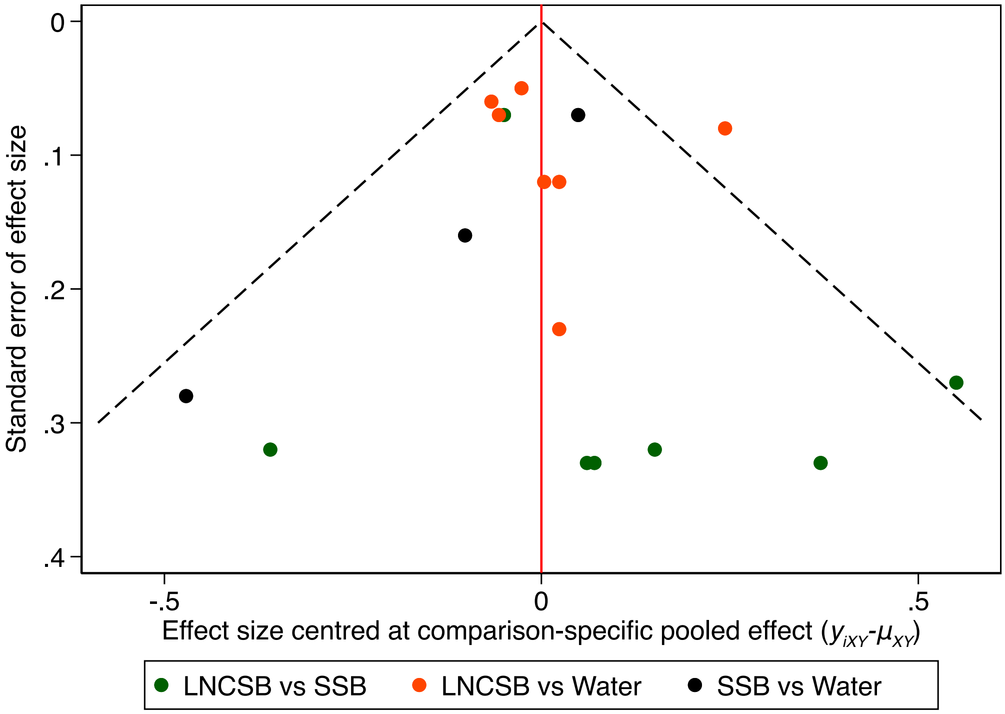

The horizontal axis represents an adjusted effect size, presenting the difference between each observed effect size and the mean effect size for the specific comparison being made. The dashed lines represent pseudo 95% confidence intervals.

© 2022 McGlynn ND et al. JAMA Network Open.

eFigure 55. Comparison adjusted funnel plot for HDL-Cholesterol

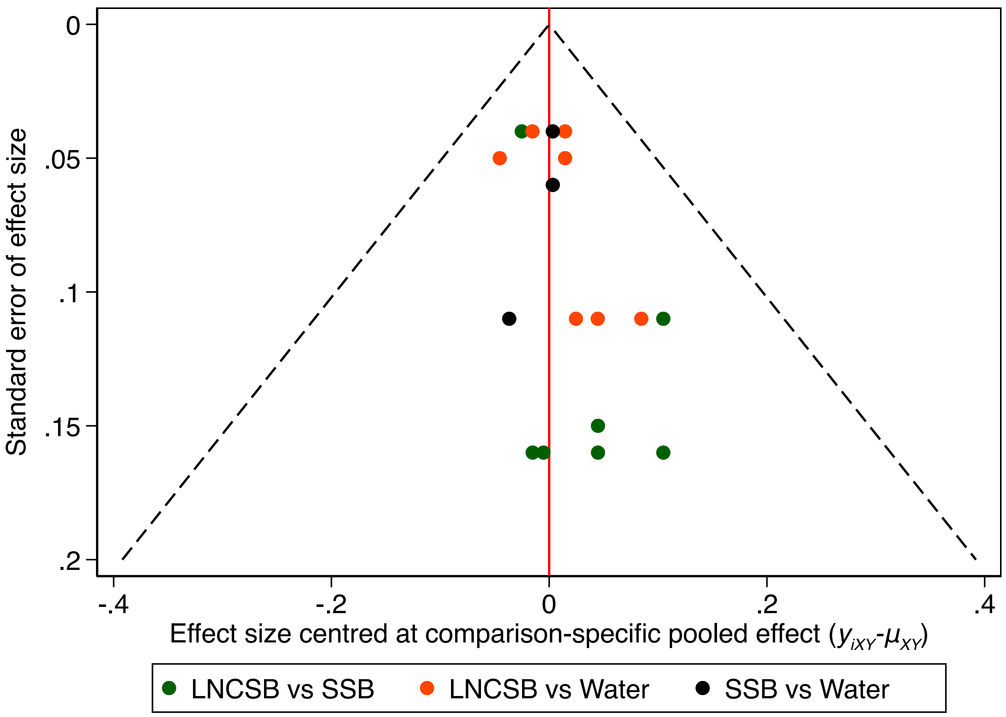

The horizontal axis represents an adjusted effect size, presenting the difference between each observed effect size and the mean effect size for the specific comparison being made. The dashed lines represent pseudo 95% confidence intervals.

© 2022 McGlynn ND et al. JAMA Network Open.

eFigure 56. Comparison adjusted funnel plot for Total-Cholesterol

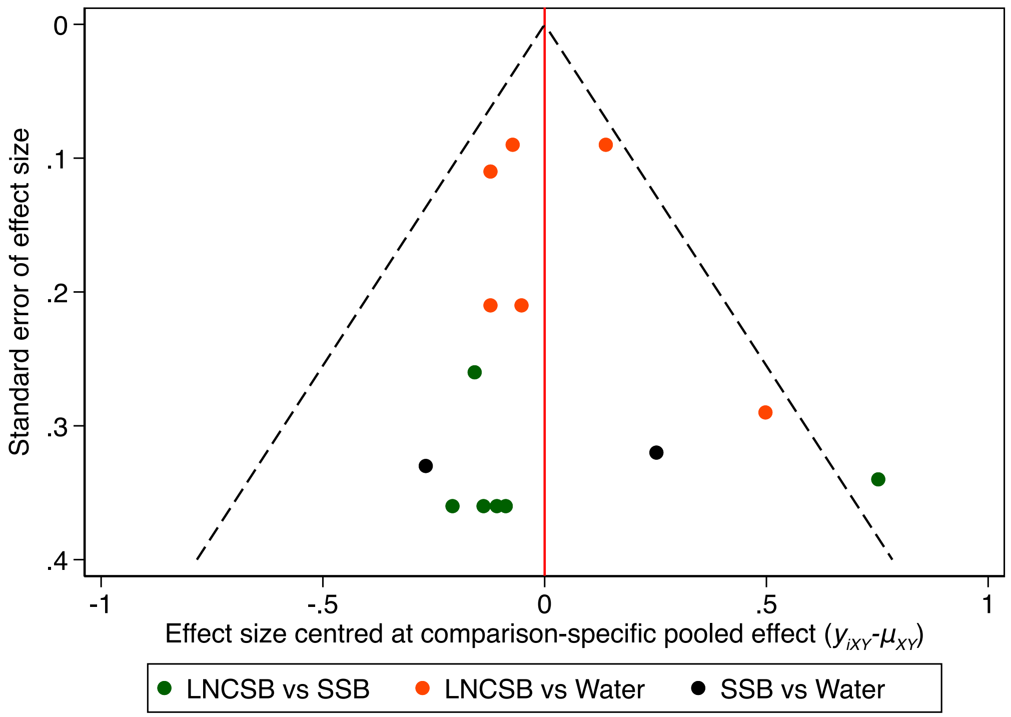

The horizontal axis represents an adjusted effect size, presenting the difference between each observed effect size and the mean effect size for the specific comparison being made. The dashed lines represent pseudo 95% confidence intervals.

© 2022 McGlynn ND et al. JAMA Network Open.

eFigure 57. Comparison adjusted funnel plot for Systolic Blood Pressure

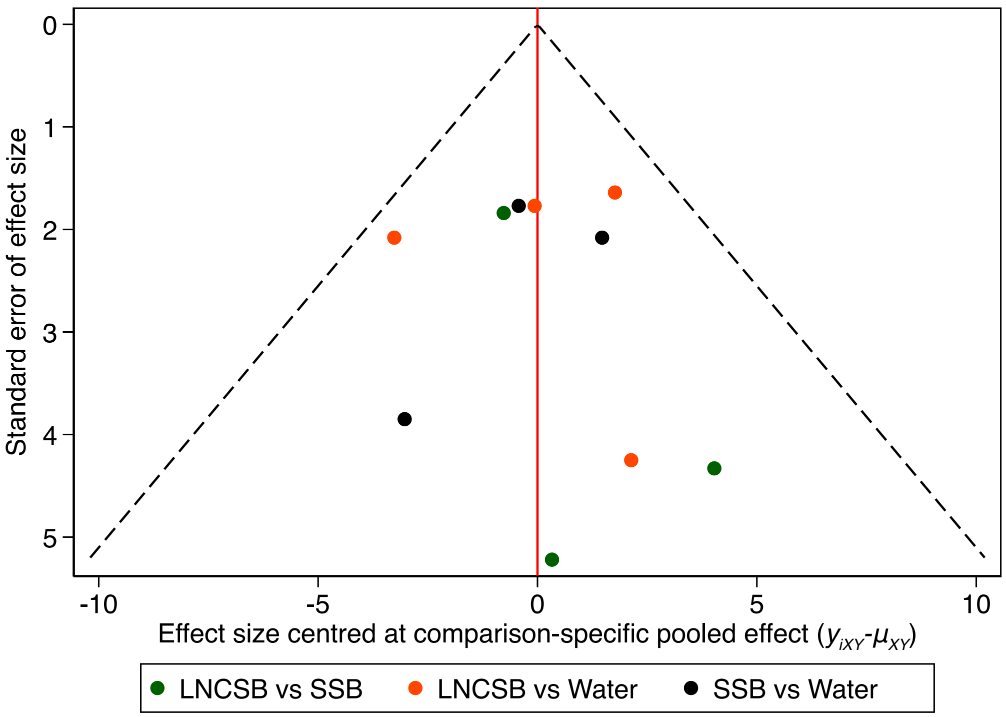

The horizontal axis represents an adjusted effect size, presenting the difference between each observed effect size and the mean effect size for the specific comparison being made. The dashed lines represent pseudo 95% confidence intervals.

© 2022 McGlynn ND et al. JAMA Network Open.

Mean Differences and Standard Errors

eData 1. Body Weight (Kg)

Author	Treatment 1	Treatment 2	Mean Difference	Standard Error
Bonnet 2018	Water	LNCSB	-0.59	0.4
Campos 2015	SSB	LNCSB	-2.3	5.45
Ebbeling 2020	Water	LNCSB	-1	0.78
Ebbeling 2020	SSB	LNCSB	-0.6	0.85
Ebbeling 2020	SSB	Water	-0.5	0.78
Engel 2018	Water	LNCSB	-0.2	6.52
Engel 2018	SSB	LNCSB	-1	4.48
Engel 2018	SSB	Water	-0.80	6.41
Hernandez-Cordero 2014	SSB	Water	-0.4	0.57
Higgins 2018 (1050mg ASP)	Water	LNCSB	0.7	3.12
Higgins 2018 (350mg ASP)	Water	LNCSB	0.8	3.12
Higgins 2019 (Asp)	SSB	LNCSB	-1.12	0.8
Higgins 2019 (RebA)	SSB	LNCSB	-1.25	0.8
Higgins 2019 (Sacrn)	SSB	LNCSB	-0.67	0.8
Higgins 2019 (Sucralose)	SSB	LNCSB	-2.63	0.8
Madjd 2015	Water	LNCSB	1.20	2.37
Madjd 2017	Water	LNCSB	1.15	1.36
Peters 2016	Water	LNCSB	-5.01	0.96
Reid 2007	SSB	LNCSB	0.63	1.7
Reid 2010	SSB	LNCSB	-0.32	1.13
Reid 2014	SSB	LNCSB	-3.02	2.51
Tate 2012	Water	LNCSB	-0.70	2.53
Tordoff 1990 (Females)	SSB	LNCSB	-0.72	0.21
Tordoff 1990 (Males)	SSB	LNCSB	-0.99	0.21

© 2022 McGlynn ND et al. JAMA Network Open.

eData 2. BMI (kg/m^2)

Author	Treatment 1	Treatment 2	Mean Difference	Standard Error
Campos 2015	SSB	LNCSB	-0.9	1.88
Engel 2018	Water	LNCSB	0	1.55
Engel 2018	SSB	LNCSB	-0.3	1.30
Engel 2018	SSB	Water	-0.3	1.33
Hernandez-Cordero 2014	SSB	Water	-0.17	0.21
Higgins 2019 (Asp)	SSB	LNCSB	-0.4	0.27
Higgins 2019 (RebA)	SSB	LNCSB	-0.42	0.27
Madjd 2015	Water	LNCSB	0.40	0.84
Madjd 2017	Water	LNCSB	0.45	0.46
Reid 2007	SSB	LNCSB	-0.3	0.49
Reid 2010	SSB	LNCSB	-0.12	0.08
Reid 2014	SSB	LNCSB	-1.22	0.87

© 2022 McGlynn ND et al. JAMA Network Open.

eData 3. Body Fat (%)

Author	Treatment 1	Treatment 2	Mean Difference	Standard Error
Campos 2015	SSB	LNCSB	-2	3.07
Ebbeling 2020	Water	LNCSB	1.27	3.35
Ebbeling 2020	SSB	LNCSB	-1.57	3.45
Ebbeling 2020	SSB	Water	-2.84	3.36
Engel 2018	Water	LNCSB	0.05	8.00
Engel 2018	SSB	LNCSB	-1.63	5.94
Engel 2018	SSB	Water	-1.68	7.67
Hernandez-Cordero 2014	SSB	Water	0	0.72
Higgins 2018 (1050mg ASP)	Water	LNCSB	-0.4	2.77
Higgins 2018 (350mg ASP)	Water	LNCSB	1.6	2.77
Higgins 2019 (Asp)	SSB	LNCSB	-0.51	0.44
Higgins 2019 (RebA)	SSB	LNCSB	-0.61	0.44
Higgins 2019 (Sacrn)	SSB	LNCSB	-0.58	0.44
Higgins 2019 (Sucralose)	SSB	LNCSB	-0.74	0.44

© 2022 McGlynn ND et al. JAMA Network Open.

eData 4. Waist Circumference (cm)

Author	Treatment 1	Treatment 2	Mean Difference	Standard Error
Bonnet 2018	Water	LNCSB	0.2	0.66
Hernandez-Cordero 2014	SSB	Water	0.3	0.81
Madjd 2015	Water	LNCSB	0.7	1.56
Madjd 2017	Water	LNCSB	0	1.42
Peters 2016	Water	LNCSB	-4.5	1.16
Tate 2012	Water	LNCSB	-0.1	2.01

© 2022 McGlynn ND et al. JAMA Network Open.

eData 5. HbA1c (%)

Author	Treatment 1	Treatment 2	Mean Difference	Standard Error
Hernandez-Cordero 2014	SSB	Water	-0.05	0.04
Higgins 2018 (1050mg ASP)	Water	LNCSB	0.01	0.07
Higgins 2018 (350mg ASP)	Water	LNCSB	0.03	0.07
Higgins 2019 (Asp)	SSB	LNCSB	0.09	0.13
Higgins 2019 (RebA)	SSB	LNCSB	0.12	0.13
Higgins 2019 (Sacrn)	SSB	LNCSB	0.14	0.13
Madjd 2015	Water	LNCSB	0.20	0.11
Madjd 2017	Water	LNCSB	0.75	0.13

© 2022 McGlynn ND et al. JAMA Network Open.

eData 6. Fasting Blood Glucose (mmol/L)

Author	Treatment 1	Treatment 2	Mean Difference	Standard Error
Bonnet 2018	Water	LNCSB	-0.04	0.06
Campos 2015	SSB	LNCSB	0.00	0.14
Ebbeling 2020	Water	LNCSB	-0.05	0.06
Ebbeling 2020	SSB	LNCSB	-0.05	0.06
Ebbeling 2020	SSB	Water	0	0.06
Engel 2018	Water	LNCSB	-0.12	0.21
Engel 2018	SSB	LNCSB	-0.17	0.22
Engel 2018	SSB	Water	-0.05	0.22
Hernandez-Cordero 2014	SSB	Water	-0.03	0.18
Higgins 2018 (1050mg ASP)	Water	LNCSB	0.12	0.75
Higgins 2018 (350mg ASP)	Water	LNCSB	0.36	0.71
Higgins 2019 (Asp)	SSB	LNCSB	-0.34	0.3
Higgins 2019 (RebA)	SSB	LNCSB	-0.22	0.3
Higgins 2019 (Sacrn)	SSB	LNCSB	-0.60	0.3
Higgins 2019 (Sucralose)	SSB	LNCSB	-0.28	0.3
Madjd 2015	Water	LNCSB	0.10	0.12
Madjd 2017	Water	LNCSB	0.34	0.2
Peters 2016	Water	LNCSB	-0.04	0.08
Tate 2012	Water	LNCSB	0.07	0.12

© 2022 McGlynn ND et al. JAMA Network Open.

eData 7. 2-Hour Post-prandial Glucose (mmol/L)

Author	Treatment 1	Treatment 2	Mean Difference	Standard Error
Bonnet 2018	Water	LNCSB	0.12	0.16
Higgins 2018 (1050mg ASP)	Water	LNCSB	-0.18	0.61
Higgins 2018 (350mg ASP)	Water	LNCSB	-0.64	0.56
Higgins 2019 (Asp)	SSB	LNCSB	0.1	0.75
Higgins 2019 (RebA)	SSB	LNCSB	0.39	0.76
Higgins 2019 (Sacrn)	SSB	LNCSB	0.26	0.76
Higgins 2019 (Sucralose)	SSB	LNCSB	0.42	0.76
Madjd 2015	Water	LNCSB	0.30	0.16
Madjd 2017	Water	LNCSB	0.31	0.24

© 2022 McGlynn ND et al. JAMA Network Open.

eData 8. Fasting Plasma Insulin (pmol/L)

Author	Treatment 1	Treatment 2	Mean Difference	Standard Error
Bonnet 2018	Water	LNCSB	0.78	2.66
Campos 2015	SSB	LNCSB	-15.28	20.92
Ebbeling 2020	Water	LNCSB	-37.5	62.37
Ebbeling 2020	SSB	LNCSB	-89.59	65.92
Ebbeling 2020	SSB	Water	-52.09	66.39
Engel 2018	Water	LNCSB	-25.72	24.30
Engel 2018	SSB	LNCSB	-6.4	11.67
Engel 2018	SSB	Water	19.32	23.33
Higgins 2018 (1050mg ASP)	Water	LNCSB	-13.13	46.85
Higgins 2018 (350mg ASP)	Water	LNCSB	55.69	47.44
Higgins 2019 (Asp)	SSB	LNCSB	-31.04	44.98
Higgins 2019 (RebA)	SSB	LNCSB	-12.99	45.15
Higgins 2019 (Sacrn)	SSB	LNCSB	-47.85	45.15
Higgins 2019 (Sucralose)	SSB	LNCSB	-2.08	45.32
Madjd 2015	Water	LNCSB	7.64	4.6
Madjd 2017	Water	LNCSB	22.50	5.97

© 2022 McGlynn ND et al. JAMA Network Open.

eData 9. HOMA-IR

Author	Treatment 1	Treatment 2	Mean Difference	Standard Error
Bonnet 2018	Water	LNCSB	-0.19	0.10
Campos 2015	SSB	LNCSB	-0.80	0.82
Engel 2018	Water	LNCSB	-0.44	0.44
Engel 2018	SSB	LNCSB	-0.13	0.23
Engel 2018	SSB	Water	0.31	0.42
Madjd 2015	Water	LNCSB	0.20	0.18
Madjd 2017	Water	LNCSB	0.71	0.38

© 2022 McGlynn ND et al. JAMA Network Open.

eData 10. LDL-Cholesterol (mmol/L)

Author	Treatment 1	Treatment 2	Mean Difference	Standard Error
Ebbeling 2020	Water	LNCSB	0.08	0.09
Ebbeling 2020	SSB	LNCSB	0.01	0.1
Ebbeling 2020	SSB	Water	-0.06	0.09
Engel 2018	Water	LNCSB	-0.43	0.29
Engel 2018	SSB	LNCSB	-0.58	0.33
Engel 2018	SSB	Water	-0.15	0.29
Hernandez-Cordero 2014	SSB	Water	0.13	0.16
Higgins 2018 (1050mg ASP)	Water	LNCSB	0.16	0.18
Higgins 2018 (350mg ASP)	Water	LNCSB	0.05	0.18
Higgins 2019 (Asp)	SSB	LNCSB	0.05	0.31
Higgins 2019 (RebA)	SSB	LNCSB	0.06	0.31
Higgins 2019 (Sacrn)	SSB	LNCSB	0.07	0.31
Higgins 2019 (Sucralose)	SSB	LNCSB	0.03	0.31
Madjd 2015	Water	LNCSB	0.00	0.11
Madjd 2017	Water	LNCSB	0.04	0.1
Peters 2016	Water	LNCSB	-0.11	0.08

© 2022 McGlynn ND et al. JAMA Network Open.

eData 11. Non-HDL-Cholesterol (mmol/L)

Author	Treatment 1	Treatment 2	Mean Difference	Standard Error
Campos 2015	SSB	LNCSB	0.00	0.23
Engel 2018	Water	LNCSB	-0.45	0.25
Engel 2018	SSB	LNCSB	-0.85	0.3
Engel 2018	SSB	Water	-0.40	0.29
Hernandez-Cordero 2014	SSB	Water	-0.02	0.04
Higgins 2018 (1050mg ASP)	Water	LNCSB	0.12	0.18
Higgins 2018 (350mg ASP)	Water	LNCSB	0.04	0.18
Higgins 2019 (Asp)	SSB	LNCSB	-0.05	0.31
Higgins 2019 (RebA)	SSB	LNCSB	0	0.31
Higgins 2019 (Sacrn)	SSB	LNCSB	-0.08	0.31
Higgins 2019 (Sucralose)	SSB	LNCSB	-0.02	0.31
Madjd 2015	Water	LNCSB	0.10	0.10
Madjd 2017	Water	LNCSB	0.05	0.07
Peters 2016	Water	LNCSB	-0.20	0.09

© 2022 McGlynn ND et al. JAMA Network Open.

eData 12. Triglycerides (mmol/L)

Author	Treatment 1	Treatment 2	Mean Difference	Standard Error
Campos 2015	SSB	LNCSB	0.30	0.32
Ebbeling 2020	Water	LNCSB	0.03	0.07
Ebbeling 2020	SSB	LNCSB	-0.01	0.07
Ebbeling 2020	SSB	Water	-0.04	0.07
Engel 2018	Water	LNCSB	-0.05	0.23
Engel 2018	SSB	LNCSB	-0.61	0.27
Engel 2018	SSB	Water	-0.56	0.28
Hernandez-Cordero 2014	SSB	Water	-0.19	0.16
Higgins 2018 (1050mg ASP)	Water	LNCSB	-0.05	0.12
Higgins 2018 (350mg ASP)	Water	LNCSB	-0.03	0.12
Higgins 2019 (Asp)	SSB	LNCSB	-0.21	0.32
Higgins 2019 (RebA)	SSB	LNCSB	-0.13	0.33
Higgins 2019 (Sacrn)	SSB	LNCSB	-0.43	0.33
Higgins 2019 (Sucralose)	SSB	LNCSB	-0.12	0.33
Madjd 2015	Water	LNCSB	0.00	0.05
Madjd 2017	Water	LNCSB	0.04	0.06
Peters 2016	Water	LNCSB	-0.27	0.08

© 2022 McGlynn ND et al. JAMA Network Open.

eData 13. HDL-Cholesterol (mmol/L)

Author	Treatment 1	Treatment 2	Mean Difference	Standard Error
Campos 2015	SSB	LNCSB	0.00	0.15
Ebbeling 2020	Water	LNCSB	0.03	0.04
Ebbeling 2020	SSB	LNCSB	0.07	0.04
Ebbeling 2020	SSB	Water	0.05	0.04
Engel 2018	Water	LNCSB	-0.07	0.11
Engel 2018	SSB	LNCSB	-0.06	0.11
Engel 2018	SSB	Water	0.01	0.11
Hernandez-Cordero 2014	SSB	Water	0.05	0.06
Higgins 2018 (1050mg ASP)	Water	LNCSB	-0.03	0.11
Higgins 2018 (350mg ASP)	Water	LNCSB	-0.01	0.11
Higgins 2019 (Asp)	SSB	LNCSB	0	0.16
Higgins 2019 (RebA)	SSB	LNCSB	0.05	0.16
Higgins 2019 (Sacrn)	SSB	LNCSB	0.06	0.16
Higgins 2019 (Sucralose)	SSB	LNCSB	-0.06	0.16
Madjd 2015	Water	LNCSB	0.00	0.05
Madjd 2017	Water	LNCSB	0.00	0.04
Peters 2016	Water	LNCSB	0.06	0.05

© 2022 McGlynn ND et al. JAMA Network Open.

eData 14. Total-Cholesterol (mmol/L)

Author	Treatment 1	Treatment 2	Mean Difference	Standard Error
Campos 2015	SSB	LNCSB	0	0.26
Engel 2018	Water	LNCSB	-0.52	0.29
Engel 2018	SSB	LNCSB	-0.91	0.34
Engel 2018	SSB	Water	-0.39	0.33
Hernandez-Cordero 2014	SSB	Water	0.13	0.32
Higgins 2018 (1050mg ASP)	Water	LNCSB	0.1	0.21
Higgins 2018 (350mg ASP)	Water	LNCSB	0.03	0.21
Higgins 2019 (Asp)	SSB	LNCSB	-0.05	0.36
Higgins 2019 (RebA)	SSB	LNCSB	0.05	0.36
Higgins 2019 (Sacrn)	SSB	LNCSB	-0.02	0.36
Higgins 2019 (Sucralose)	SSB	LNCSB	-0.07	0.36
Madjd 2015	Water	LNCSB	0.10	0.11
Madjd 2017	Water	LNCSB	0.05	0.09
Peters 2016	Water	LNCSB	-0.16	0.09

© 2022 McGlynn ND et al. JAMA Network Open.

eData 15. Systolic Blood Pressure (mmHg)

Author	Treatment 1	Treatment 2	Mean Difference	Standard Error
Campos 2015	SSB	LNCSB	-3.50	5.22
Ebbeling 2020	Water	LNCSB	-2.4	1.77
Ebbeling 2020	SSB	LNCSB	-2.4	1.84
Ebbeling 2020	SSB	Water	0	1.77
Engel 2018	Water	LNCSB	-4.6	4.25
Engel 2018	SSB	LNCSB	-7.2	4.33
Engel 2018	SSB	Water	-2.60	3.85
Hernandez-Cordero 2014	SSB	Water	1.9	2.08
Peters 2016	Water	LNCSB	-4.23	1.64
Tate 2012	Water	LNCSB	0.80	2.08

© 2022 McGlynn ND et al. JAMA Network Open.

eData 16. Diastolic Blood Pressure (mmHg)

Author	Treatment 1	Treatment 2	Mean Difference	Standard Error
Campos 2015	SSB	LNCSB	-3.20	3.49
Ebbeling 2020	Water	LNCSB	0.8	1.49
Ebbeling 2020	SSB	LNCSB	-1.5	1.56
Ebbeling 2020	SSB	Water	-2.3	1.49
Engel 2018	Water	LNCSB	-4.4	3.21
Engel 2018	SSB	LNCSB	-6.9	3.33
Engel 2018	SSB	Water	-2.50	3.34
Hernandez-Cordero 2014	SSB	Water	0.5	1.86
Tate 2012	Water	LNCSB	0.30	1.43

© 2022 McGlynn ND et al. JAMA Network Open.

eData 17. Intrahepatocellular Lipid/Liver fat (SMD)

Author	Treatment 1	Treatment 2	Mean Difference	Standard Error
Campos 2015	SSB	LNCSB	-0.38	0.19
Maersk 2012	Water	LNCSB	-0.08	0.20
Maersk 2012	SSB	LNCSB	-0.47	0.21
Maersk 2012	SSB	Water	-0.46	0.21

© 2022 McGlynn ND et al. JAMA Network Open.

eData 18. Alanine Aminotransferase (U/L)

Author	Treatment 1	Treatment 2	Mean Difference	Standard Error
Campos 2015	SSB	LNCSB	-3.50	7.1
Ebbeling 2020	Water	LNCSB	-8.80	7.07
Ebbeling 2020	SSB	LNCSB	-13.90	7.44
Ebbeling 2020	SSB	Water	-5.10	7.57
Higgins 2018 (1050mg ASP)	Water	LNCSB	2.5	2.59
Higgins 2018 (350mg ASP)	Water	LNCSB	-0.4	2.55

© 2022 McGlynn ND et al. JAMA Network Open.

eData 19. Aspartate Aminotransferase (U/L)

Author	Treatment 1	Treatment 2	Mean Difference	Standard Error
Campos 2015	SSB	LNCSB	-1.50	3.25
Higgins 2018 (1050mg ASP)	Water	LNCSB	-0.30	3.06
Higgins 2018 (350mg ASP)	Water	LNCSB	0.70	3.06

© 2022 McGlynn ND et al. JAMA Network Open.

eData S20: Uric Acid (mmol/L)

Author	Treatment 1	Treatment 2	Mean Difference	Standard Error
Bruun 2015	Water	LNCSB	0.01	0.03
Bruun 2015	SSB	LNCSB	-0.04	0.02
Bruun 2015	SSB	Water	-0.05	0.02
Campos 2015	SSB	LNCSB	0.00	0.03
Ebbeling 2020	Water	LNCSB	-0.01	0.01
Ebbeling 2020	SSB	LNCSB	0.00	0.01
Ebbeling 2020	SSB	Water	0.01	0.01

© 2022 McGlynn ND et al. JAMA Network Open.

References

1. Hutton B, Salanti G, Caldwell DM, et al. The PRISMA extension statement for reporting of systematic reviews incorporating network meta-analyses of health care interventions: Checklist and explanations. *Annals of Internal Medicine* 2015; **162**(11): 777.
2. Higgins JPT, Altman DG, Gotzsche PC, et al. The Cochrane Collaboration's tool for assessing risk of bias in randomised trials. *BMJ* 2011; **343**(oct18 2): d5928-d.
3. Higgins J, Thomas, J. Cochrane handbook for systematic reviews of interventions. Ed 6. 2019. https://training.cochrane.org/handbook/current.
4. Luo D, Wan X, Liu J, Tong T. Optimally estimating the sample mean from the sample size, median, mid-range, and/or mid-quartile range. *Statistical methods in medical research* 2018; **27**(6): 1785-805.
5. Wan X, Wang W, Liu J, Tong T. Estimating the sample mean and standard deviation from the sample size, median, range and/or interquartile range. *BMC medical research methodology* 2014; **14**(1): 1-13.
6. Elbourne DR, Altman DG, Higgins JP, Curtin F, Worthington HV, Vail A. Meta-analyses involving cross-over trials: methodological issues. *International journal of epidemiology* 2002; **31**(1): 140-9.
7. Follmann D, Elliott P, Suh I, Cutler J. Variance imputation for overviews of clinical trials with continuous response. *Journal of clinical epidemiology* 1992; **45**(7): 769-73.
8. Balk EM, Earley A, Patel K, Trikalinos TA, Dahabreh IJ. Empirical assessment of within-arm correlation imputation in trials of continuous outcomes. 2013.
9. Ku HH. Notes on the use of propagation of error formulas. *Journal of Research of the National Bureau of Standards* 1966; **70**(4): 263-73.
10. Chaimani A, Higgins JPT, Mavridis D, Spyridonos P, Salanti G. Graphical tools for network meta-analysis in stata. *PLoS ONE* 2013; **8**(10): e76654.
11. Dias S, Welton NJ, Caldwell DM, Ades AE. Checking consistency in mixed treatment comparison meta-analysis. *Statistics in Medicine* 2010; **29**(7-8): 932-44.
12. Jackson D, Barrett JK, Rice S, White IR, Higgins JPT. A design-by-treatment interaction model for network meta-analysis with random inconsistency effects. 2014; **33**(21): 3639-54.
13. Guyatt G, Oxman AD, Akl EA, et al. GRADE guidelines: 1. Introduction—GRADE evidence profiles and summary of findings tables. *Journal of Clinical Epidemiology* 2011; **64**(4): 383-94.
14. Puhan MA, Schunemann HJ, Murad MH, et al. A GRADE Working Group approach for rating the quality of treatment effect estimates from network meta-analysis. 2014; **349**(sep24 5): g5630-g.
15. Brignardello-Petersen R, Bonner A, Alexander PE, et al. Advances in the GRADE approach to rate the certainty in estimates from a network meta-analysis. *Journal of Clinical Epidemiology* 2018; **93**: 36-44.
16. Brignardello-Petersen R, Murad MH, Walter SD, et al. GRADE approach to rate the certainty from a network meta-analysis: avoiding spurious judgments of imprecision in sparse networks. *Journal of Clinical Epidemiology* 2019; **105**: 60-7.
17. Brignardello-Petersen R, Mustafa RA, Siemieniuk RAC, et al. GRADE approach to rate the certainty from a network meta-analysis: addressing incoherence. *Journal of Clinical Epidemiology* 2019; **108**: 77-85.

© 2022 McGlynn ND et al. JAMA Network Open.
